# Measuring plantar load with STAMPS3D: a preliminary study on the impact of contoured orthoses

**DOI:** 10.3389/fbioe.2025.1648649

**Published:** 2025-11-20

**Authors:** Francesca Sairally, David A. Russell, Heidi J. Siddle, Kate Chauhan, Daniele Trinca, Claire Brockett, Pete Culmer

**Affiliations:** 1 School of Mechanical Engineering, Institute of Design, Robotics and Manufacturing, University of Leeds, Leeds, United Kingdom; 2 Leeds Vascular Institute, Leeds Teaching Hospitals NHS Trust, Leeds, United Kingdom; 3 Leeds Institute of Clinical Trials Research, University of Leeds, Leeds, United Kingdom; 4 Leeds Institute of Rheumatic and Musculoskeletal Medicine, University of Leeds, Leeds, United Kingdom; 5 Steeper Group, Leeds, United Kingdom; 6 Orthotics Department, Leeds Teaching Hospitals NHS Trust, Leeds, United Kingdom; 7 Biomedical Research Centre, Leeds Teaching Hospitals NHS Trust, Leeds, United Kingdom; 8 Insigneo Institute, School of Mechanical, Aerospace and Civil Engineering, University of Sheffield, Sheffield, United Kingdom

**Keywords:** diabetes, diabetic foot, plantar strain, shear, digital image correlation, 3D DIC, contoured orthoses, STAMPS3D

## Abstract

**Introduction:**

Diabetes-related foot disease including foot ulcers (DFU) are a growing concern with a huge associated socio-economic impact. Bespoke contoured orthoses are a common clinical intervention used to help prevent DFU formation in regions at risk through offloading, by implementing various design features such as arch support, cuts-outs, metatarsal pads and variable density materials. Research surrounding contoured orthoses has been limited to measuring plantar pressure to assess effectiveness and guide insole modifications necessary for individual patients. However, there is currently no in-shoe measurement tool capable of measuring all components of plantar load (plantar pressure and shear stresses).

**Methods:**

The STAMPS3D system has been developed to address this gap and has been successfully shown to measure the cumulative effect of plantar strain indicative of pressure and shear stresses that arise at the plantar interface. In this work, the STAMPS3D system has been used in a proof-of-concept study to explore the efficacy of capturing 3D strain data across a non-planar surface of different contoured orthoses.

**Results:**

Plantar strain patterns were shown to vary across anatomical regions of the foot, with statistically significant increases in plantar strain found for contoured conditions across the midfoot due to offloading associated with contoured orthoses. Differences in plantar strain were also observed across the toe and forefoot regions as a result of changing the material stiffness of the orthoses.

**Discussion:**

This work demonstrates the utility of employing 3D measurement to improve our understanding of plantar load under the influence of design features implemented in contoured orthoses and their ability to offload regions at risk of DFU formation. In doing so, improvements in management and prevention of diabetes-related ulceration can be made, tackling the social and financial costs associated.

## 1 Introduction

The global population with diabetes is a growing concern with an expected increase in prevalence from 537 million to 783 million from 2021 to 2045 (International Diabetes Federation, 2021). Consequently, a rise in associated diabetes-related foot health complications is also expected, with up to 25% of people with diabetes developing a diabetic foot ulcer (DFU) during their lifetime (Armstrong et al., 2017). DFUs place a significant burden on both patients and the healthcare system. For those who develop a DFU, roughly 20% will remain unhealed at 1 year, with 65% experiencing subsequent recurrence within 5 years after successful healing (Prompers et al., 2008; Armstrong et al., 2017). The mortality rate at 5 years is approximately 40%, increasing to more than 70% after amputation (Jupiter et al., 2016; Armstrong et al., 2017). The resulting financial cost is significant with estimates of up to £1 billion or 0.9% of the NHS England annual budget spent between 2014–2015 (Kerr et al., 2019).

To address the social and financial costs associated, a set of guidelines on the prevention and management of diabetic-related foot disease was developed by the International World Group on the Diabetic Foot (IWGDF) (International Working Group on the Diabetic Foot, 2023). Additionally, prevention was identified in 6/10 of the top diabetic foot research priorities set by the National Institute for Health and Care Research (NIHR) James Lind Alliance (NIHR, 2023). This has driven the development of assessment techniques used as a predictive tool for DFU formation, such as the emed® (Novel GmbH, Munich, Germany) pressure platform and Pedar® (Novel GmbH, Munich, Germany) in-shoe system. However, commercial measurement systems can be expensive making them less accessible for clinical use and are limited to capturing the pressure component of plantar load. Plantar load comprises of vertical (plantar pressure) and tangential (plantar shear stress) components. Increased plantar pressure has been reported in people with diabetes and even more so in those with existing DFUs, associating it with DFU formation (Veves et al., 1992; Chatwin et al., 2020). Although research on the influence of plantar pressure on DFU formation is extensive, peak plantar pressure alone is an unreliable marker for ulceration (Lavery et al., 2003; Chatwin et al., 2020). Veves et al. found that only 38% of ulcers were located at peak pressure sites, with others reporting that ulceration could still occur at pressures that are commonly considered normal (Veves et al., 1992; Perry et al., 2002). This suggests that there may be other factors contributing to the development of DFUs. Pollard et al. found that ulcer formation occurred at both peak pressure and peak shear sites but the dominant mechanical force of the two was unclear (Pollard and Le Quesne, 1983). A recent systematic review of 16 studies investigating plantar shear stress in people with diabetes showed that patients with an existing or pre-existing DFU displayed increased shear stress levels in contrast to those without ulceration (Jones et al., 2022). Although plantar pressure and plantar shear have been described as influential in DFU formation, the technical demands of capturing both components of plantar load simultaneously have limited the availability of appropriate measurement instrumentation, particularly for plantar shear. Within research settings measurement systems have begun to address this gap. Yavuz et al. developed a custom measurement platform with 80 embedded sensors, capable of capturing all components of plantar load (Yavuz et al., 2007; Yavuz, 2014). However, this technique is limited to capturing unshod conditions and therefore data is not representative of the plantar loads that occur while wearing shoes over a period of gait. More recently, in-shoe measurement devices have been reported capable of determining both plantar pressure and shear load components, employing electronic sensors either adhered to the plantar surface or imbedded in an insole, examples include (Lord and Hosein, 2000; Amemiya et al., 2016; Haron et al., 2024). Machine-learning techniques have also been used to enhance the measurements obtained from sensing elements within such in-shoe systems, demonstrating the potential to predict features such as tissue deformation, tissue injury risk and abnormalities occurring during gait (Hu et al., 2024; Xiang et al., 2024; Zhang et al., 2025). While these advances demonstrate the potential for combining real-time in-shoe plantar load measures with AI-based analysis, their spatial resolution is constrained by the number of sensing elements on the plantar surface (typically N = 4–8) and their positioning. Accordingly, our work at the University of Leeds has pursued an alternative approach for full-field plantar load characterization through development of the STrain Analysis and Mapping of the Plantar Surface (STAMPS) system (Jones et al., 2023). This technique comprises a multi-layer plastically deformable insole together with Digital Image Correlation (DIC) to record the cumulative plantar strain after a period of gait. This differs from sensor-based systems which capture real-time data, but this approach enables high spatial resolution of the data captured across the plantar surface. Additionally, this system allows for high adaptability compared to other devices, in which the size and shape of the insole can be tailored to user requirements. The STAMPS system initially used a 2D DIC approach, which has been successfully validated in a number of studies, including a study of 18 participants identifying a range of ‘normal’ values within a healthy cohort (Crossland et al., 2022; Jones et al., 2023; 2024). Use of STAMPS with 2D DIC showed the potential to characterise plantar load, but its application is limited to planar surfaces and therefore measures may not accurately record contoured surfaces of the foot. To address this limitation, we have developed STAMPS3D, enhancing the original STAMPS system with 3D DIC techniques to enable the capture of 3D strain measures (Sairally et al., 2025).

Currently, clinicians act to reduce risk of ulceration by offloading clinically indicated areas of high plantar load, e.g., site of callus or previous DFU. Plantar pressure measurements are used within research to measure the effect of interventions such as contoured orthoses. However, due to the complexities and time needed for data capture and analysis, the available pressure measurement systems are not routinely used in clinical practice. The insole design features for offloading vary, including arch support, shape, thickness, material stiffness or hardness and variable stiffness/density across an insole (Collings et al., 2020). Modifications can also be made through material cut-outs or addition of corrective pads (Ahmed et al., 2020; Collings et al., 2020). For insole designs that are implemented based on both pressure data and the individual’s foot shape, offloading was found to be more effective (Ahmed et al., 2020). In particular, improved forefoot offloading has been reported where multi-density, lower stiffness materials are used to make contoured orthoses, with increased arch support found to further reduce plantar pressures in the forefoot region (Ahmed et al., 2020). Overall, the literature indicates that offloading is more effective using contoured orthoses with custom arch supports in comparison to those that are completely flat, in addition to material stiffness being influential in how well the foot is offloaded. In cases where in-shoe pressure measurements were used to modify footwear (in particular to the insoles), together with high patient adherence, risk of DFU recurrence was reduced by more than 50% (Arts et al., 2015). Footwear modifications alone were found to relieve peak plantar pressures in local regions by 30%, however this varies among the literature with some showing conflicting results (Bus et al., 2011). This may be due to difficulty in distinguishing design features that have the greatest impact on offloading or other contributing factors that lack investigation, such as shear (Bus et al., 2008; 2011; Collings et al., 2020). Current literature focusses only on plantar pressure, hence the STAMPS3D system offers an approach that has the potential to measure plantar strain indicative of all components of strain at the orthosis-foot interface, to identify whether differences can be identified for insole design features such as material stiffness and arch support.

This paper presents a proof-of-concept study to investigate whether STAMPS3D can be used to capture 3D strain data across the orthosis-foot interface and to identify the impact of contoured orthoses varying in stiffness compared to normal flat conditions. By evaluating how orthosis design influences strain distribution, this work builds on previous research demonstrating the potential of STAMPS3D for DFU risk prediction and supports applications in preventative clinical strategies.

## 2 Materials and methods

A proof-of-concept study consisting of five healthy participants was conducted. Ethical approval was obtained from the University of Leeds Ethics Committee to conduct this study (EPS FREC-2024 1462-2517). Prior to assessment, participants read the participant information sheet and provided written consent. Participants were included if they were >18 years old and capable of walking unaided for 50 m. Individuals were excluded if they were diagnosed with diabetes or associated foot health conditions.

### 2.1 Insole fabrication

Foot impressions were taken by a qualified orthotist using a foam impression box. The impressions were scanned and used to create a 3D mesh, prior to 3D printing the bespoke contoured orthoses. Variation in the stiffness of the printed orthoses was achieved by adjusting the infill settings within a proprietary software, which uses a numeric scale to control infill density. This manufacturing process was conducted by an orthotics company (Steeper) that supply the NHS. For each participant, two pairs of contoured orthoses were printed defined as being either low (setting 1.3) or high (setting 5) stiffness. The stiffness of the insoles was controlled through the density of the print, temperature and speed of the 3D printer (Arkad P1, Qwadra, France), selected to ensure each insole type was significantly different. More detailed print-specific parameters such as temperature and head-speed are not directly accessible from this commercial software. An additional 1.5 mm Ethylene Vinyl Acetate (EVA) foam layer was added to the top of each insole as a standard feature for all contoured orthoses.

The development of the STAMPS system and the subsequent 3D DIC enhancement has previously been described (Jones et al., 2023; Sairally et al., 2025); however a brief overview is provided here for convenience. The STAMPS insole has a multi-layer structure. The 5 mm mid-layer is made of industrial plasticine, enabling plastic deformation and can be easily adapted to fit a range of shoe sizes and shapes. The top layer consists of a high contrast stochastic speckle pattern adhered to the surface using water-activated temporary tattoo paper. The speckle pattern was created using a commercial pattern generator (Correlated Solutions Inc.) and defined to have 65% density, 0.8 mm diameter and 75% variation. Pre- and post-walking image pairs with a resolution of 1920 × 1,200 were captured using a custom 3D camera setup consisting of two high resolution USB cameras (Basler, Germany, A2A 1920-160UCBAS). Image pairs were analysed using open-source 3D DIC software (DuoDIC) and post-processed further using custom scripts (MATLAB, Mathworks, United States). The raw DIC strain data was anatomically segmented using a masking protocol employed by a commercial plantar analysis software (PEDAR INC., Novel GmbH, Munich, Germany): Global, hallux, 2^nd^ toe, toes 3–5, 1^st^ Metatarsal Head (MTH), 2^nd^ MTH, 3^rd^ MTH, 4^th^ MTH, 5^th^ MTH, medial and lateral Midfoot, heel. The mask was scaled and rotated based on the insoles shape and size, enabling the strain data to be allocated to the corresponding anatomical regions. The strain measures calculated include the magnitude of strain (S_MAG_), anterior and posterior strain (S_ANT_, S_POST_), medial and lateral strain (S_MED_, S_LAT_) and the z-component of strain (S_Z_). While each of the strain components were captured and analysed, only the overall strain magnitudes, S_MAG_, are reported in the main text to provide a concise summary. Detailed directional strain data for each anatomical region and condition are available in Sections 1 and 2.1 of the Supplementary Material.

### 2.2 Experimental protocol

**FIGURE 1:**
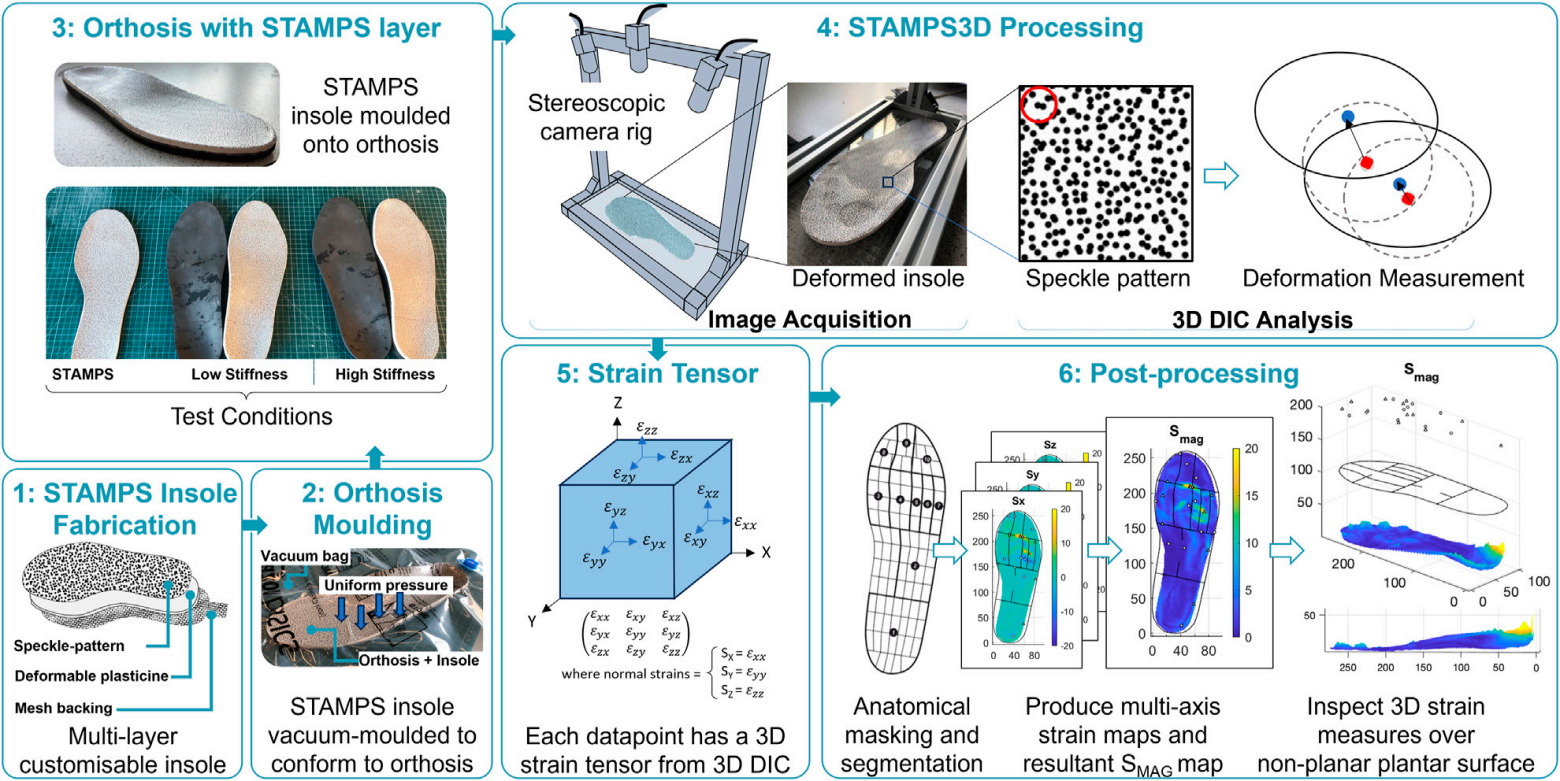
Overview of methods, STAMPS3D and post-processing of 3D strain data.

Participant shoe size was measured to ensure the STAMPS insoles and supportive neoprene boots (Ninewells Boot, Chaneco) were correctly sized. The STAMPS insoles were made >24 h prior to use. The STAMPS insoles were vacuum moulded to the orthoses surface, ensuring a consistent moulding approach and to avoid excessive deformation to the surface through manual moulding with hands. Any excess material was trimmed, and insoles were stored in a temperature-controlled cooler (15 °C) until use. Prior to testing, insoles were manually checked for full contact with the orthosis surface. A pre-walk image was captured of the insole and inserted into the right shoe, with a similarly sized insole inserted into the left shoe to prevent any unwanted difference in insole depth. For consistency, the right foot was used for measurements for each participant. Participants were required to walk along a 10 m walkway at a self-selected walking speed, ensuring a consistent number of steps were taken. In total, participants were asked to complete three walking conditions: 1. Low stiffness orthosis with STAMPS overlayed (contoured), 2. High stiffness orthosis with STAMPS overlayed (contoured) and 3. STAMPS alone (flat). The insole was carefully removed, and a post-walk image was captured. Each of the walking conditions were repeated three times, with a new STAMPS insole used for each assessment. Following the walking assessments using the STAMPS insole, a final walking assessment was performed using the Pedar® (Novel GmbH, Munich, Germany) in-shoe plantar pressure measurement system. Since the Pedar system cannot be used where there is pronounced and/or compound curvature (i.e., curvature in more than axis), direct comparisons between PPP and S_MAG_ will only be made with flat STAMPS condition. Measures of interest include the global and anatomical regions for median peak S_MAG_ and peak plantar pressure (PPP). Median measures were used to provide a more robust representation of data and less susceptible to being skewed by extreme measures. Figure 1 outlines the method setup using STAMPS3D, DIC analysis and post-processing.

### 2.3 Statistical analysis and post-processing

The data was assessed for normality using the Shapiro-Wilk test. The results indicated non-normal distribution across multiple anatomical regions and test conditions. Due to the small sample size (n = 5) and the repeated measures design, the non-parametric Friedman’s test was employed to compare plantar strain across the three test conditions. This test is appropriate for non-normally distributed data and does not assume homogeneity of variance. A post-hoc test with a Bonferroni correction was applied to adjust for multiple comparisons and identify which conditions showed statistically significant differences (P < 0.05). While the sample size of five participants limits the statistical power of the study, the repeated measures design and anatomical segmentation yielded a substantial amount of data per participant. The findings should be interpreted as preliminary, and future studies with larger cohorts are planned to confirm and expand the results. PPP for each anatomical region and globally were extracted using the multimask application (Novel, GmbH, Munich, Germany) with the mask previously described. Spearman’s correlation coefficient was used to assess the relationship between peak S_MAG_ and PPP, with a significant relationship being where a moderate and statistically significant correlation was found (0.4 < rho <0.69 and P < 0.05). This was consistent with the approach used in the healthy cross-sectional study using STAMPS (Jones et al., 2024). Median coefficients of variation (CV) of PPP and S_MAG_ was calculated for each region across all participants to determine repeatability of the study conditions.

## 3 Results

Five healthy participants were recruited and all successfully completed the walking assessments without reporting discomfort.

**FIGURE 2:**
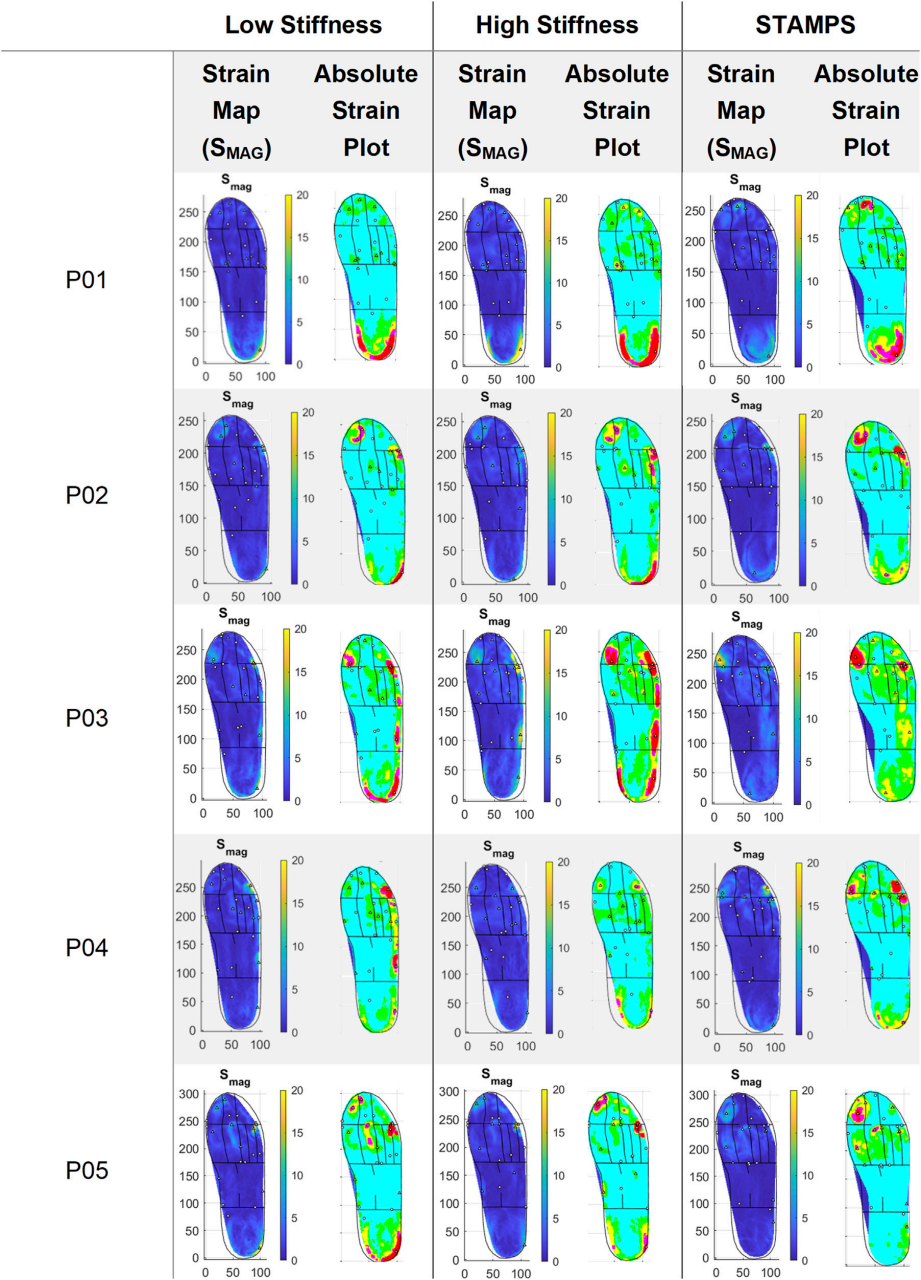
Representative strain maps for S_MAG_ across each participant and condition, with absolute plots demonstrating strain brackets: 0%–2.5% (blue), 2.5%–5% (green), 5%–7.5% (yellow), 7.5%–10% (magenta) and >10 (red).

Across all the participants, the global median peak S_MAG_ was 28.34% ranging from 19.97%–52.96%, 42.00% ranging from 21.19%–102.11% and 22.70% ranging from 16.03%–42.21% for the low stiffness, high stiffness and STAMPS conditions respectively. The global median PPP was 412.54 kPa, ranging from 293.00–542.00 kPa. The corresponding S_MAG_ strain maps and absolute plots displaying different coloured strain brackets across the plantar surface are shown in Figure 2. A single strain map representative of all three repeats for each condition and participant is displayed. The strain brackets were selected to better highlight local regions of high strain, e.g., 0%–2.5% (blue), 2.5%–5% (green), 5%–7.5% (yellow), 7.5%–10% (magenta) and >10 (red). The figure shows patterns across the participants, with high strain regions varying across the cohort. For the low stiffness condition, peak S_MAG_ was located at toes 3-5 in one participant, at the 5^th^ MTH in one and at the heel in three. For the high stiffness condition, peak S_MAG_ was located at the 4^th^ MTH in one participant, at the 5^th^ MTH in one and at the heel in three participants. For the flat condition, peak S_MAG_ was located at the hallux in one participant, at toes 3-5 in one, at the 4^th^ MTH in one, at the 5^th^ MTH in one and at the heel in one. PPP was located at the hallux in three participants, at the 2^nd^ MTH in one and at the heel in one. Peak S_MAG_ for all conditions and PPP occurred at the same region in one participant.

The region of peak S_MAG_ showed consistency across all repeats for all conditions in 1/5 participants. All other participants showed consistency across in two of the repeats for all conditions except for P05 during the flat condition. The median CV of S_MAG_ and PPP was computed across participants to compare across the three repeats. For the low stiffness condition, the median CV was 45%, for high stiffness was 30%, for the flat condition was 23% and for PPP was 5%.

**FIGURE 3:**
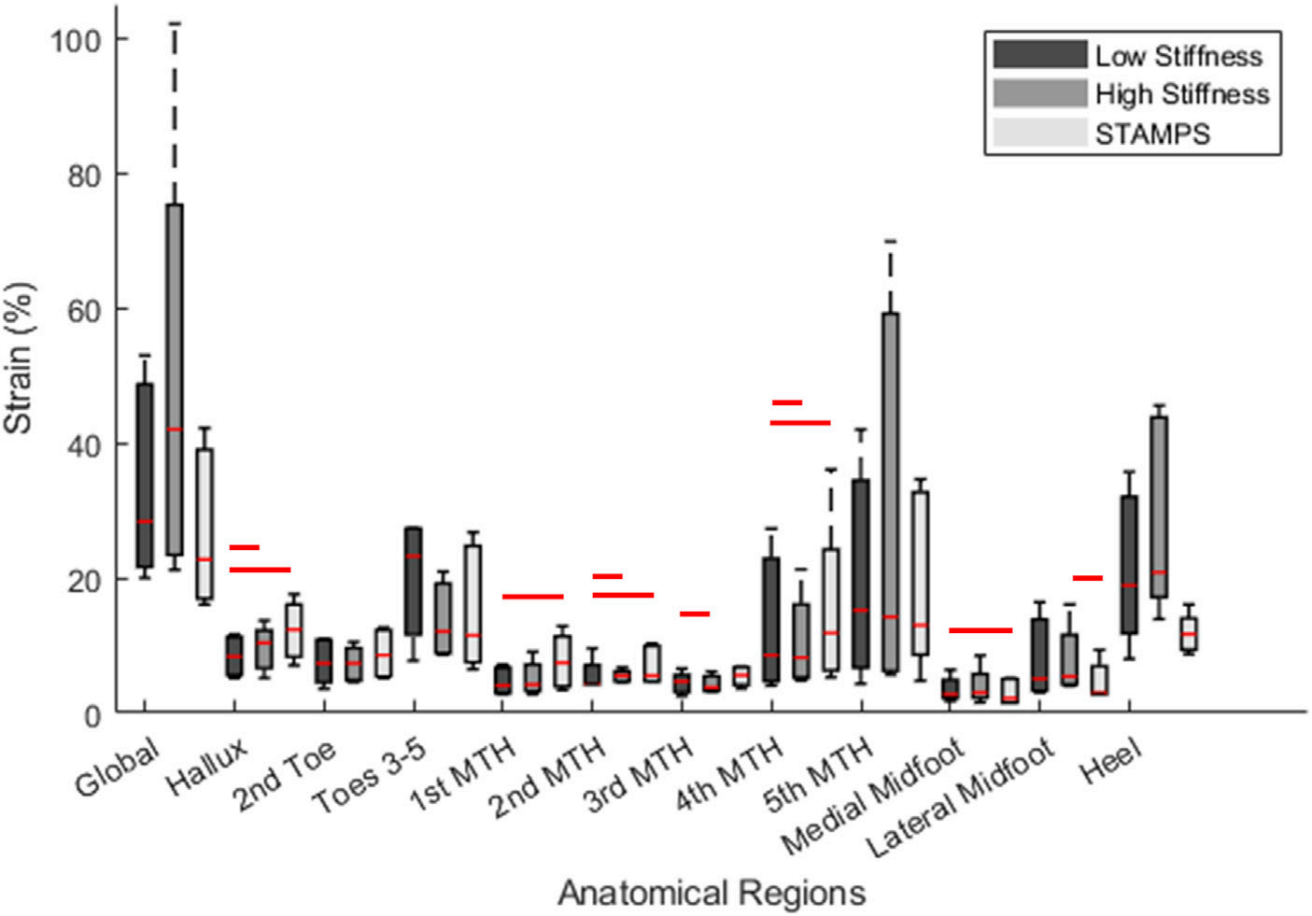
Boxplot demonstrating regional distribution of peak S_MAG_ across all participants for the low stiffness, high stiffness and STAMPS conditions. Red lines above boxplots represent significance (P < 0.05) between the corresponding conditions based on median measures. For detail regional breakdowns, see Section 2.2 of the Supplementary Material.

Median regional strain and PPP measures across the five participants are shown in Table 1. Across each condition the region of highest S_MAG_ and PPP were at different locations, being at toes 3–5 (23.22%), heel (20.80%), 5^th^ MTH (13%) and hallux (399.99 kPa) respectively. For the contoured conditions, the regions with highest S_MAG_ were the toes 3–5, 5^th^ MTH and heel, whereas for the flat condition S_MAG_ was highest at the hallux, 4^th^ MTH and 5^th^ MTH. The distribution of median peak S_MAG_ across each anatomical region for all participants is shown in Figure 3. Significant differences between conditions were found at the hallux, 1^st^ MTH, 2^nd^ MTH, 3^rd^ MTH, 4^th^ MTH, medial and lateral midfoot and heel. To complement the overview provided in Figure 3 and Section 2.2 of the Supplementary Material presents individual boxplots for each of the 12 anatomical regions, allowing for more in-depth interpretation of regional strain patterns across conditions.

**TABLE 1:** Regional median peak S_MAG_ for each condition and PPP across all participants, with interquartile range (IQR) reported in brackets.

Anatomical region	Low stiffness, peak S_MAG_ (%)	High stiffness, peak S_MAG_ (%)	STAMPS, peak S_MAG_ (%)	PPP (kPa)
Global	28.34 (21.59–48.71)	42.00 (23.40–75.30)	22.70 (16.90–39.00)	412.54 (328.31–517.13)
Hallux	8.37 (5.58–11.27)	10.38 (6.62–12.20)	12.36 (8.34–16.00)	399.99 (281.65–503.94)
2nd toe	7.35 (4.53–10.83)	7.36 (4.85–9.61)	8.56 (5.39–12.30)	140.75 (107.94–283.97)
Toes 3–5	23.22 (11.59–27.30)	12.10 (8.84–19.20)	11.50 (7.51–24.70)	86.33 (66.00–145.27)
1st MTH	4.06 (2.91–6.70)	4.20 (3.21–7.17)	7.45 (3.92–11.30)	210.08 (85.73–309.56)
2nd MTH	4.31 (4.24–7.11)	5.52 (4.66–6.15)	5.51 (4.68–9.92)	193.50 (149.26–248.10)
3rd MTH	4.67 (3.07–5.64)	3.77 (3.27–5.45)	5.60 (4.15–6.78)	173.33 (120.42–221.02)
4th MTH	8.56 (4.73–22.84)	8.21 (5.24–16.10)	11.85 (6.31–24.20)	173.33 (120.42–219.42)
5th MTH	15.21 (6.70–34.43)	14.20 (6.23–59.10)	13.00 (8.64–32.70)	97.63 (93.40–136.17)
Medial midfoot	2.77 (2.21–4.92)	3.01 (2.24–5.78)	2.16 (1.50–5.05)	23.92 (1.56–40.33)
Lateral midfoot	5.08 (3.30–13.84)	5.42 (4.21–11.60)	3.01 (2.76–6.94)	74.67 (58.57–148.79)
Heel	18.88 (11.78–32.04)	20.80 (17.10–43.80)	11.69 (9.37–14.00)	286.00 (208.13–364.07)

**FIGURE 4:**
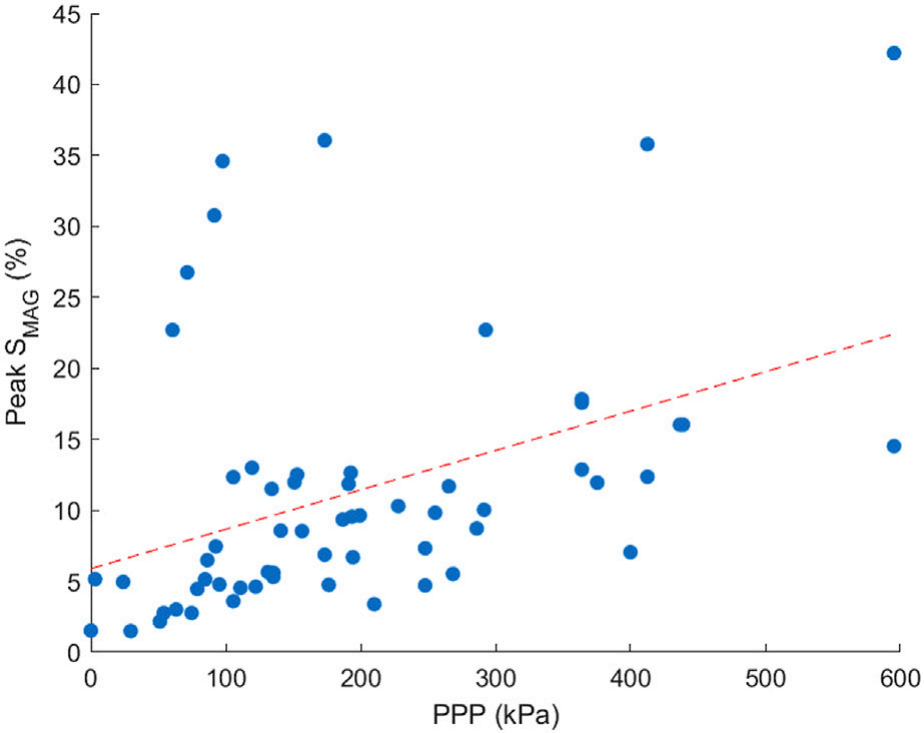
Scatter plot demonstrating the correlation between peak S_MAG_ and PPP across all participants. Points included are an average across three repeats for each region and participant.

Peak S_MAG_ and PPP were found to be moderately correlated, with PPP increasing with increased S_MAG_. Spearman’s correlation coefficient was found to be 0.52, P < 0.001. The relationship between the two plantar measures is demonstrated in Figure 4. For each participant there was moderate to strong correlation between peak S_MAG_ and PPP for 4/5 participants, ranging from 0.53–0.74. Only one participant showed a weak correlation between peak S_MAG_ and PPP (rho = 0.21). Regional correlation analysis between peak S_MAG_ and PPP revealed moderate to very strong positive correlations in 4 out of 12 regions. The remaining regions showed weak or inconsistent relationships. These findings may reflect limitations in pressure measurement systems sensor resolution, which varies across anatomical regions and may not fully capture localised strain patterns measured by STAMPS3D.

## 4 Discussion

This study has demonstrated the successful application of the STAMPS3D system to identify whether differences in plantar strain measures can be observed when an individual’s foot posture is altered using bespoke contoured orthosis compared to a standard flat insole. It also examined how plantar strain changes as a result of the material stiffness of the orthosis.

Plantar strain patterns were shown to vary across the foot between conditions in each participant (Figure 2), particularly between the contoured (low and high stiffness) and flat (STAMPS) conditions. The absolute plots highlight the edge of the heel and lateral side of the foot, indicating strain levels >10%. This differs for the flat condition where little to no high-level strain measures are located in those regions. For the flat condition of P01, the heel region is highlighted more centrally compared to the contoured conditions, which suggest that the raised edges of the heel and lateral side of the orthosis result in greater interaction with the individual’s foot, therefore increasing strain measures captured.

The relationship between PPP and peak S_MAG_, shown in Figure 4, was found to have significant moderate correlation (rho = 0.52, P < 0.001), aligning with results from a healthy cross-sectional study which also showed moderate correlation (rho = 0.65, P < 0.001) (Jones et al., 2024). Differences in correlation may be due to a smaller sample size in the present study. Although direct measures of in-shoe shear stress are not measured, the STAMPS3D system captures the resultant strain from both shear stress and plantar pressure at the foot’s surface interface. Therefore, the 3D strain data provides a proxy for assessment of plantar load under the tested conditions. Note that the reported correlation is between the peak S_MAG_ measures corresponding to the flat STAMPS3D condition and PPP, since plantar pressure comparison measurements could only be captured under flat conditions due to limitations of the Pedar system. The difference in resolution between STAMPS3D and pressure measurement insoles such as Pedar presents a challenge for regional correlation analysis. STAMPS3D captures strain at higher spatial resolution and across the full plantar surface, while Pedar relies on fewer sensors per region. This may lead to underrepresentation of pressure data in some areas, affecting the strength and reliability of regional correlations. Future studies may benefit from integrating higher-resolution pressure mapping or interpolative techniques to improve regional comparability.

For the low and high stiffness conditions, the highest S_MAG_ occurred at the same three regions including toes 3–5, 5^th^ MTH and heel (Table 1). Whereas for the STAMPS condition, the highest S_MAG_ occurred at the hallux, 4^th^ MTH and 5^th^ MTH, with the latter being the only region appearing in all three conditions with the highest measures. Within the toe region, the hallux and 2^nd^ toe regions showed lower S_MAG_ in the contoured conditions compared to the flat condition with statistical significance between low and STAMPS at the hallux (P = 0.003). Within the forefoot region, the 1^st^, 3^rd^ and 4^th^ MTH showed lower S_MAG_ measures in the contoured conditions compared to the flat condition. Strain measures were found to be significantly lower in the low stiffness condition compared to STAMPS across the 1^st^, 2^nd^ and 4^th^ MTH (P = 0.01, P = 0.006, P = 0.019). A number of studies found a decrease in plantar pressure in forefoot regions of those who worn contoured orthoses in comparison to a flat insole, with one finding a 34% pressure reduction at the 1^st^ MTH (Lord and Hosein, 1994; Bus et al., 2004; Collings et al., 2020). Since this study showed that peak S_MAG_ correlates with PPP, the decreased magnitude of strain reported may be as a result of a decreased pressure component. This can be found when contoured orthoses with a raised arch profile is present, enabling the forces to be redistributed from the front of the foot to the midfoot (Bus et al., 2004). This may explain why at the medial and lateral midfoot, the reported S_MAG_ is significantly increased in the contoured conditions (P = 0.003, P = 0.019). Similarly, S_MAG_ is increased at the heel for the contoured conditions. This again may be resulting from the raised edge prominent along the lateral side of the orthosis and around the heel cup, leading to increased resultant effect of shear and pressure in those regions as shown by the strain maps in Figure 2. In addition to this, these high strain regions may also be due to ill-fitted footwear since the footwear used in this study had to be able to accommodate for a range of sizes, leading to greater movement within the shoes and against the orthosis during gait. The regions where the highest PPP occurred were the hallux, 1^st^ MTH and heel. However, there were regions that reported relatively high peak S_MAG_ in comparison to PPP such as toes 3–5, 4^th^ MTH and 5^th^ MTH. Since the plantar pressure exhibited was low in the presence of high plantar strain, it suggests that these regions may have experienced elevated shear stress.

A comparison between the low and high stiffness conditions within the contoured group showed fewer differences. As mentioned previously, the highest median S_MAG_ occurred in the same three anatomical regions, however some significant differences were found at the hallux (P = 0.019), 2^nd^ MTH (P = 0.002), 3^rd^ MTH (P = 0.019), 4^th^ MTH (P = 0.019) and lateral midfoot (P = 0.019). At the hallux, 2^nd^ MTH and lateral midfoot the median peak S_MAG_ was found to be significantly higher in the high stiffness group, whereas at the 3^rd^ and 4^th^ MTH significantly increased S_MAG_ was found in the low stiffness group. From Table 1, measures reported in the medial forefoot and toe regions appear to be generally reduced when low stiffness insoles were worn compared to the lateral toe and forefoot region, even though the arch profile was consistent in both contoured conditions. This suggests that differences in the distribution of plantar load may occur in differing regions depending on the stiffness of insole used. From the literature, multiple papers have reported differences in plantar pressures depending on insole material stiffness, hardness or density (Tang et al., 2015; Nouman et al., 2019; Ahmed et al., 2020; Shi et al., 2022). However, whether the pressure reductions are found in the lower or upper margin of these material properties (i.e., low or high stiffness) are conflicted among studies. Therefore, from the current data it is difficult to determine if these results are significant and whether pressure or shear stress has greater influence in these regions depending on material stiffness. It is also worth highlighting that since this study consists of a healthy cohort, the expected impact of using a low or high stiffness insole may not be as pronounced in comparison to a cohort with diabetes-related foot complications. This could be due to a ‘healthy’ individual having relatively normal distribution of plantar load compared to those with foot issues.

The key limitations of the STAMPS3D system are similar to those of the 2D STAMPS system which have previously been discussed and relates to measurement capability, variability in outcomes and clinical utility (Jones et al., 2023; Sairally et al., 2025). Most notably, the STAMPS measurement approach involves a trade-off between temporal measurement frequency and spatial resolution; using DIC to record the cumulative strain incurred through gait inherently precludes the ability to capture real-time or dynamic data but does allow for high resolution measurement across the full plantar surface. This differentiates the STAMPS (2D and 3D) systems from electronic-sensor based in-shoe systems which are capable of real-time measurement but lack spatial resolution and full coverage. Both approaches have virtue and there is potential to use them together as complementary technologies to obtain a more complete understanding of plantar loading. In this study, the outcome measures obtained from STAMPS3D exhibited a relatively high coefficient of variation across gait repeats. However, this level of variability is consistent with previous STAMPS2D in-shoe testing, where natural gait variation contributed to higher CVs compared to bench testing (Jones et al., 2023; Jones et al., 2024). Notably, regions with lower absolute strain tended to show higher CVs, which may exaggerate variability without reflecting clinically meaningful differences. In prior studies involving diabetic participants, regions with higher strain magnitudes were associated with lower CVs (The Leeds Teaching Hospitals NHS Trust, 2023), suggesting improved measurement stability under clinically relevant load regimes and contexts, and an aspect that will be pursued in future work. The STAMPS systems have been co-developed with clinical stakeholders with the aim of being appropriate for clinical use in the future. In its current form, STAMPS3D system does have some limitations in this respect; although data capture can be completed within 10–15 min (deemed clinically appropriate), it requires additional preparation time (to mould insoles to the contours of the orthosis) and to perform the post-hoc analysis steps (including DIC, masking and determining summary metrics). Addressing these aspects is an important part of our future work, firstly to optimise the fitting process and secondly to automate the analysis process guided by expert clinical input, combining to produce a streamlined process suitable for use within clinical sessions.

This study was conducted with healthy participants to establish the baseline performance and repeatability of the STAMPS3D system. While this does limit extrapolation to understand potential clinical efficacy, it is an important first step in evaluating system feasibility prior to future clinical studies. Using this approach, our previous work using STAMPS2D demonstrates greater strain differentiation in diabetic populations (The Leeds Teaching Hospitals NHS Trust, 2023) suggesting that STAMPS3D may offer similar capabilities coupled with the ability to evaluate non-planar insoles. Future clinical studies involving participants with diabetes (including those with neuropathy and foot deformities), are planned to evaluate the clinical utility of STAMPS3D. In doing so, the effect of bespoke orthoses and the various design features used to offload regions at risk of DFU formation in patients with diabetes, can be better understood and used to improve management and prevention procedures.

In conclusion, this proof-of-concept study demonstrates the potential of the STAMPS3D system to capture 3D strain patterns indicative of plantar loads at the foot-orthosis interface. The system was successfully applied to compare strain patterns across flat and contoured orthoses of varying stiffness, revealing significant differences in local strain distribution. Notably, regions of high plantar strain did not always align with regions of high plantar pressure, suggesting the influence of shear stress components. While these findings are promising, the repeatability and clinical applicability of STAMPS3D requires further investigation. The current study was limited to healthy participants, and future work will involve individuals with diabetes, including those with neuropathy and foot deformity, to assess robustness and relevance in clinical settings. These efforts aim to improve our understanding of orthotic intervention and their role in preventing diabetic foot ulceration.

## Data Availability

The datasets generated for this study can be found in the Research Data Leeds Repository (https://doi.org/10.5518/1706).
