# Supplementary material for: Measuring plantar load with STAMPS3D: a preliminary study on the impact of contoured orthoses
Source: Front Bioeng Biotechnol. 2025 Nov 20;13:1648649. doi: 10.3389/fbioe.2025.1648649 (PMC12675326; doi:10.3389/fbioe.2025.1648649)
Supplement: Supplementary file 1 [file DataSheet1.docx]

Supplementary Material

# Supplementary Tables

**Supplementary Table 1.** Regional median peak S_X_ for each condition across all participants, with interquartile range (IQR) reported in brackets.

| **Anatomical Region** | **Low Stiffness, Peak S_X_ (%)** | **High Stiffness, Peak S_X_ (%)** | **STAMPS, Peak S_X_ (%)** |
| --- | --- | --- | --- |
| Global | 18.09  (10.51-21.88) | 12.01  (9.80-43.45) | 18.58  (9.26-24.76) |
| Hallux | 4.00  (2.20-8.19) | 5.33  (2.90-8.61) | 5.83  (2.64-8.47) |
| 2^nd^ Toe | 3.98  (2.58-4.36) | 3.72  (2.76-4.80) | 3.97  (2.41-4.72) |
| Toes 3-5 | 6.92  (5.55-13.58) | 5.12  (4.52-8.81) | 5.97  (3.65-16.85) |
| 1^st^ MTH | 1.33  (1.08-2.97) | 1.01  (0.57-2.30) | 1.88  (1.24-6.56) |
| 2^nd^ MTH | 1.60  (0.87-2.42) | 1.08  (0.63-1.24) | 2.19  (1.63-4.36) |
| 3^rd^ MTH | 1.08  (0.87-1.91) | 0.94  (0.48-1.58) | 2.03  (1.12-2.18) |
| 4^th^ MTH | 1.80  (0.72-3.19) | 1.53  (0.84-2.01) | 2.10  (1.41-5.38) |
| 5^th^ MTH | 7.26  (3.49-13.99) | 10.69  (2.02-29.64) | 10.94  (3.92-24.10) |
| Medial Midfoot | 1.48  (0.93-3.23) | 1.96  (1.11-2.65) | 0.86  (0.80-2.66) |
| Lateral Midfoot | 3.12  (1.68-4.33) | 2.44  (1.58-3.41) | 0.81  (0.75-2.11) |
| Heel | 6.81  (4.04-16.88) | 8.64  (4.58-19.55) | 4.75  (3.79-7.87) |

**Supplementary Table 2.** Regional median peak S_Y_ for each condition across all participants, with interquartile range (IQR) reported in brackets.

| **Anatomical Region** | **Low Stiffness, Peak S_Y_ (%)** | **High Stiffness, Peak S_Y_ (%)** | **STAMPS, Peak S_Y_ (%)** |
| --- | --- | --- | --- |
| Global | 14.31  (11.21-39.14) | 32.24  (11.86-54.65) | 15.75  (10.61-28.45) |
| Hallux | 5.55  (4.87-7.94) | 6.58  (5.67-8.65) | 10.62  (7.13-12.98) |
| 2^nd^ Toe | 4.17  (2.87-7.48) | 3.95  (3.69-6.76) | 4.53  (4.05-9.63) |
| Toes 3-5 | 9.71  (6.92-25.23) | 9.73  (4.37-16.29) | 6.64  (2.36-13.68) |
| 1^st^ MTH | 2.09  (0.55-4.14) | 3.28  (1.72-5.22) | 4.98  (2.72-9.68) |
| 2^nd^ MTH | 3.90  (3.37-6.27) | 4.52  (4.21-5.58) | 4.89  (4.22-7.78) |
| 3^rd^ MTH | 2.42  (1.44-5.09) | 3.52  (1.37-4.47) | 2.00  (1.57-4.54) |
| 4th MTH | 8.02  (3.28-22.45) | 5.68  (4.55-14.93) | 11.72  (2.69-21.36) |
| 5^th^ MTH | 7.69  (5.42-33.06) | 5.18  (4.51-53.62) | 10.12  (3.74-13.70) |
| Medial Midfoot | 1.54  (1.04-2.47) | 1.76  (1.44-4.27) | 0.51  (0.43-1.02) |
| Lateral Midfoot | 3.25  (2.35-13.16) | 4.64  (3.03-10.72) | 2.86  (1.83-6.54) |
| Heel | 12.91  (7.56-14.72) | 11.48  (9.48-22.18) | 8.00  (6.84-10.87) |

**Supplementary Table 3.** Regional median peak S_Z_ for each condition across all participants, with interquartile range (IQR) reported in brackets.

| **Anatomical Region** | **Low Stiffness, Peak S_Z_ (%)** | **High Stiffness, Peak S_Z_ (%)** | **STAMPS, Peak S_Z_ (%)** |
| --- | --- | --- | --- |
| Global | 6.73  (4.68-7.41) | 7.41  (5.32-9.80) | 1.92  (1.23-2.09) |
| Hallux | 0.43  (0.23-0.99) | 0.33  (0.26-0.95) | 0.61  (0.29-1.04) |
| 2^nd^ Toe | 0.43  (0.26-0.45) | 0.33  (0.27-0.44) | 0.35  (0.26-0.51) |
| Toes 3-5 | 0.90  (0.58-1.36) | 0.56  (0.45-0.87) | 0.59  (0.35-1.71) |
| 1^st^ MTH | 0.24  (0.22-0.52) | 0.21  (0.07-0.55) | 0.24  (0.13-0.75) |
| 2^nd^ MTH | 0.22  (0.15-0.31) | 0.17  (0.08-0.27) | 0.24  (0.18-0.47) |
| 3^rd^ MTH | 0.16  (0.12-0.28) | 0.12  (0.09-0.27) | 0.20  (0.12-0.23) |
| 4^th^ MTH | 0.27  (0.10-0.38) | 0.25  (0.11-0.27) | 0.23  (0.18-0.53) |
| 5^th^ MTH | 0.75  (0.41-1.69) | 1.21  (0.25-3.36) | 1.15  (0.42-2.00) |
| Medial Midfoot | 0.40  (0.33-0.91) | 0.39  (0.28-1.49) | 0.11  (0.09-0.29) |
| Lateral Midfoot | 0.54  (0.34-0.72) | 0.52  (0.42-0.86) | 0.08  (0.08-0.23) |
| Heel | 6.73  (4.68-7.41) | 6.02  (5.12-9.50) | 1.04  (0.80-1.43) |

# Supplementary Figures

## Strain Maps

|  | **Low Stiffness** | | **High Stiffness** | | **STAMPS** | |
| --- | --- | --- | --- | --- | --- | --- |
|  | **Strain Map (S_MAG_)** | **Absolute Strain Plot** | **Strain Map (S_MAG_)** | **Absolute Strain Plot** | **Strain Map (S_MAG_)** | **Absolute Strain Plot** |
| P01 | 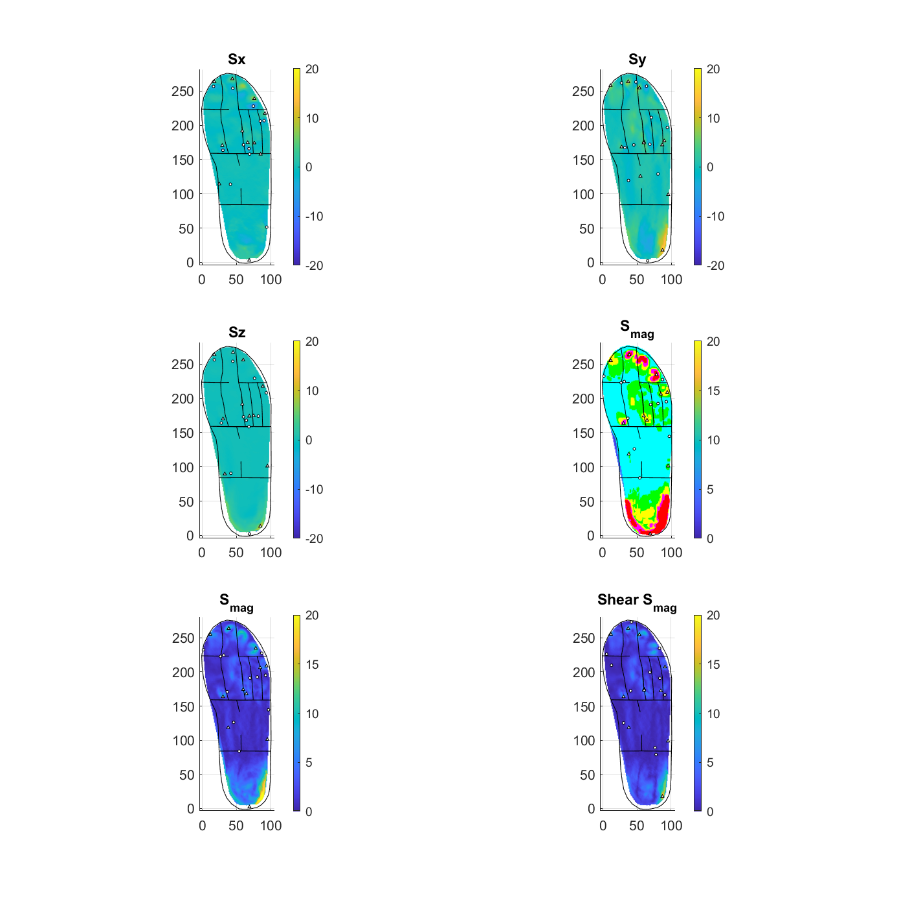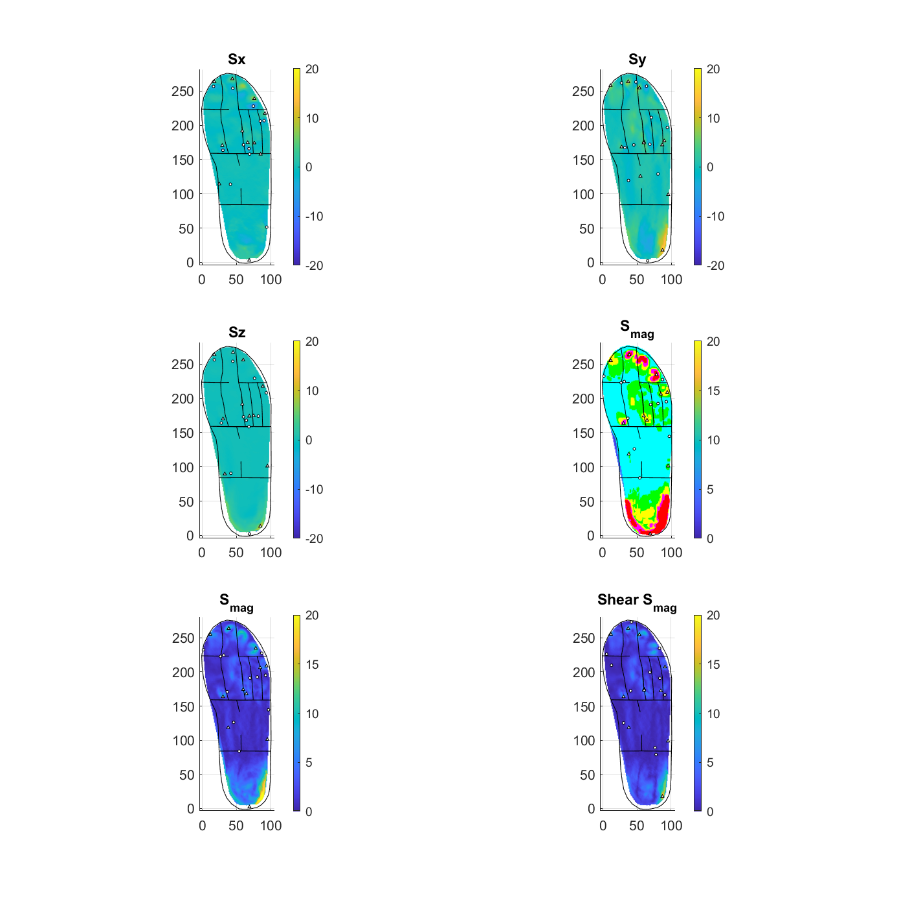 | | 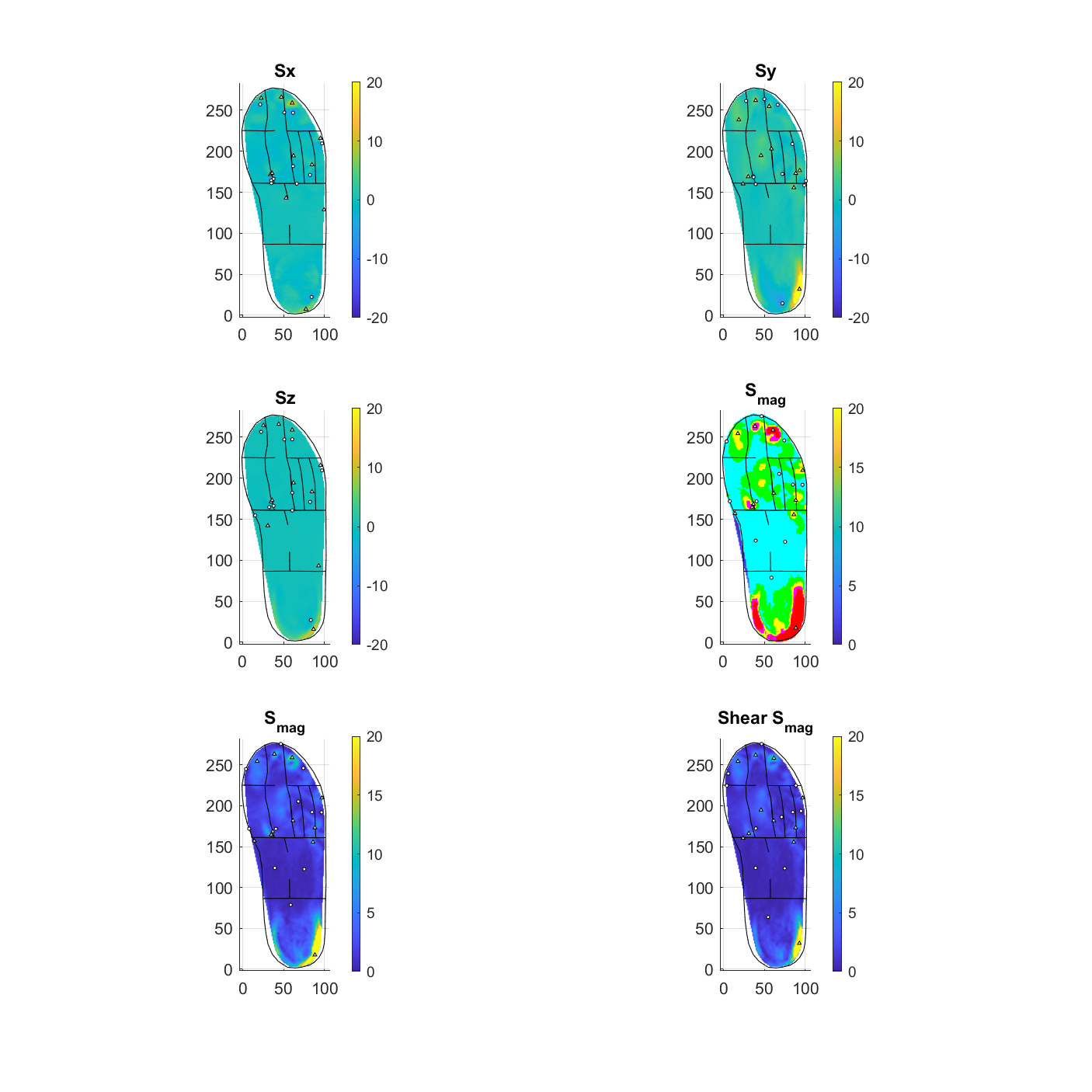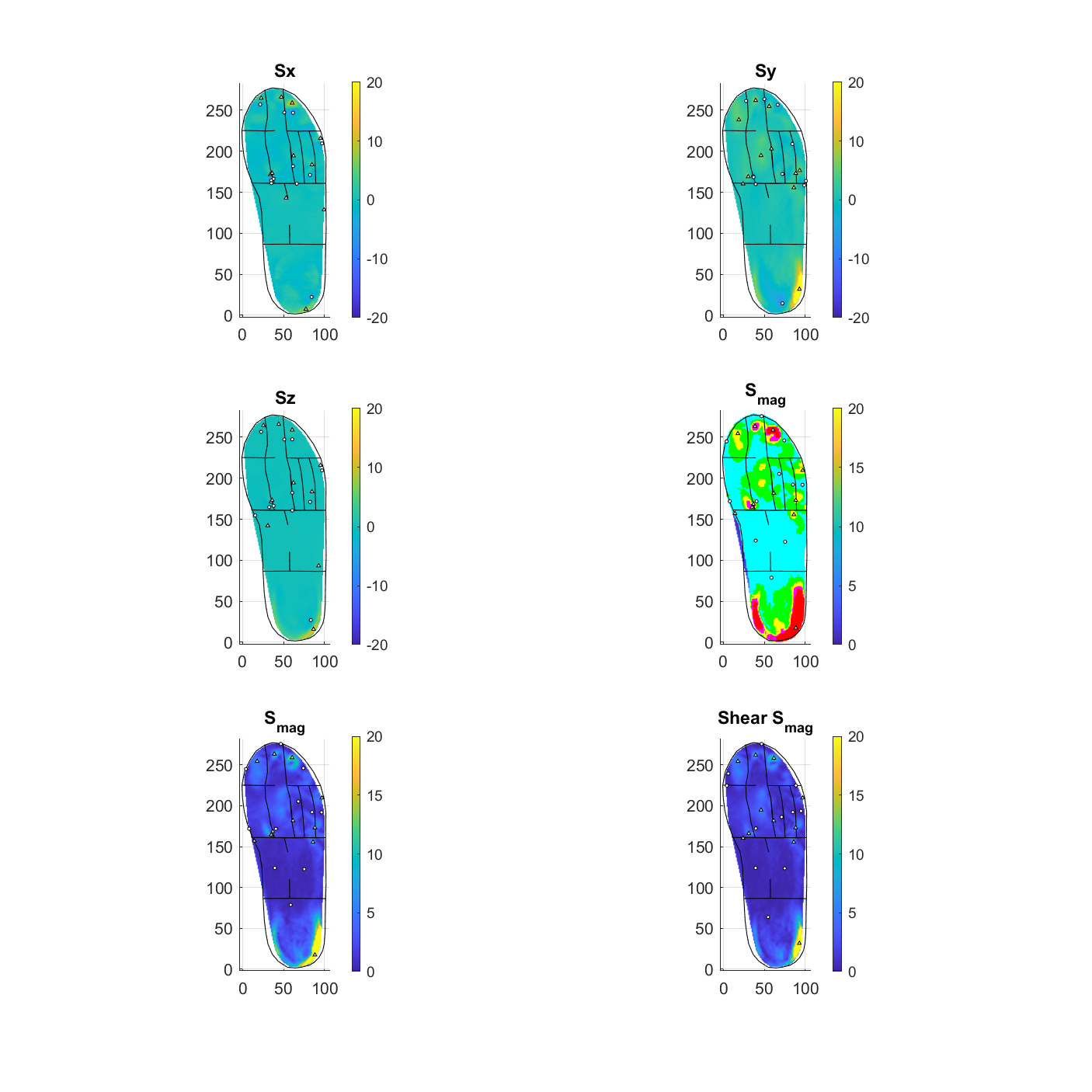 | | 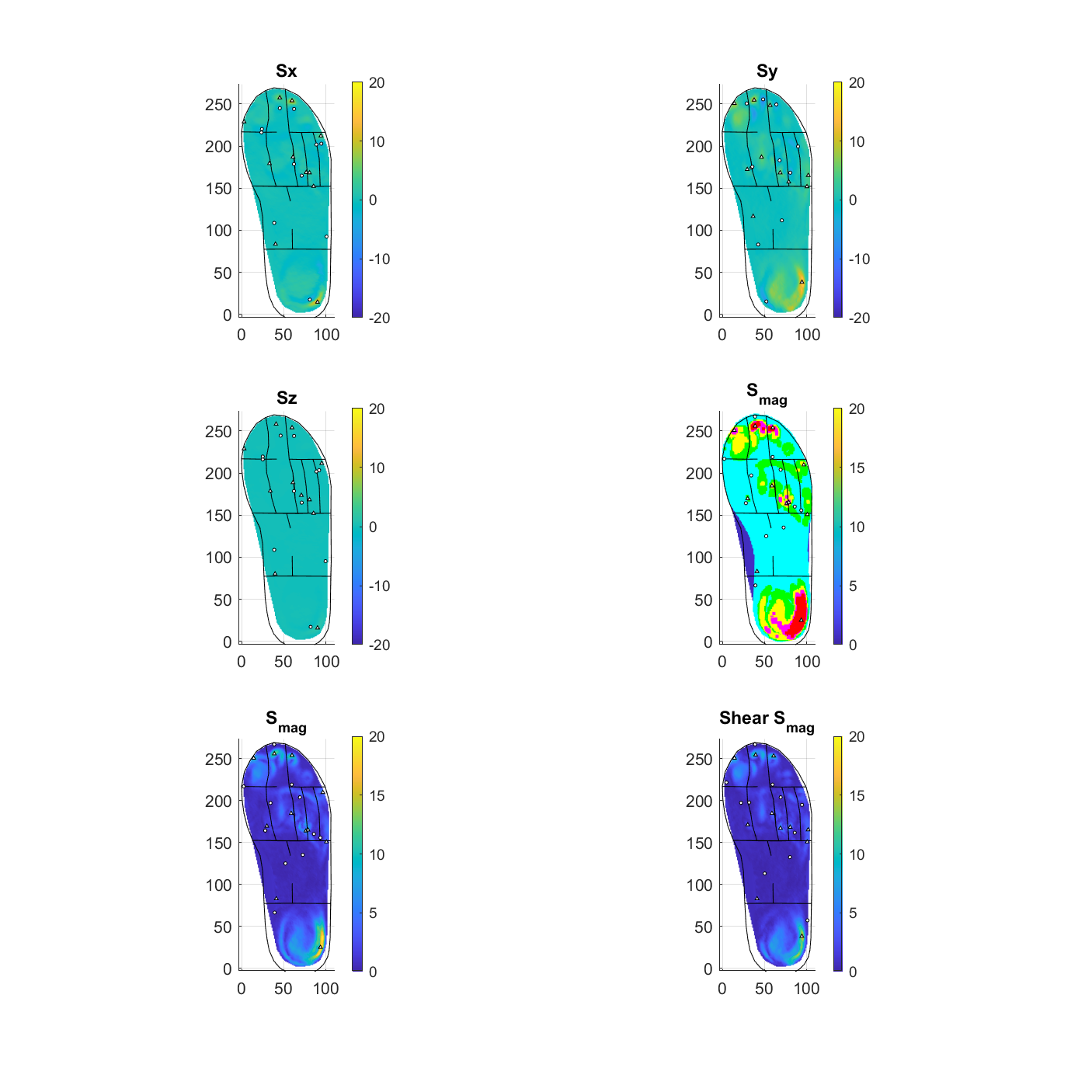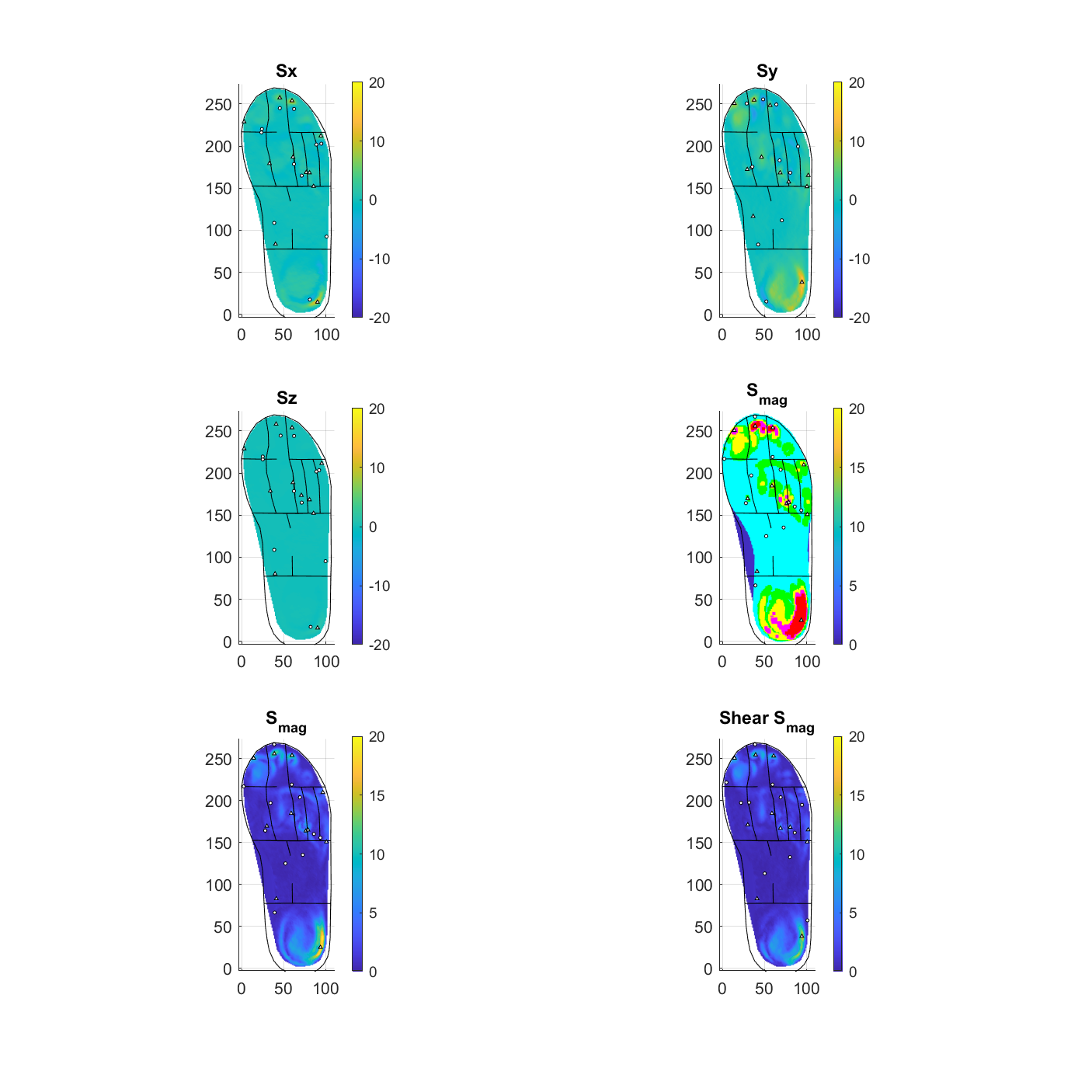 | |
| P02 | 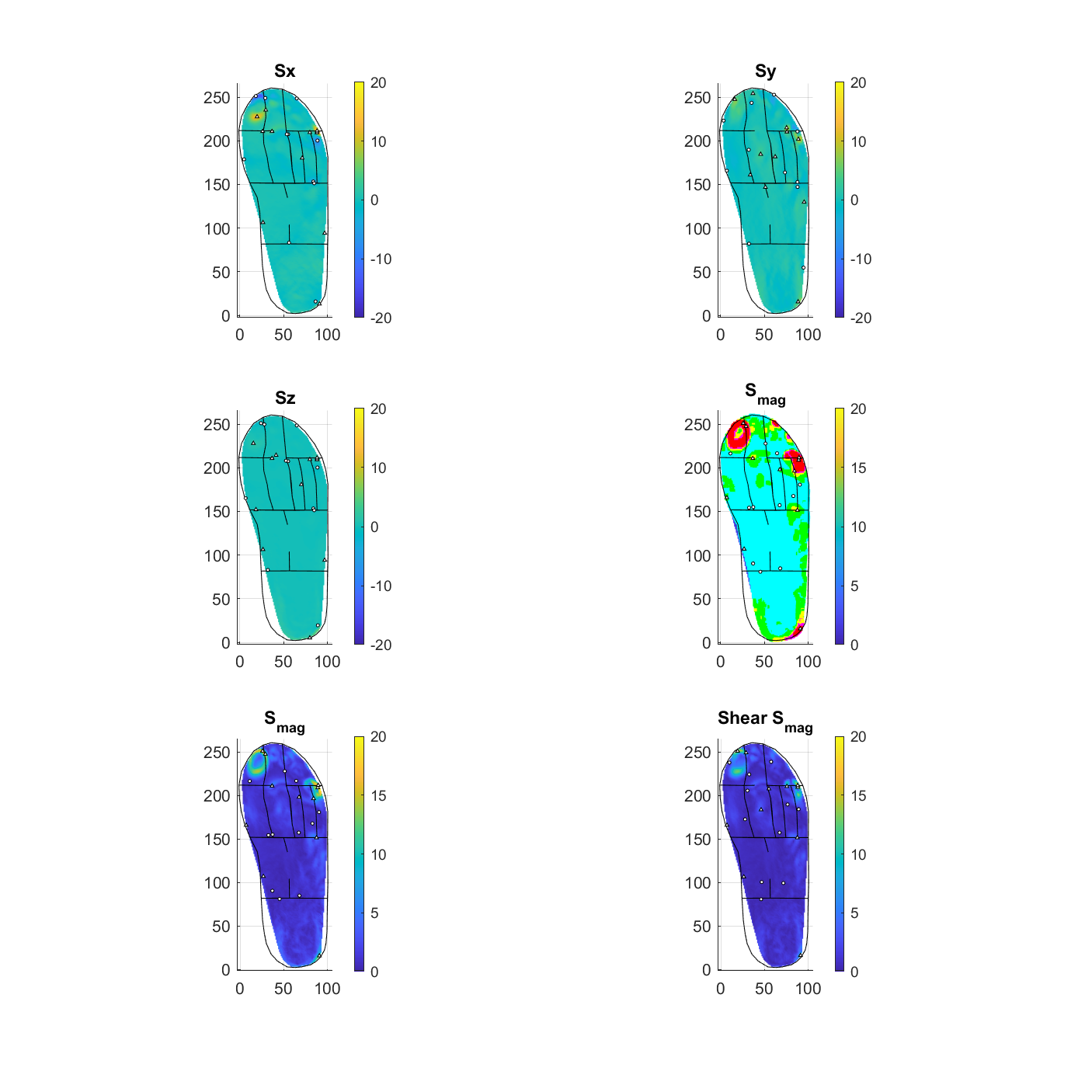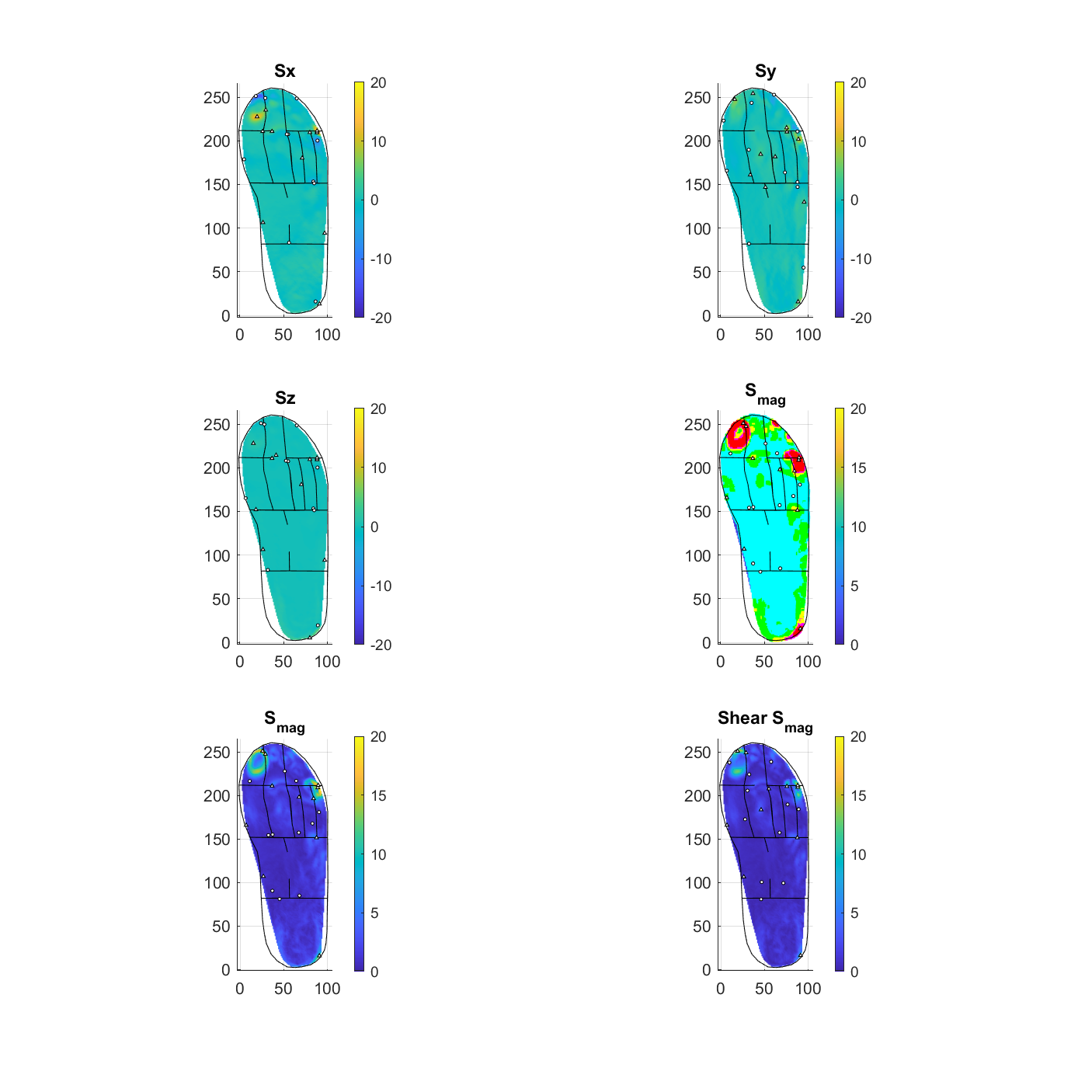 | | 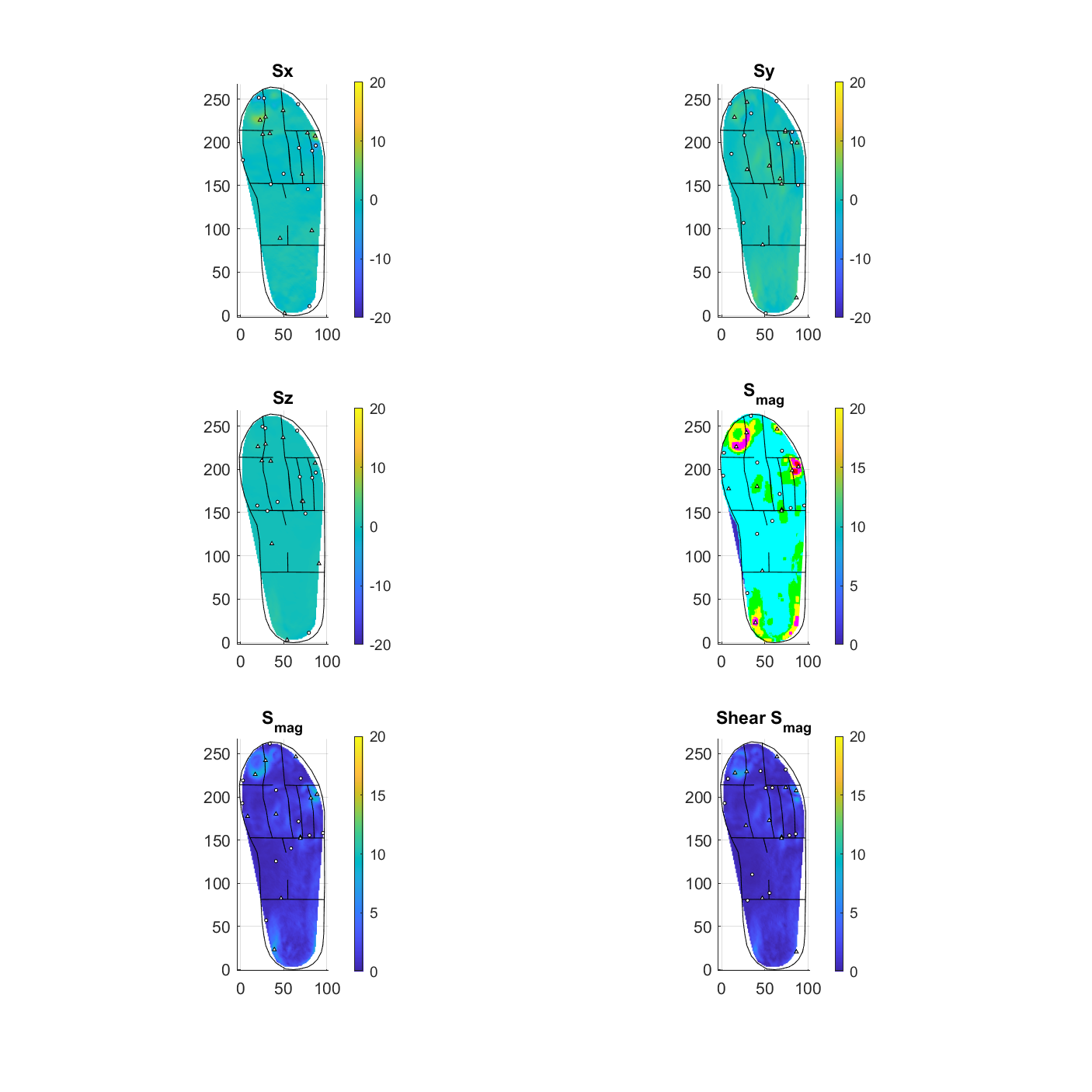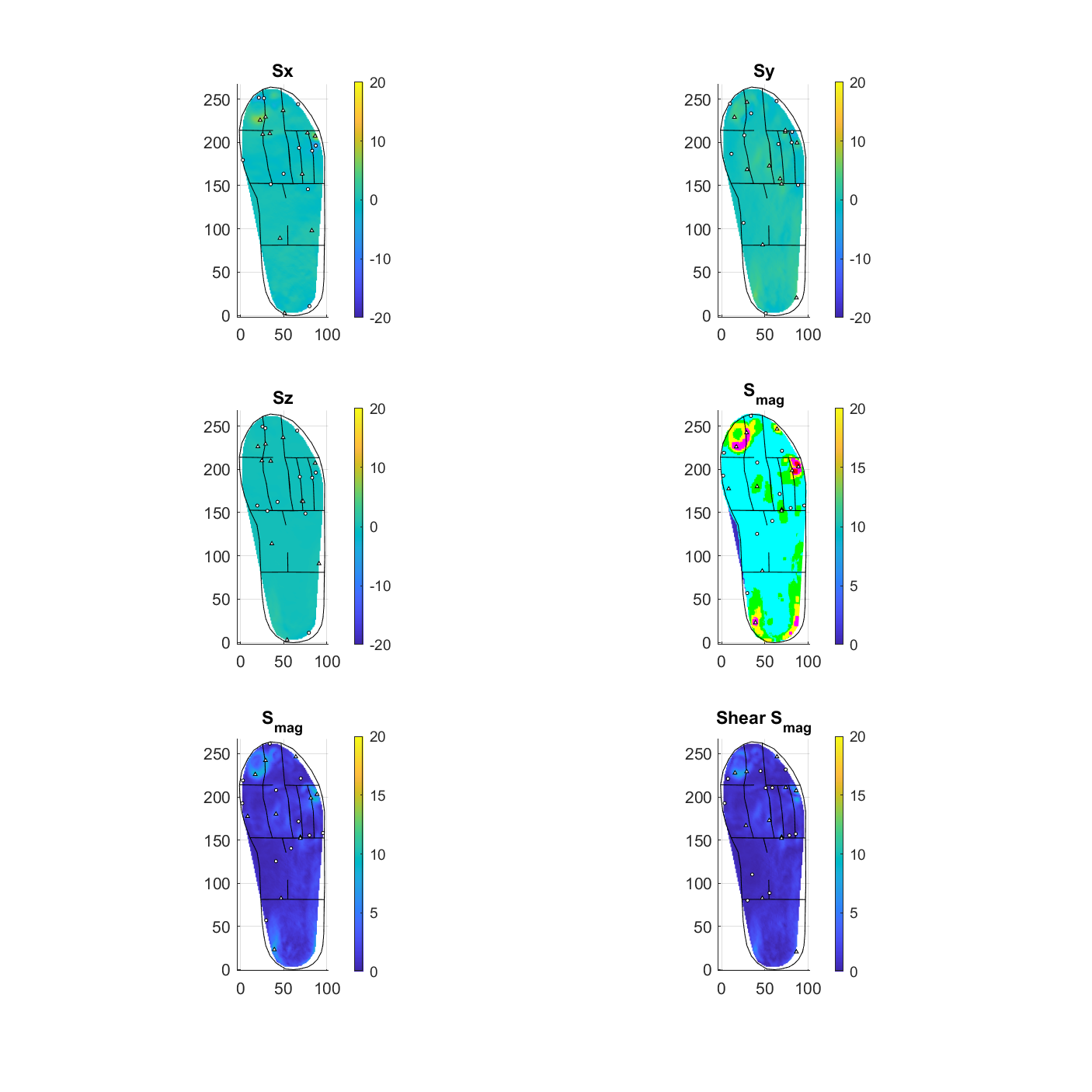 | | 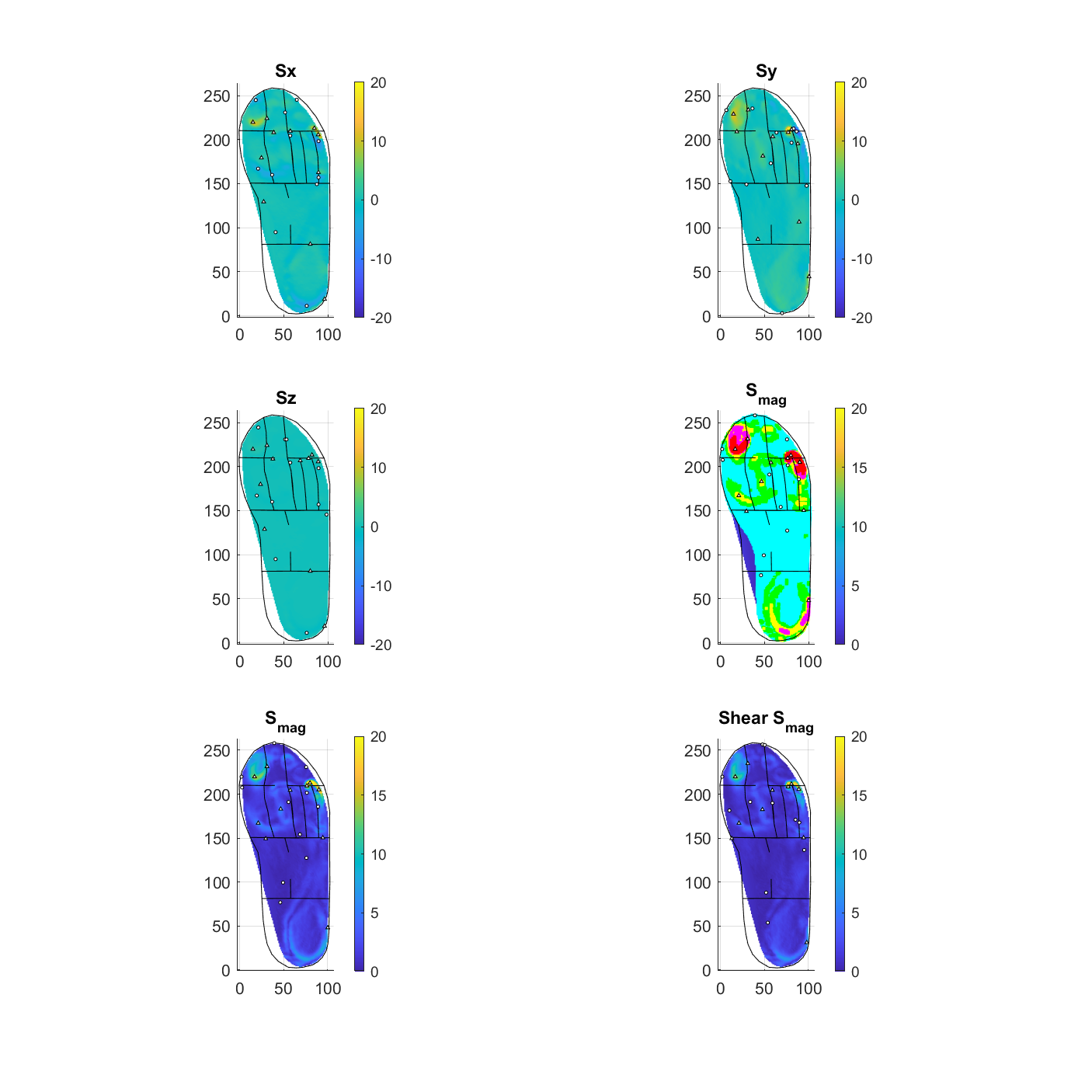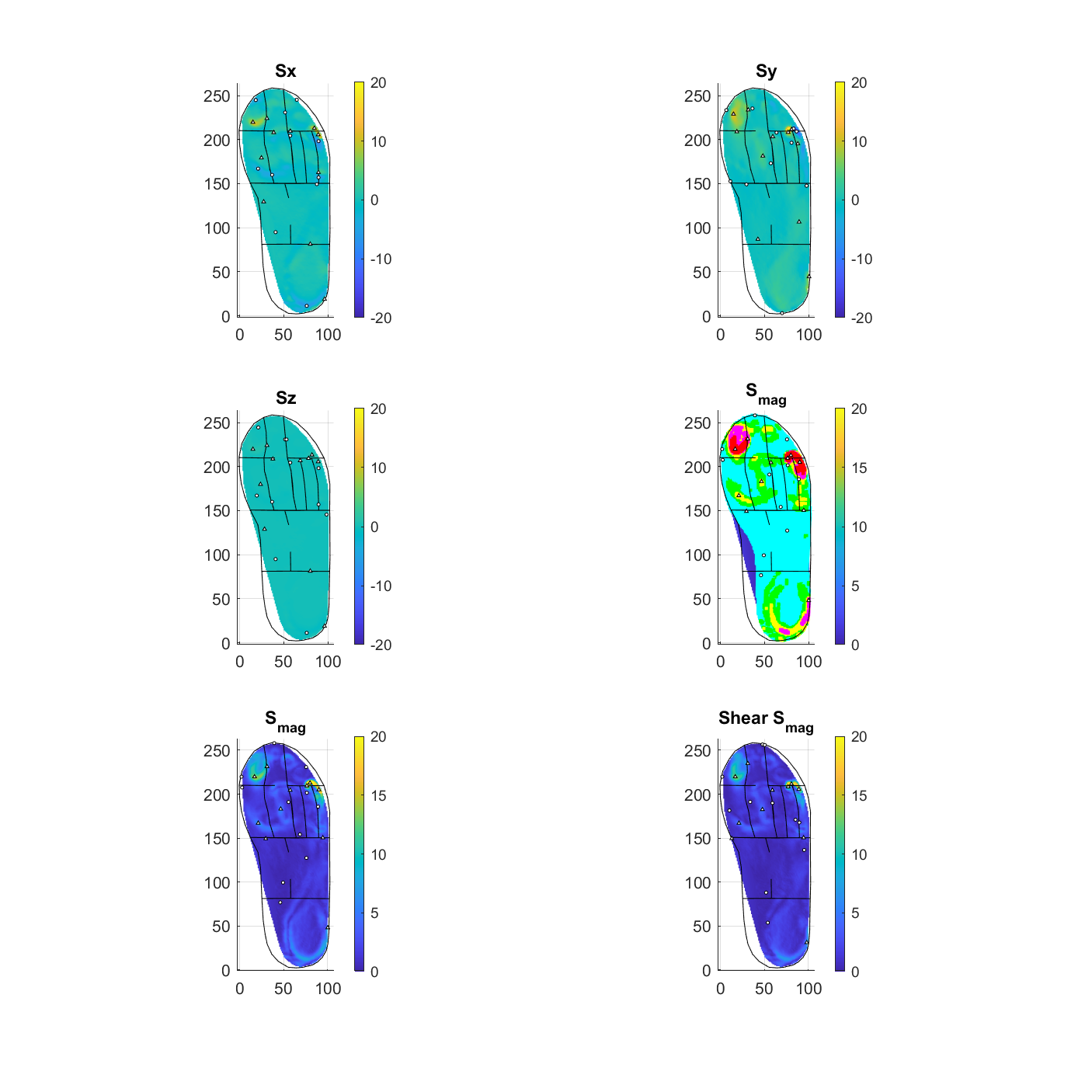 | |
| P03 | 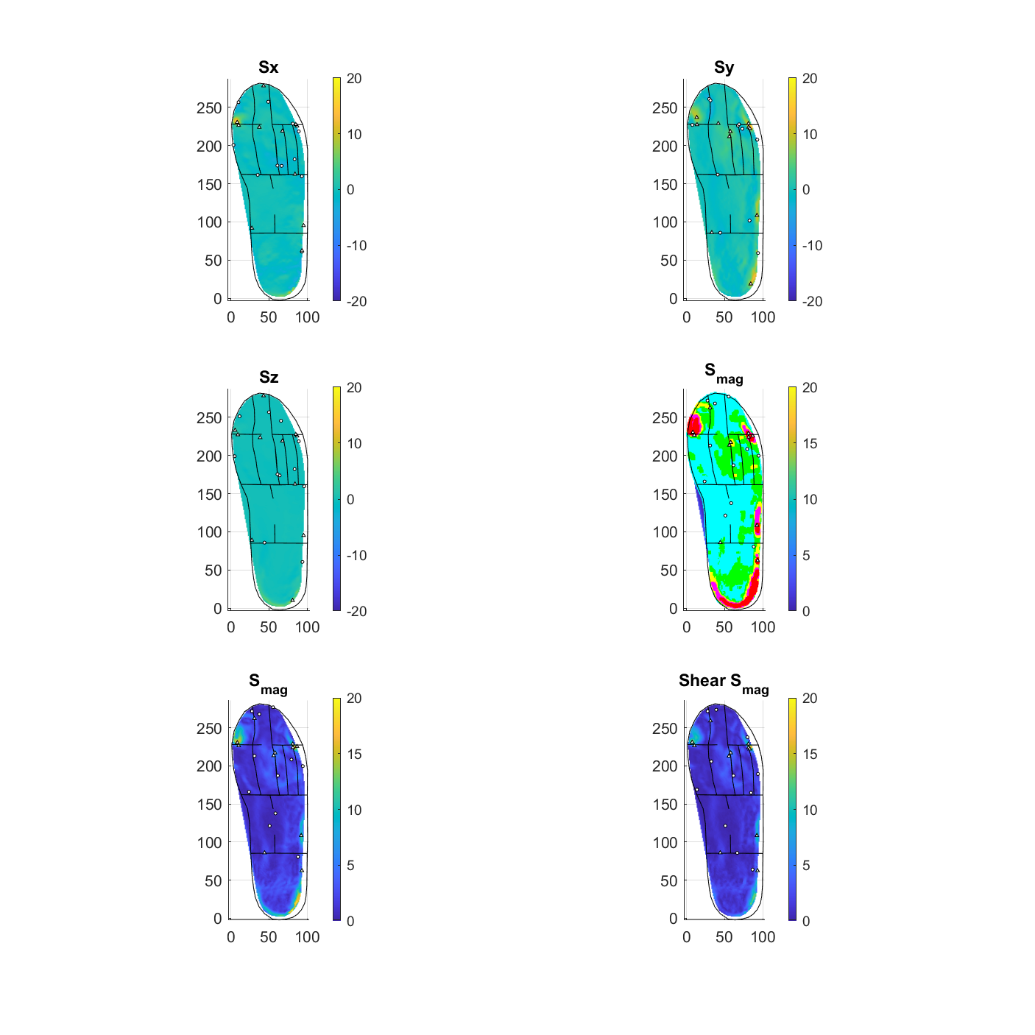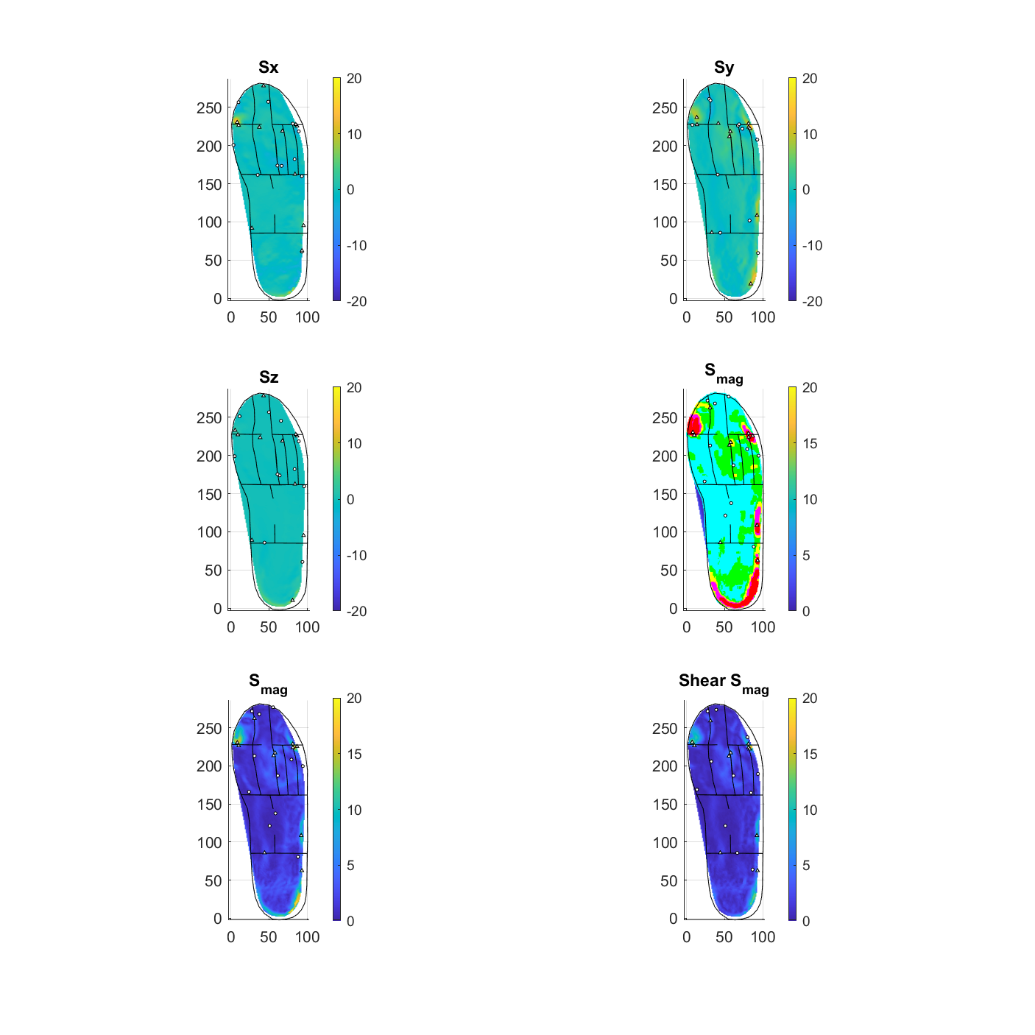 | | 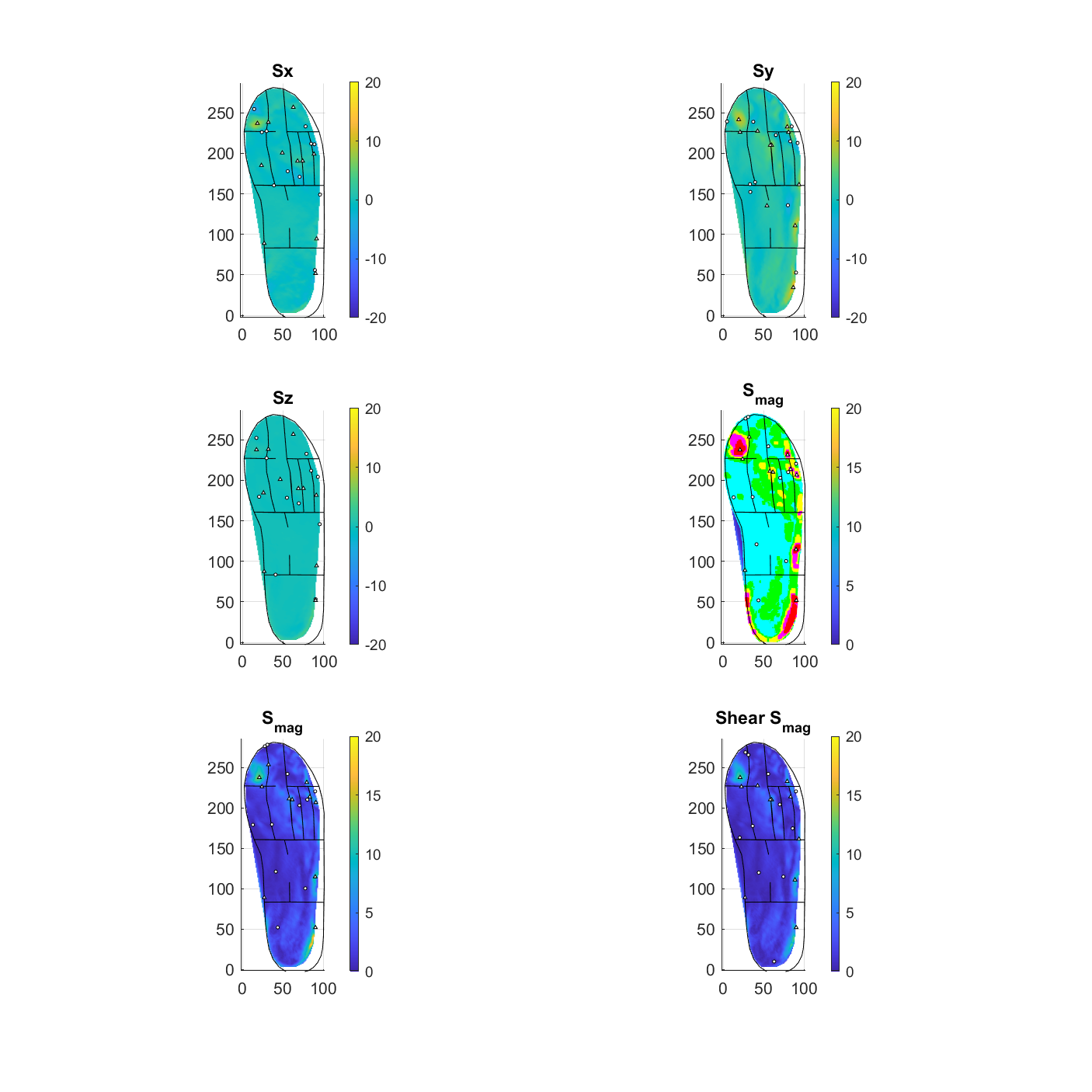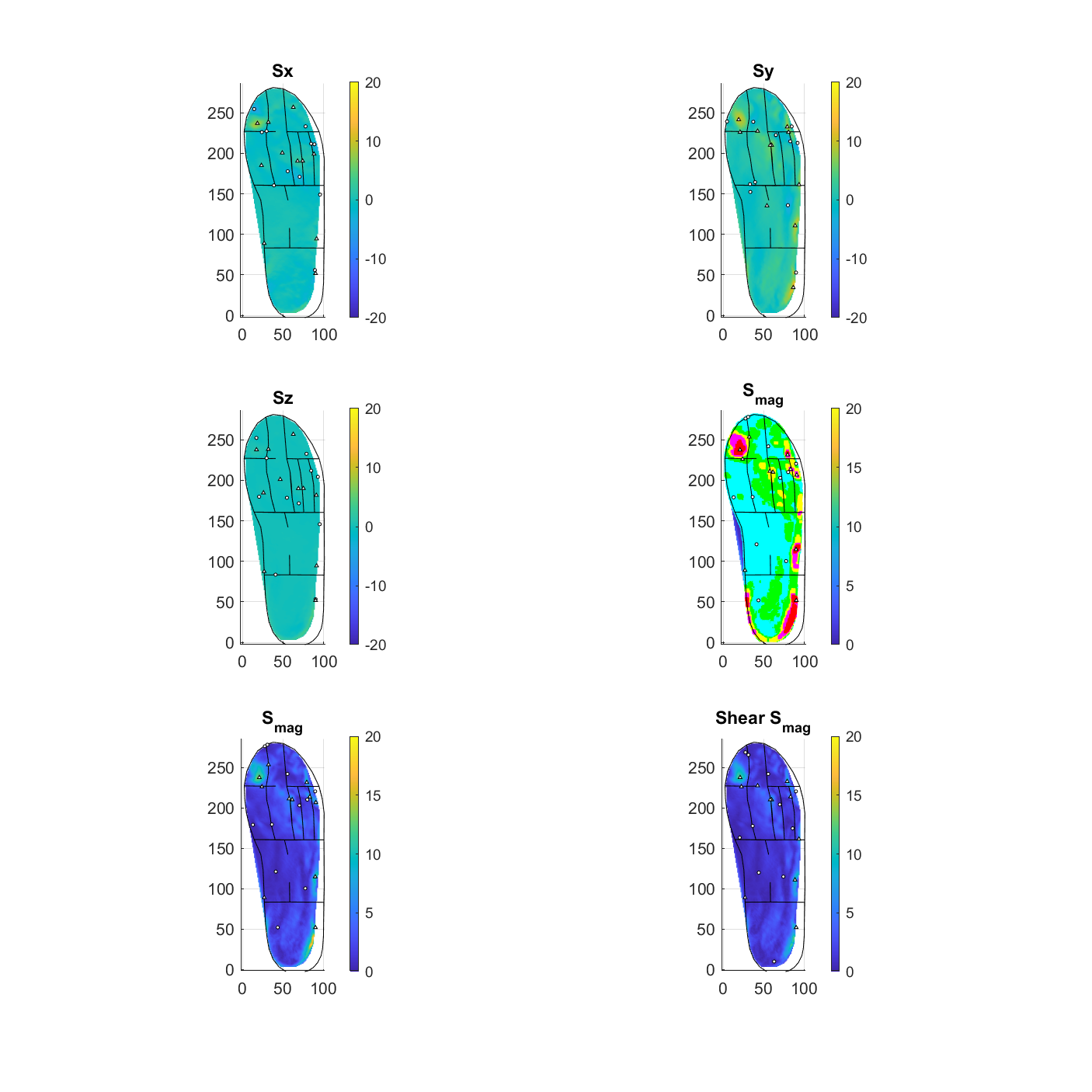 | | 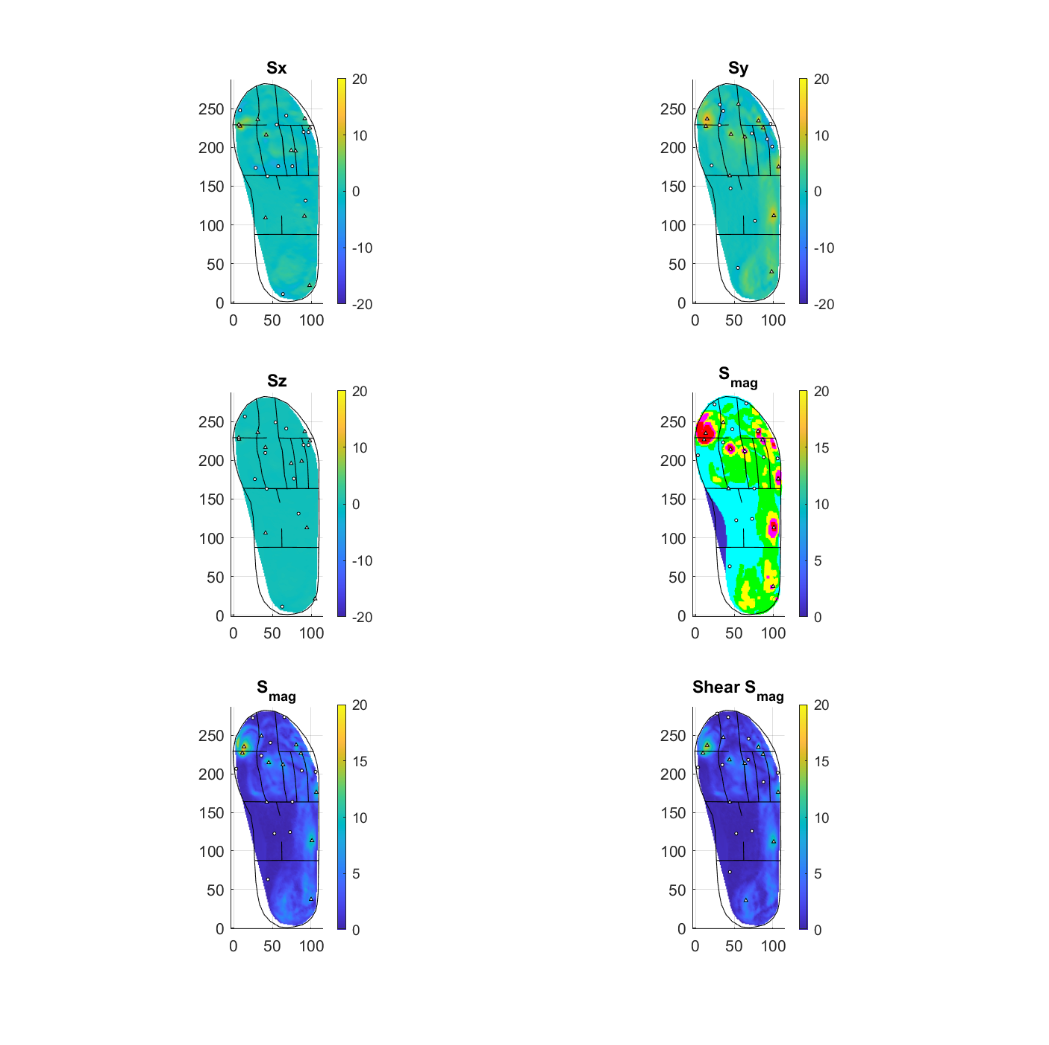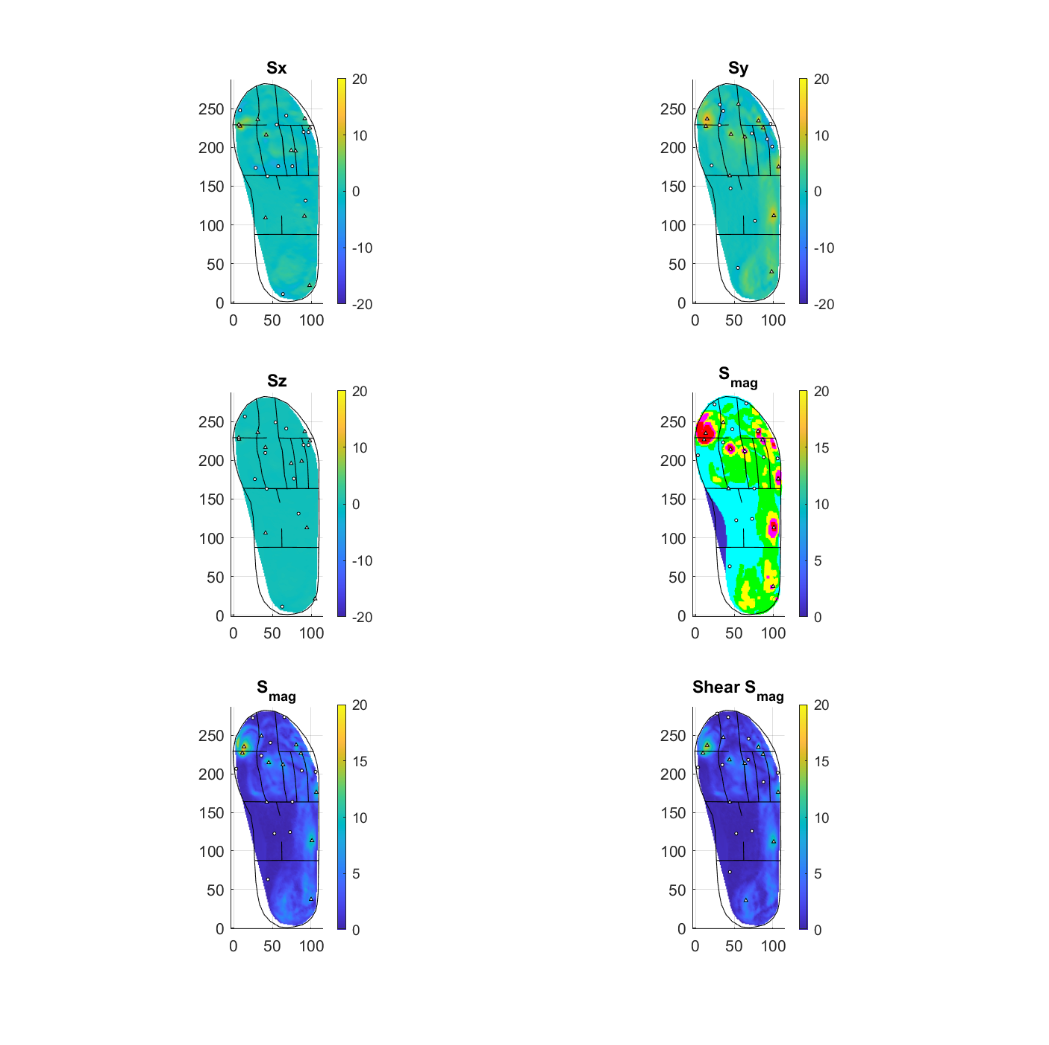 | |
| P04 | 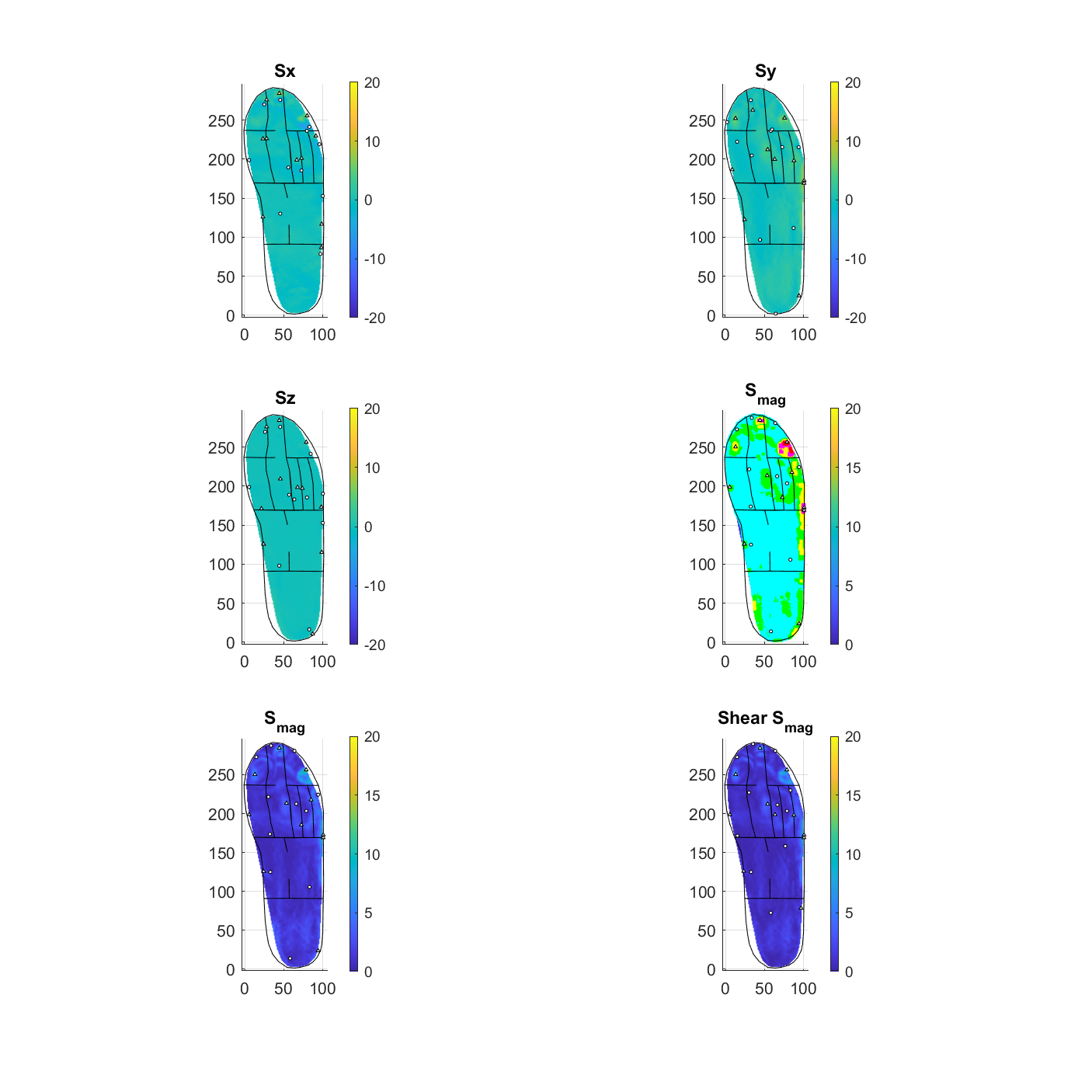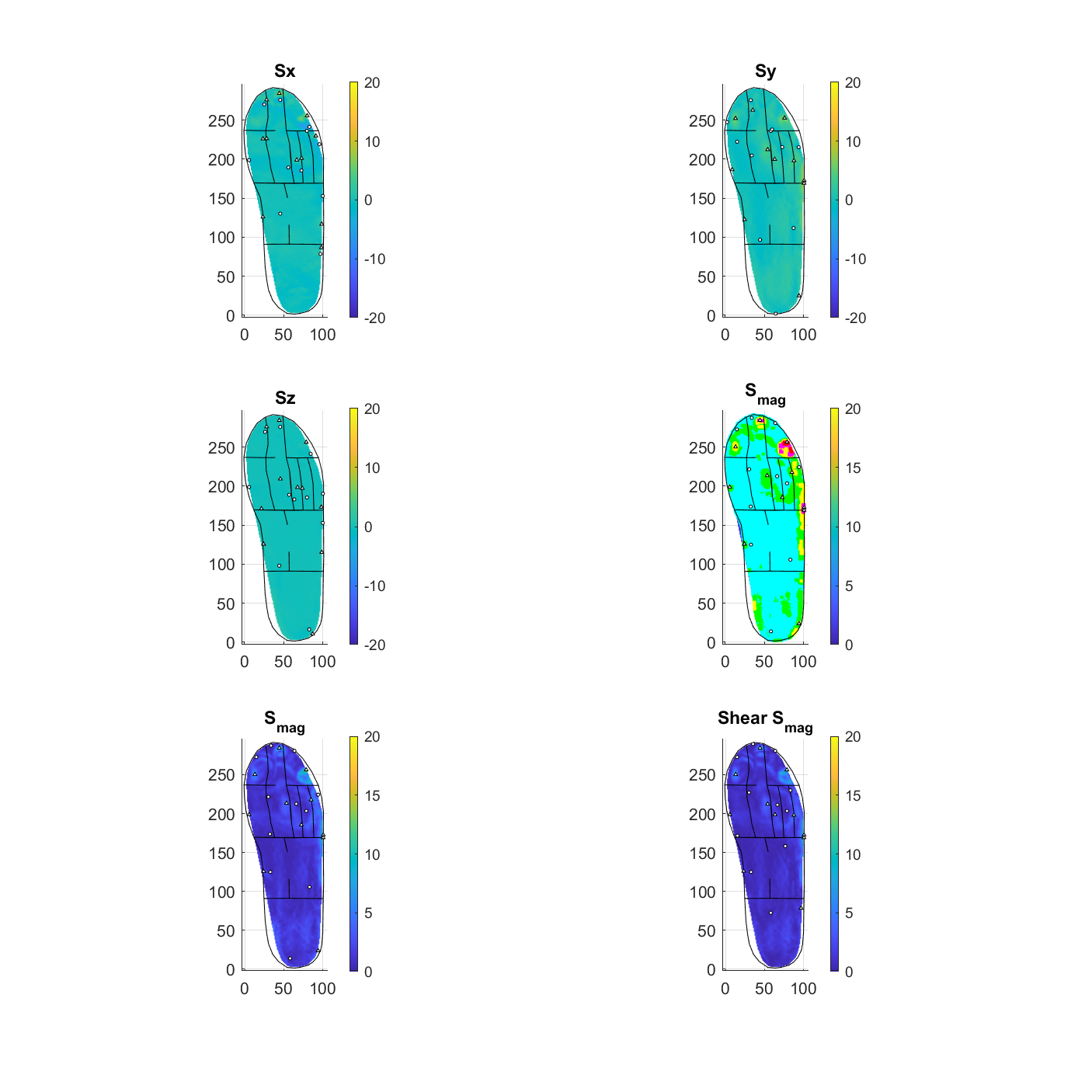 | | 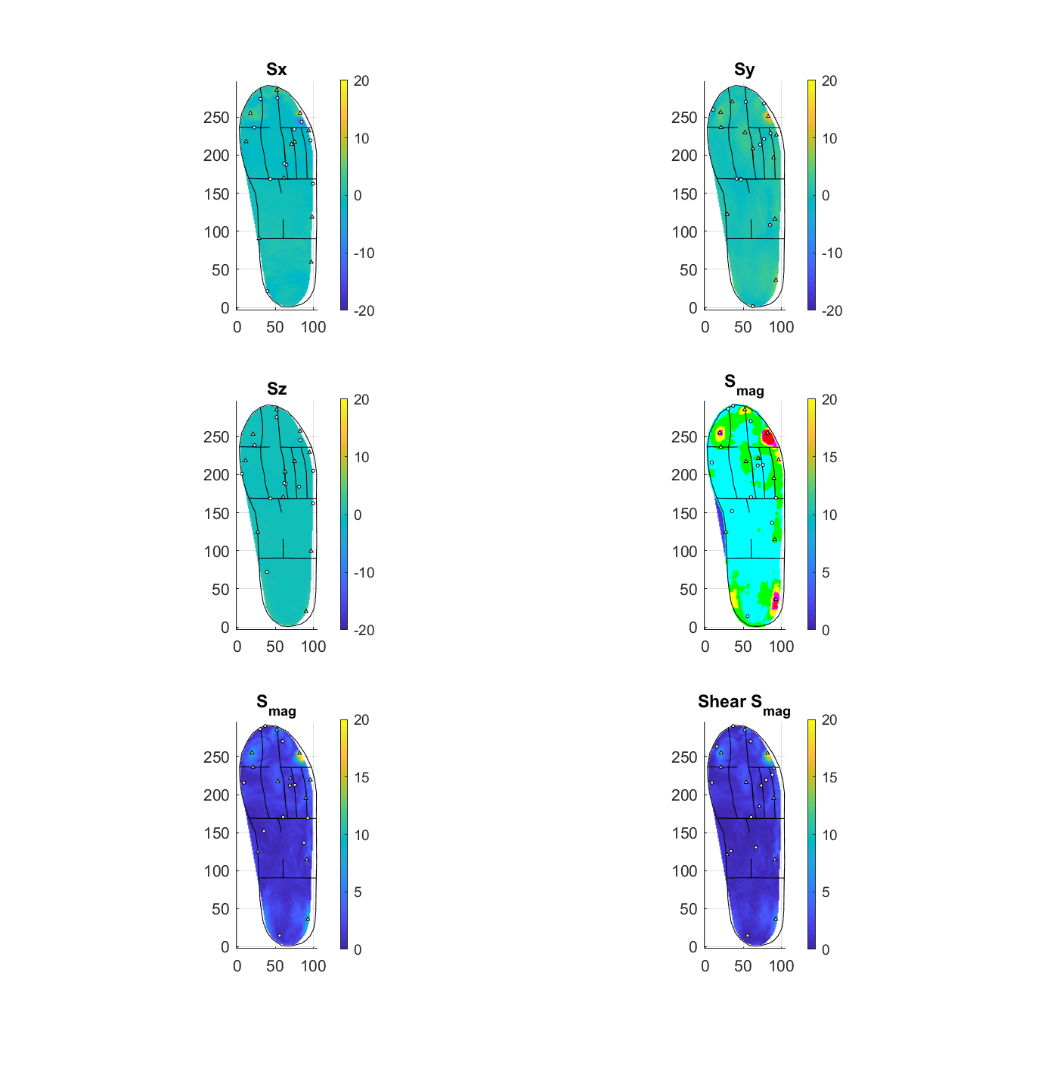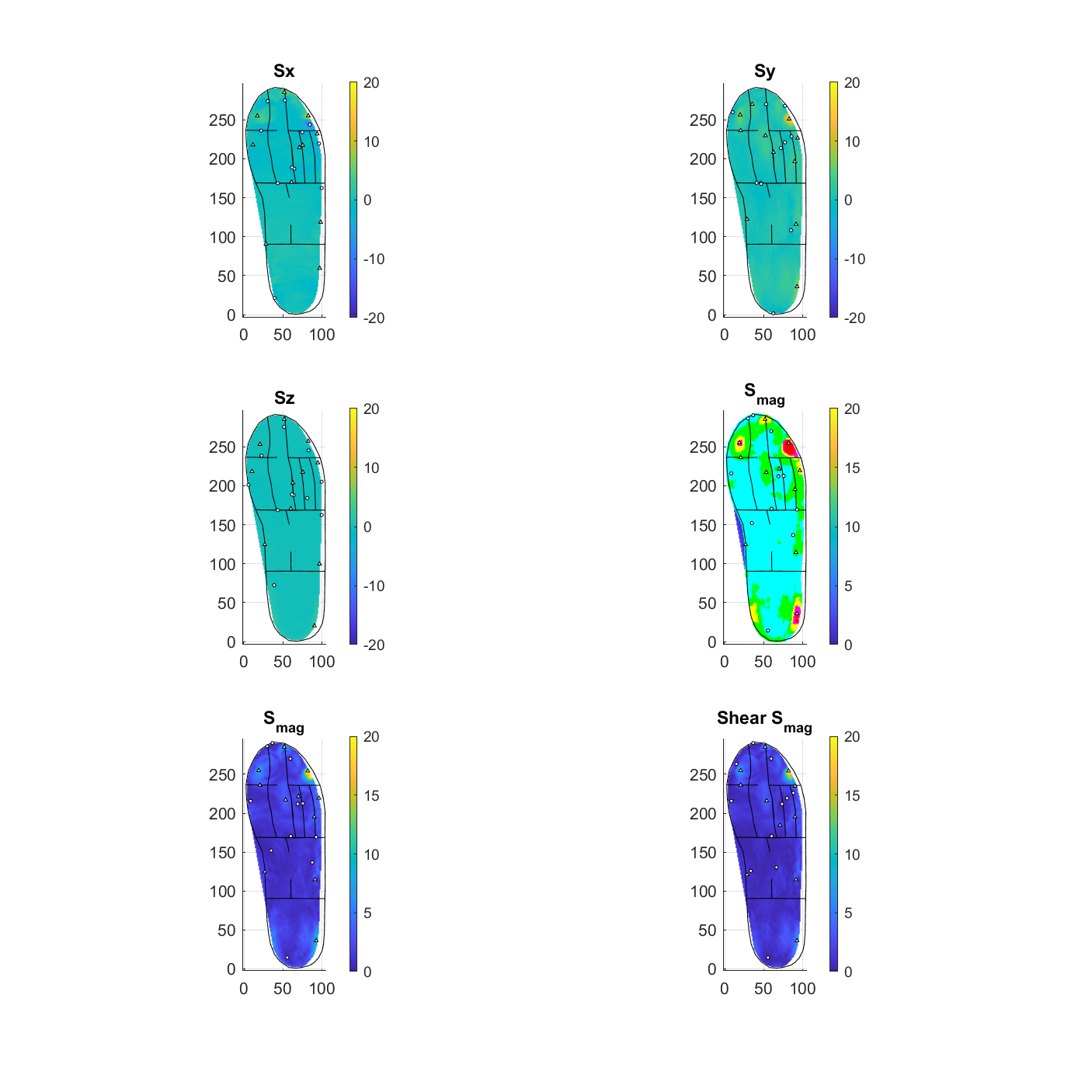 | | 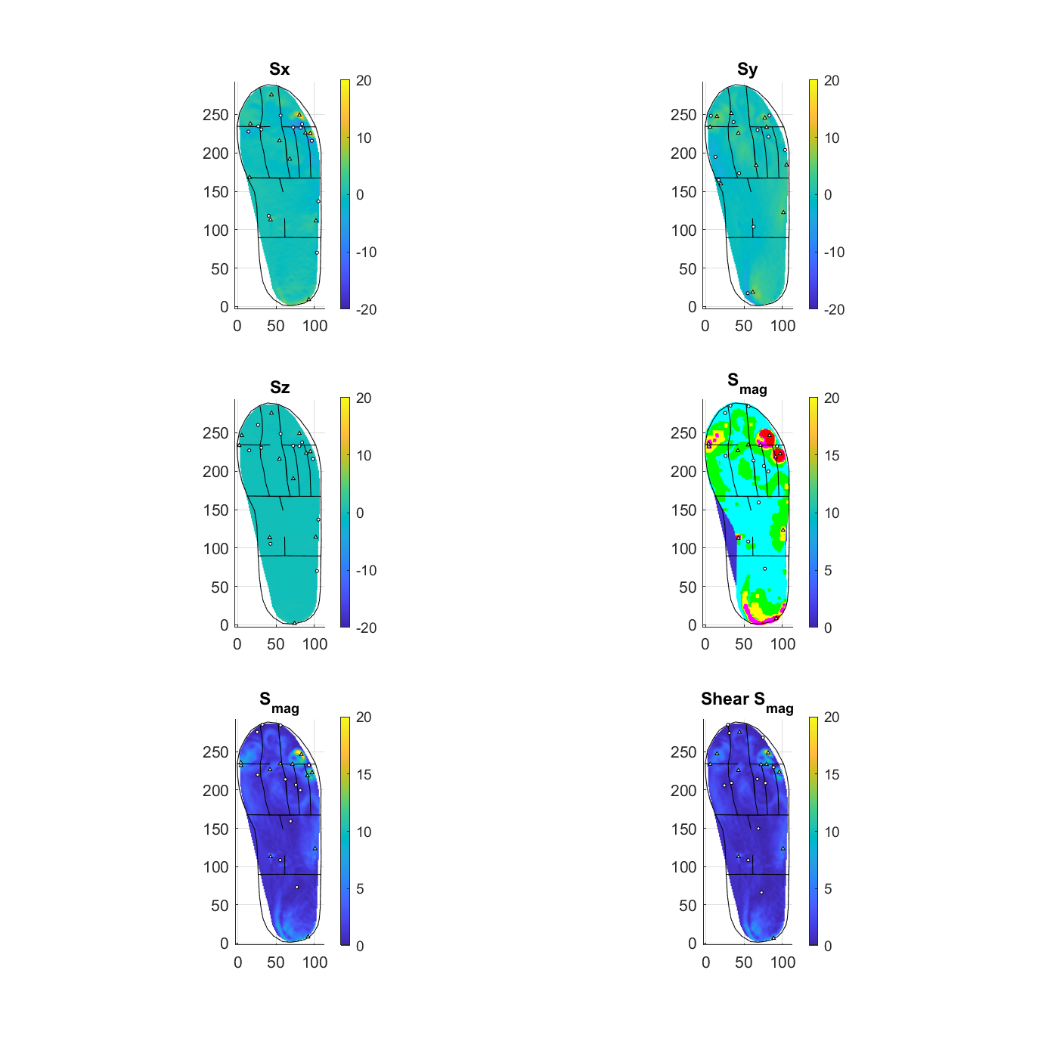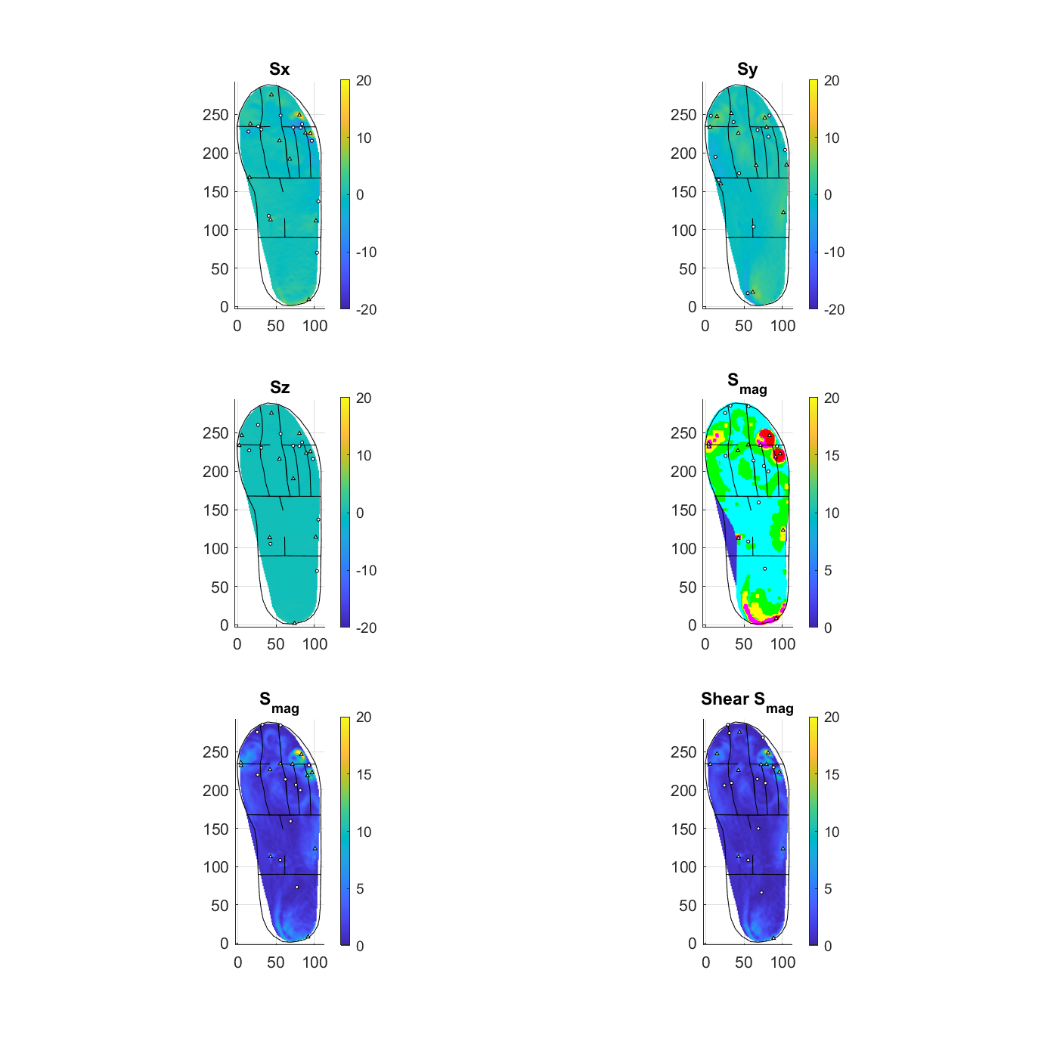 | |
| P05 | 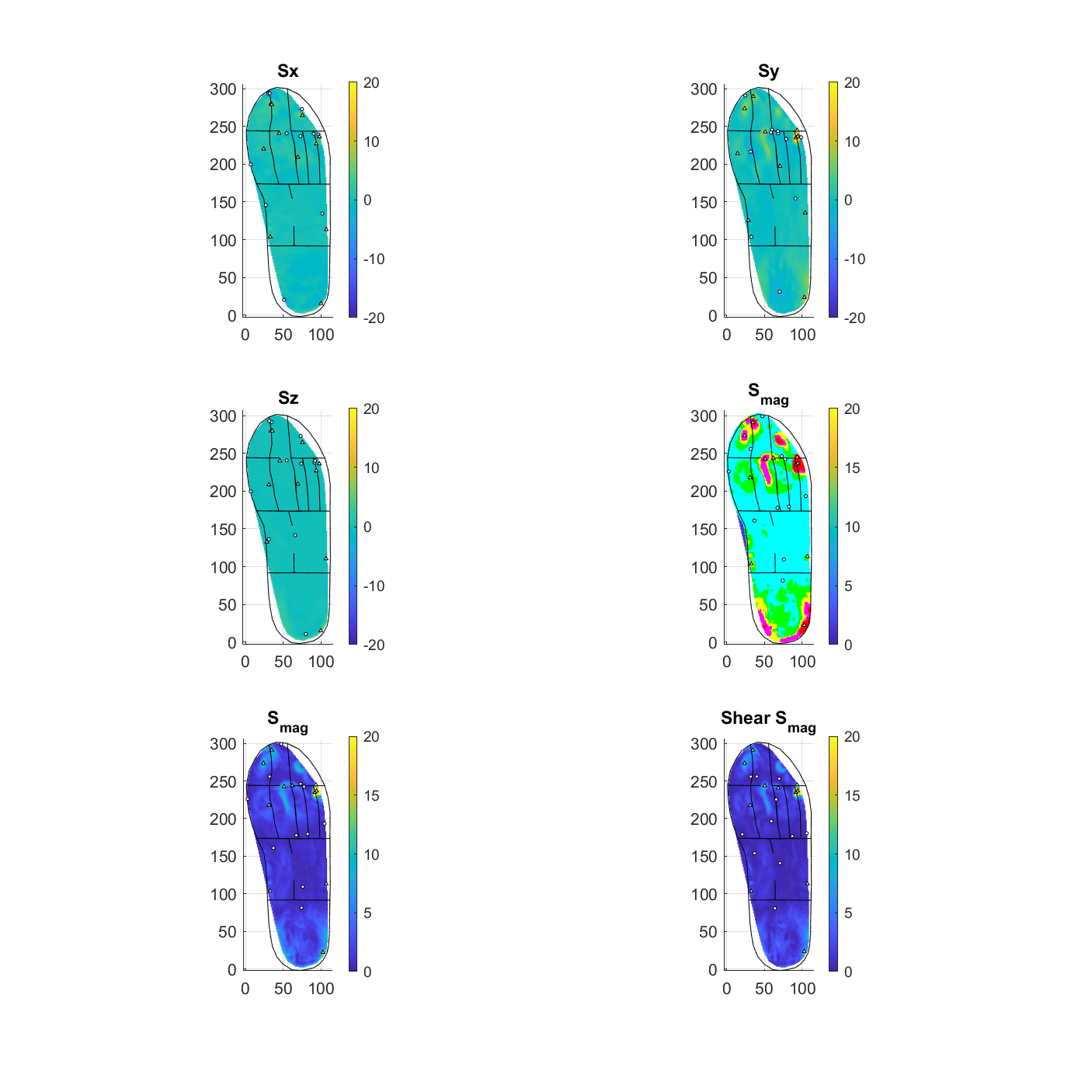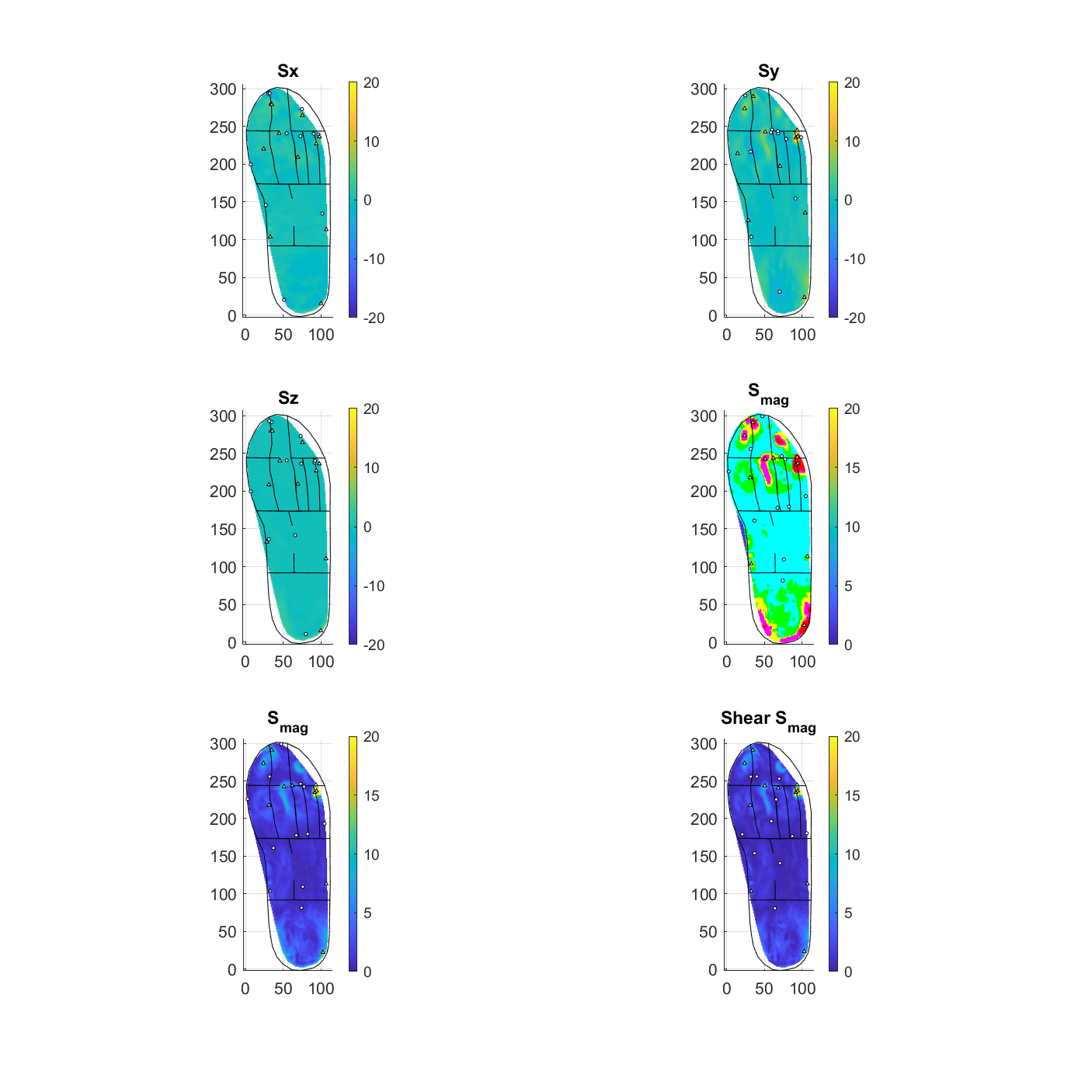 | | 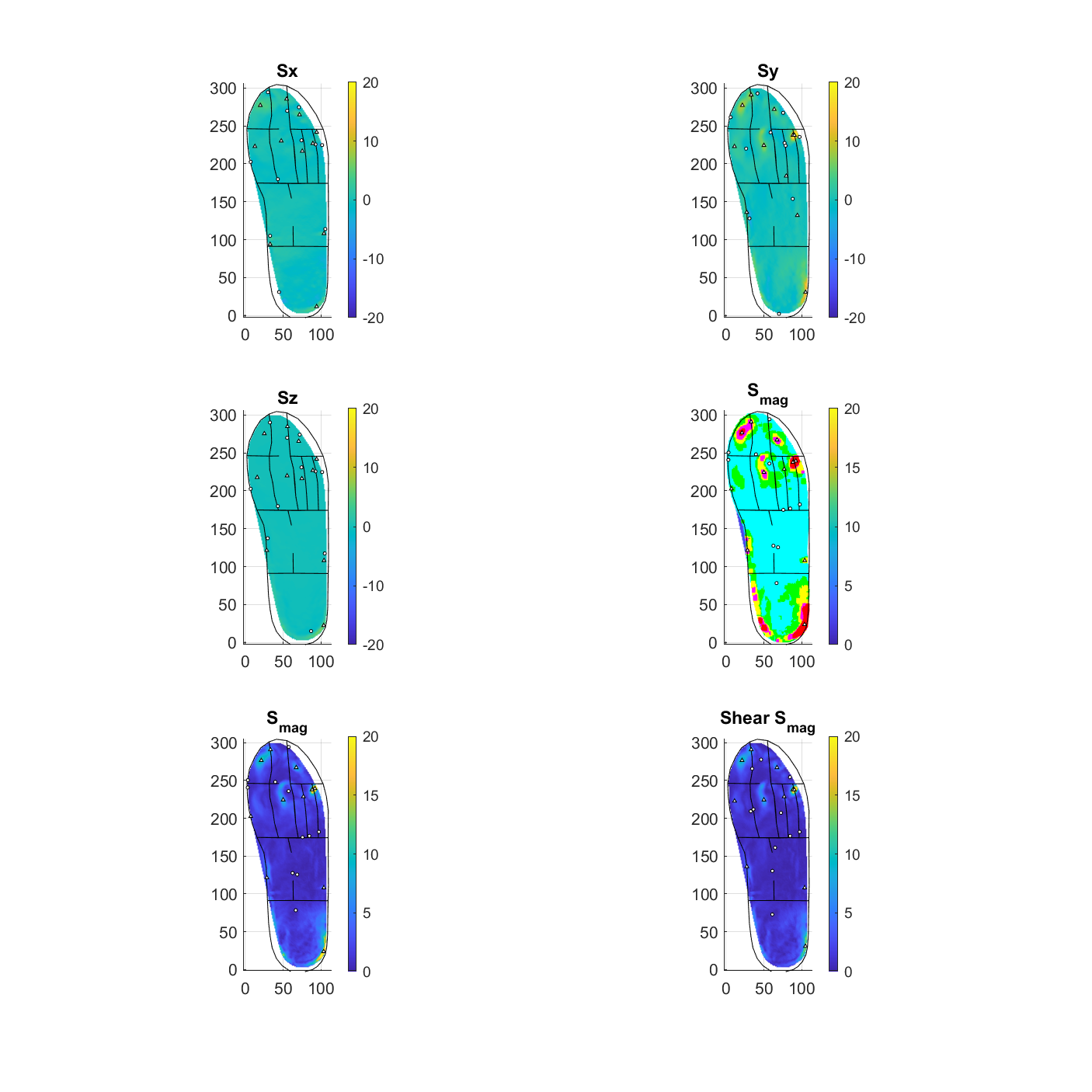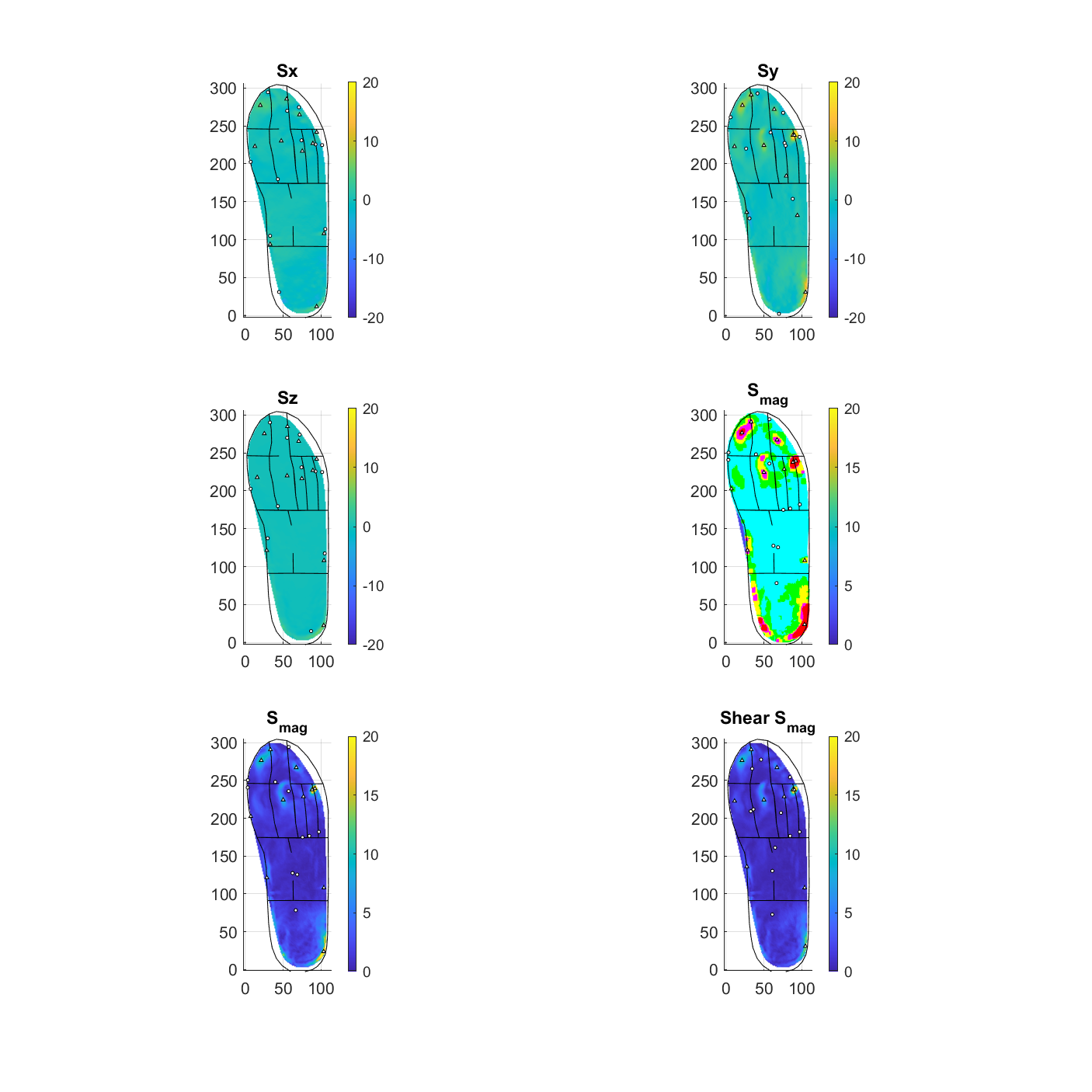 | | 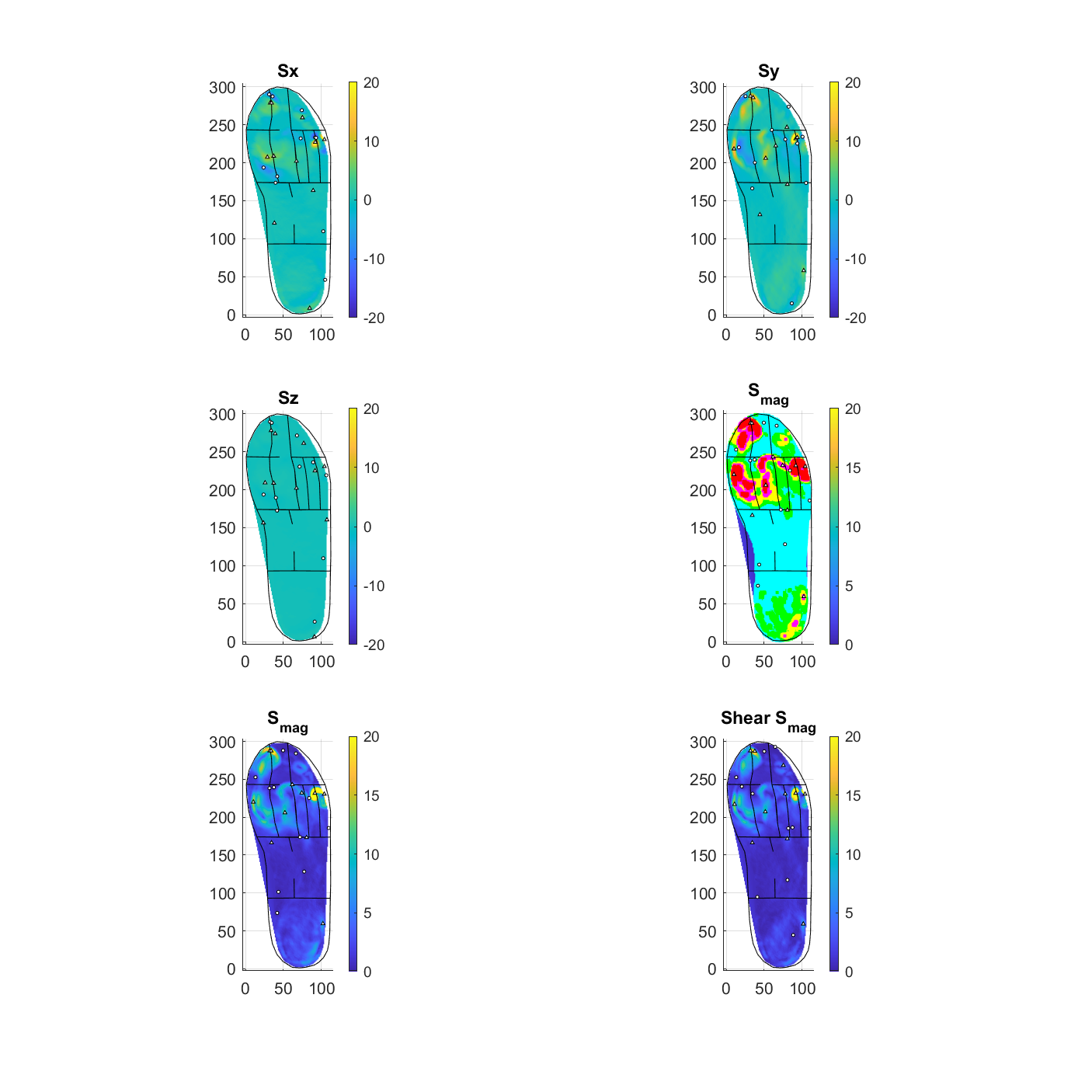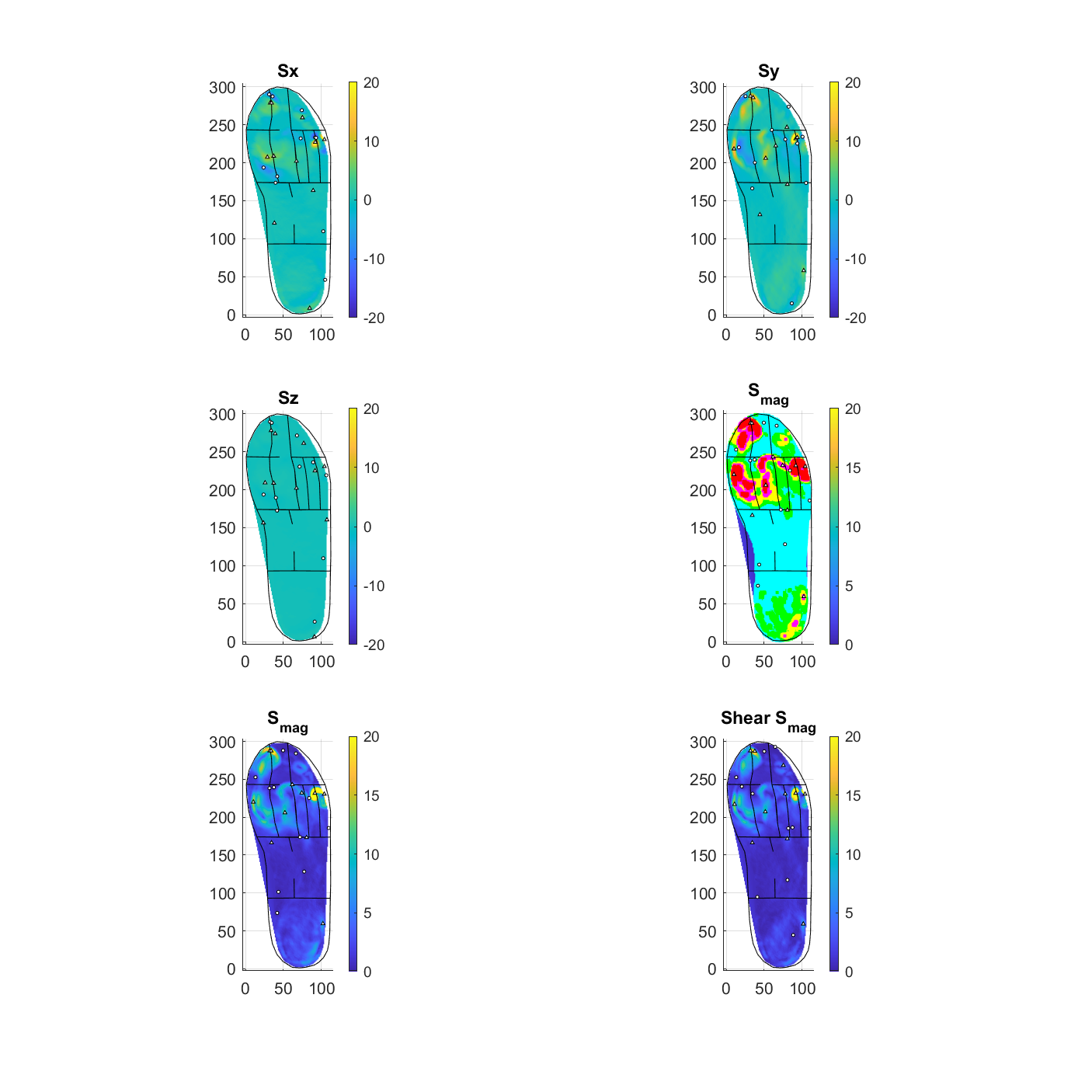 | |

**Supplementary Figure 1.** Representative strain maps for S_MAG_, trial 1 across each participant, with absolute plots demonstrating strain brackets: 0-2.5% (blue), 2.5-5% (green), 5-7.5% (yellow), 7.5-10% (magenta) and > 10 (red).

|  | **Low Stiffness** | | **High Stiffness** | | **STAMPS** | |
| --- | --- | --- | --- | --- | --- | --- |
|  | **Strain Map (S_MAG_)** | **Absolute Strain Plot** | **Strain Map (S_MAG_)** | **Absolute Strain Plot** | **Strain Map (S_MAG_)** | **Absolute Strain Plot** |
| P01 | 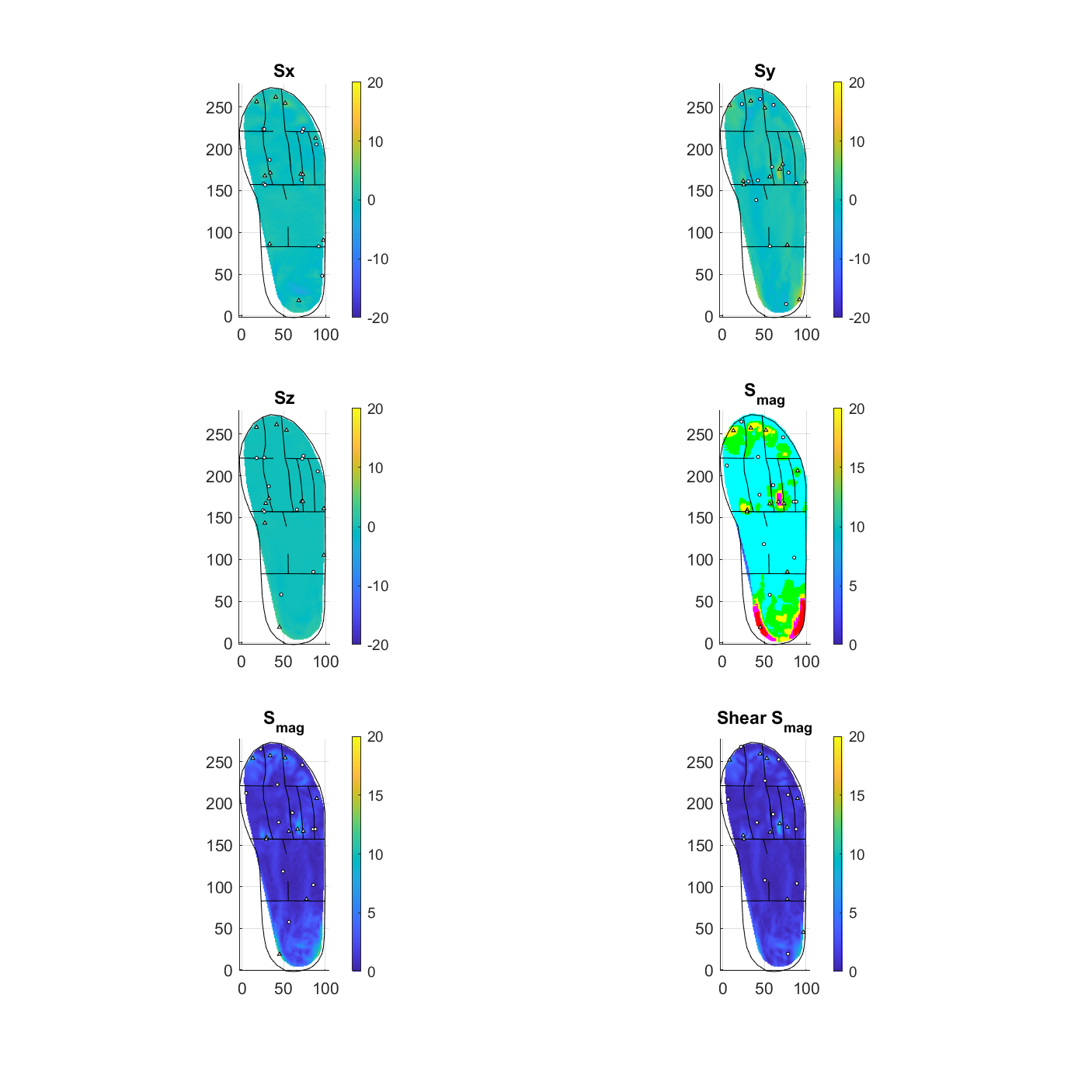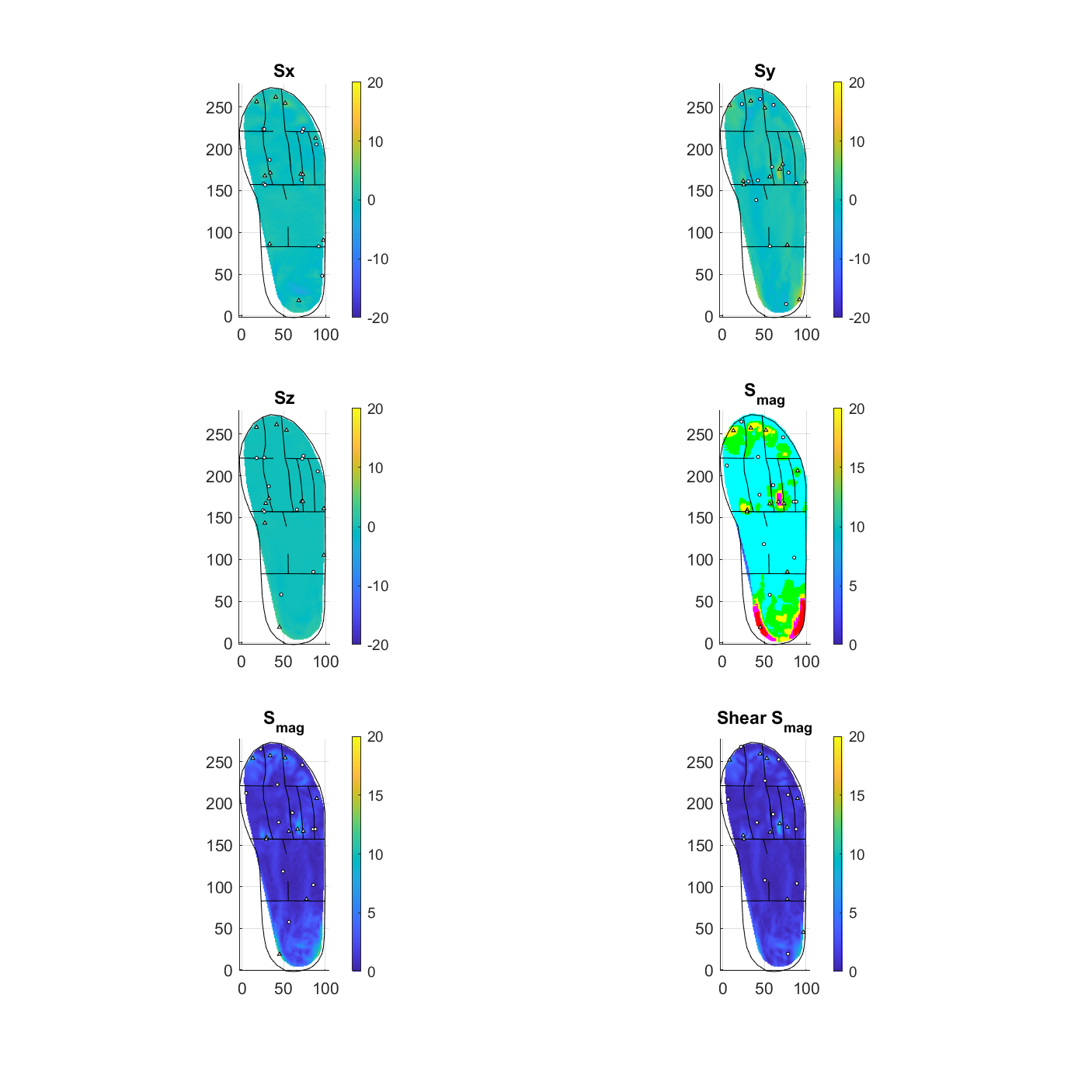 | | 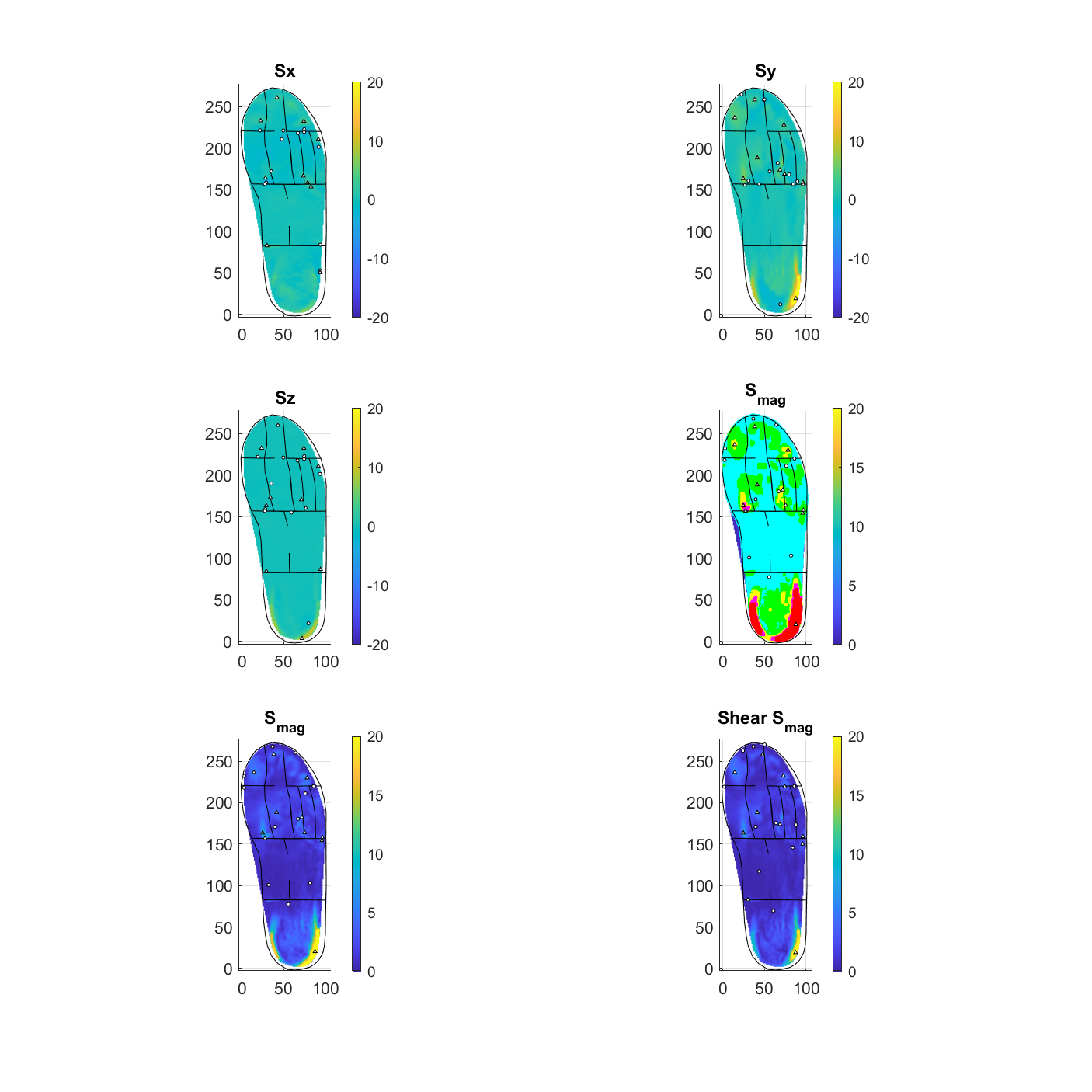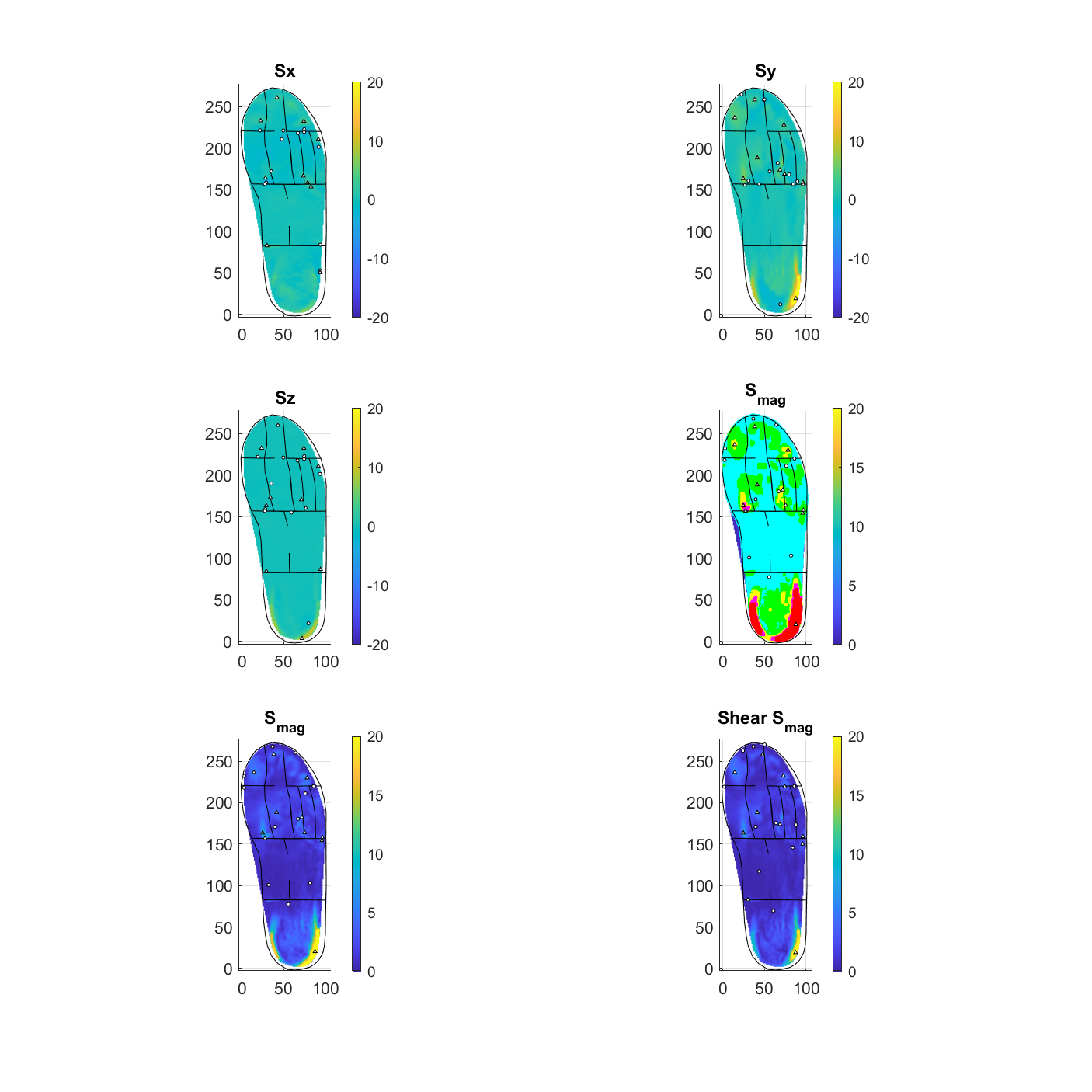 | | 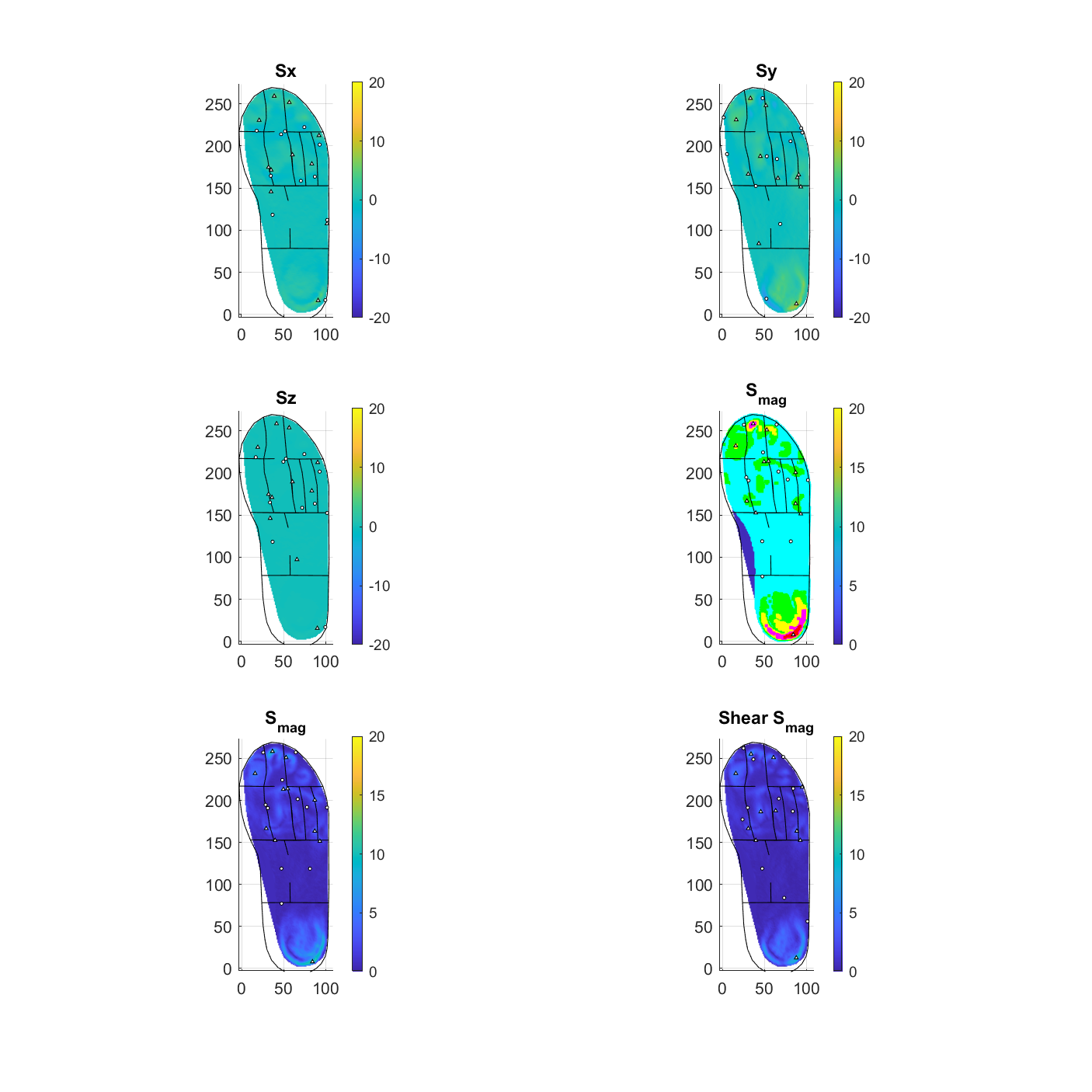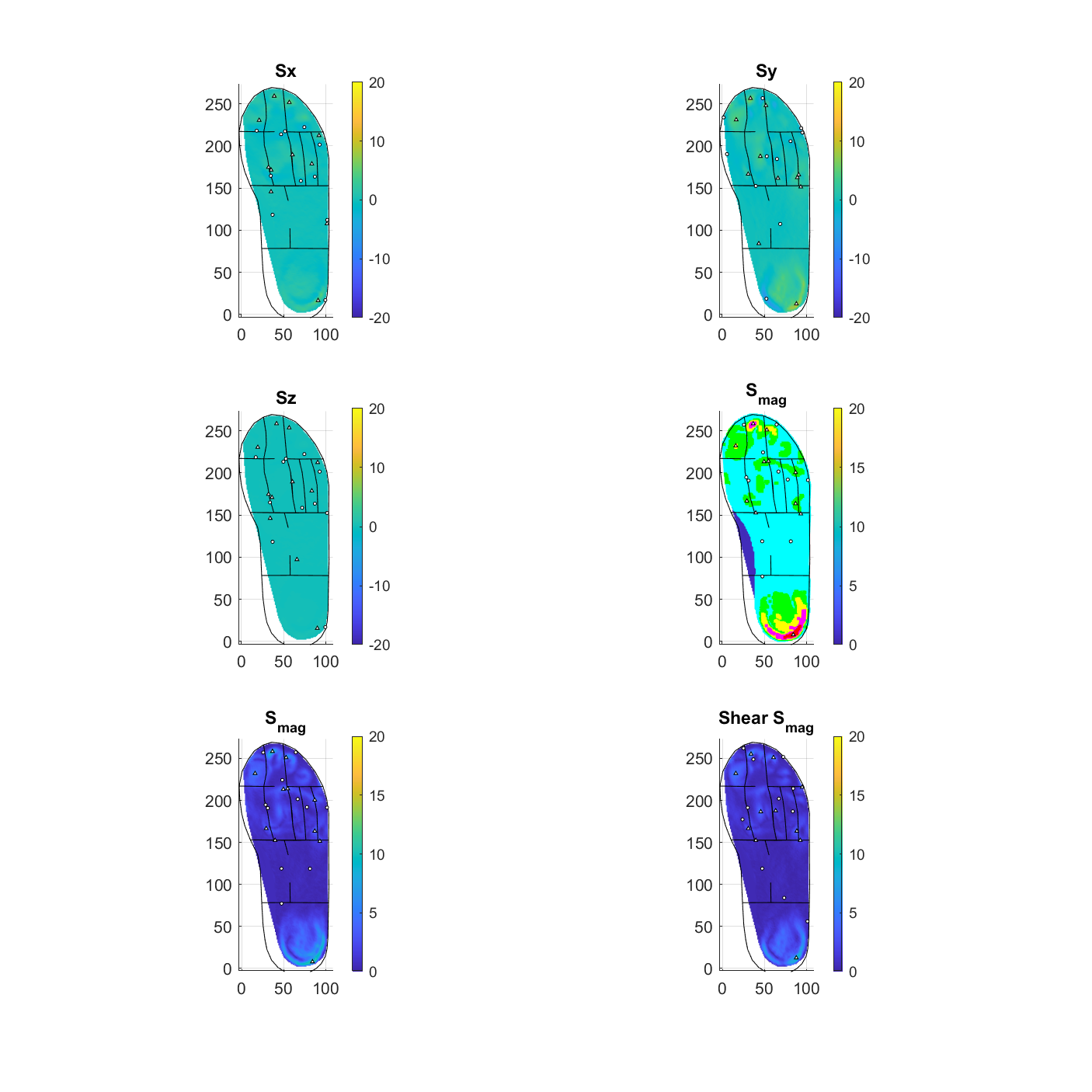 | |
| P02 | 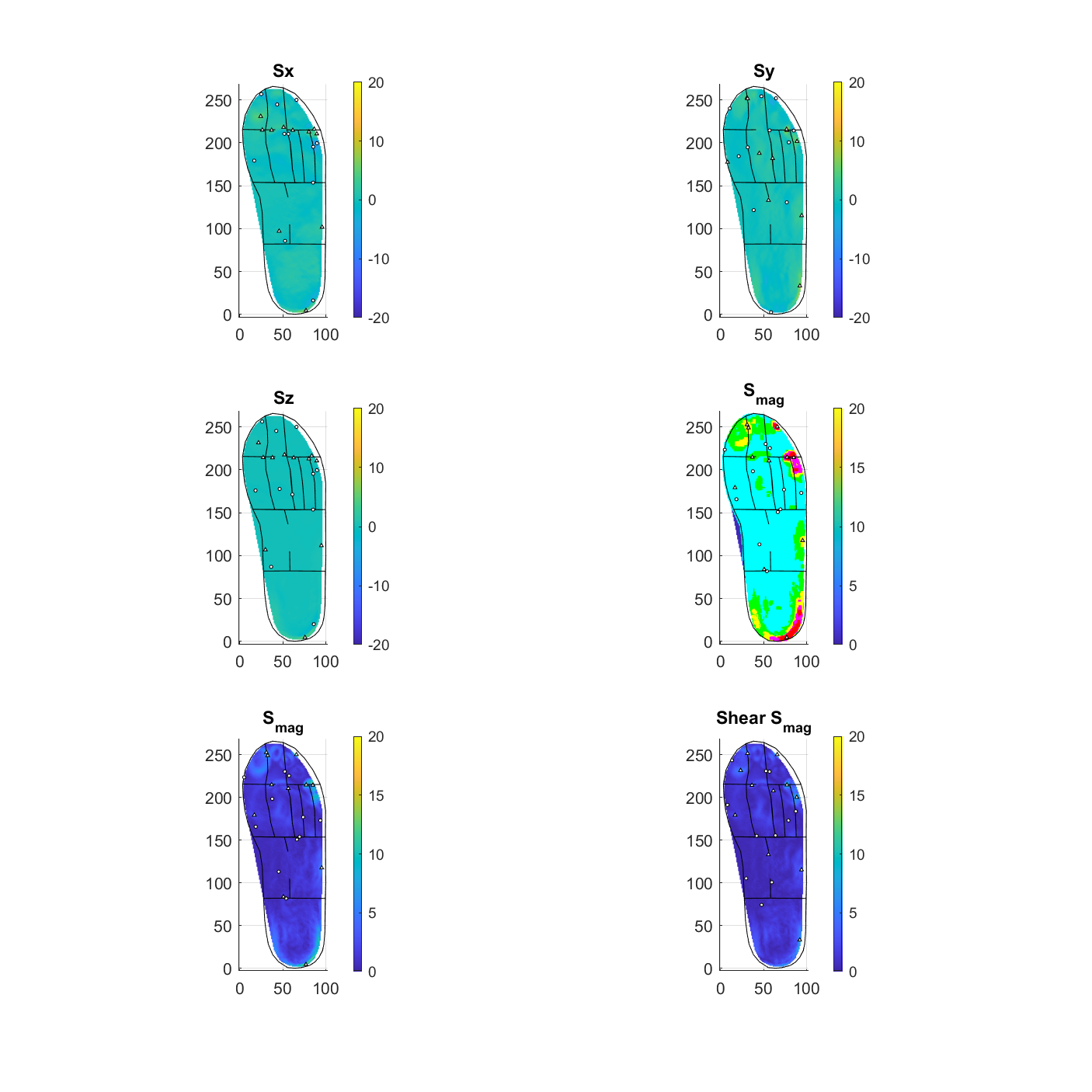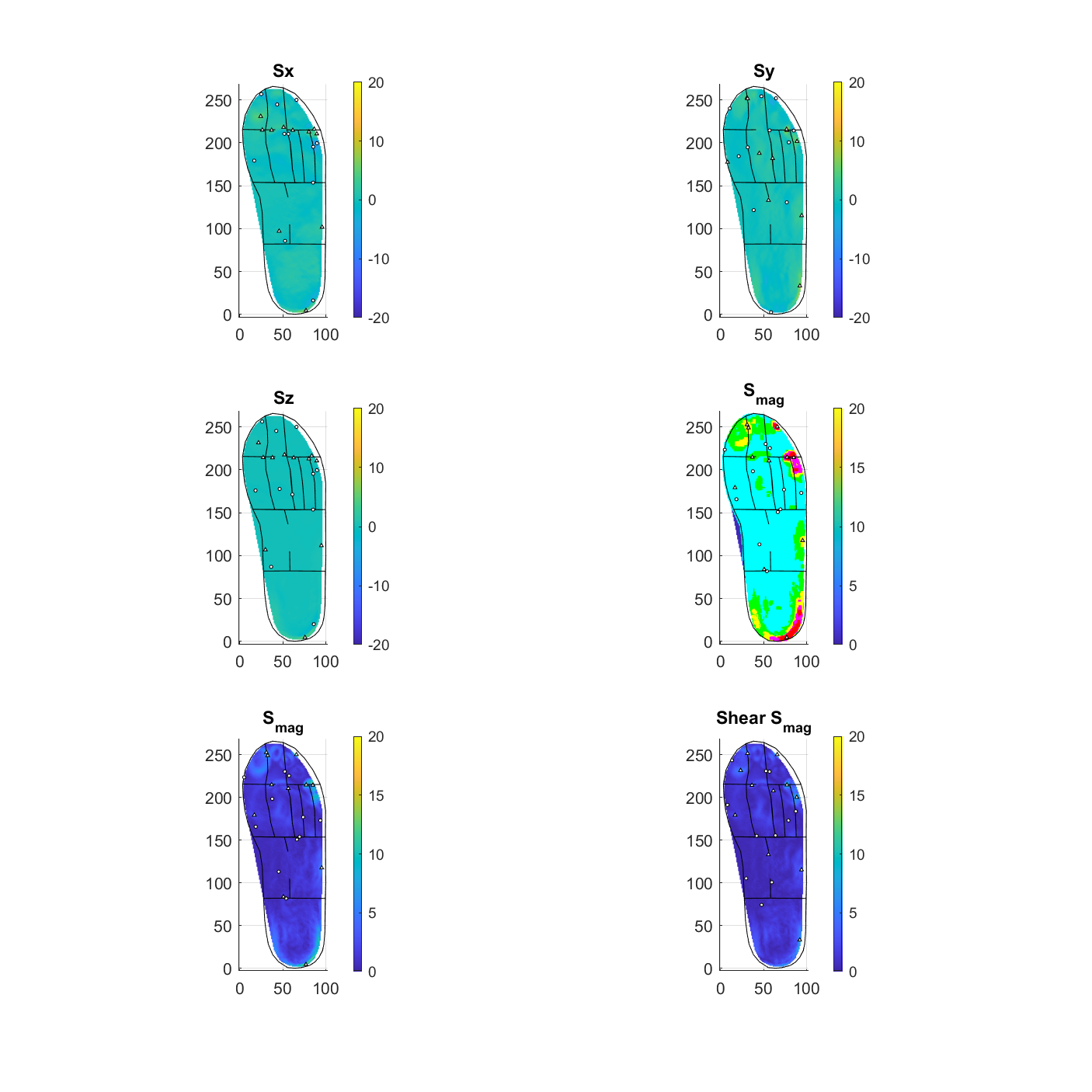 | | 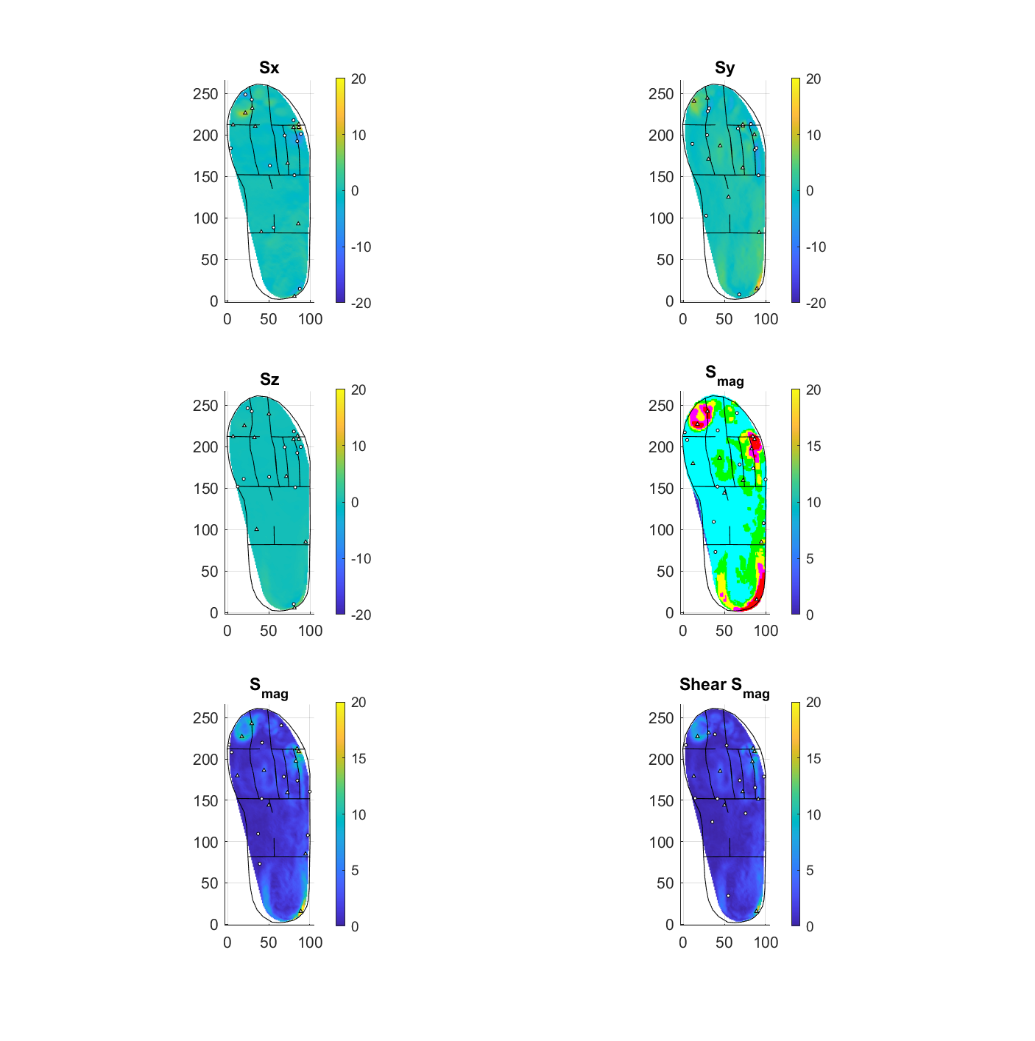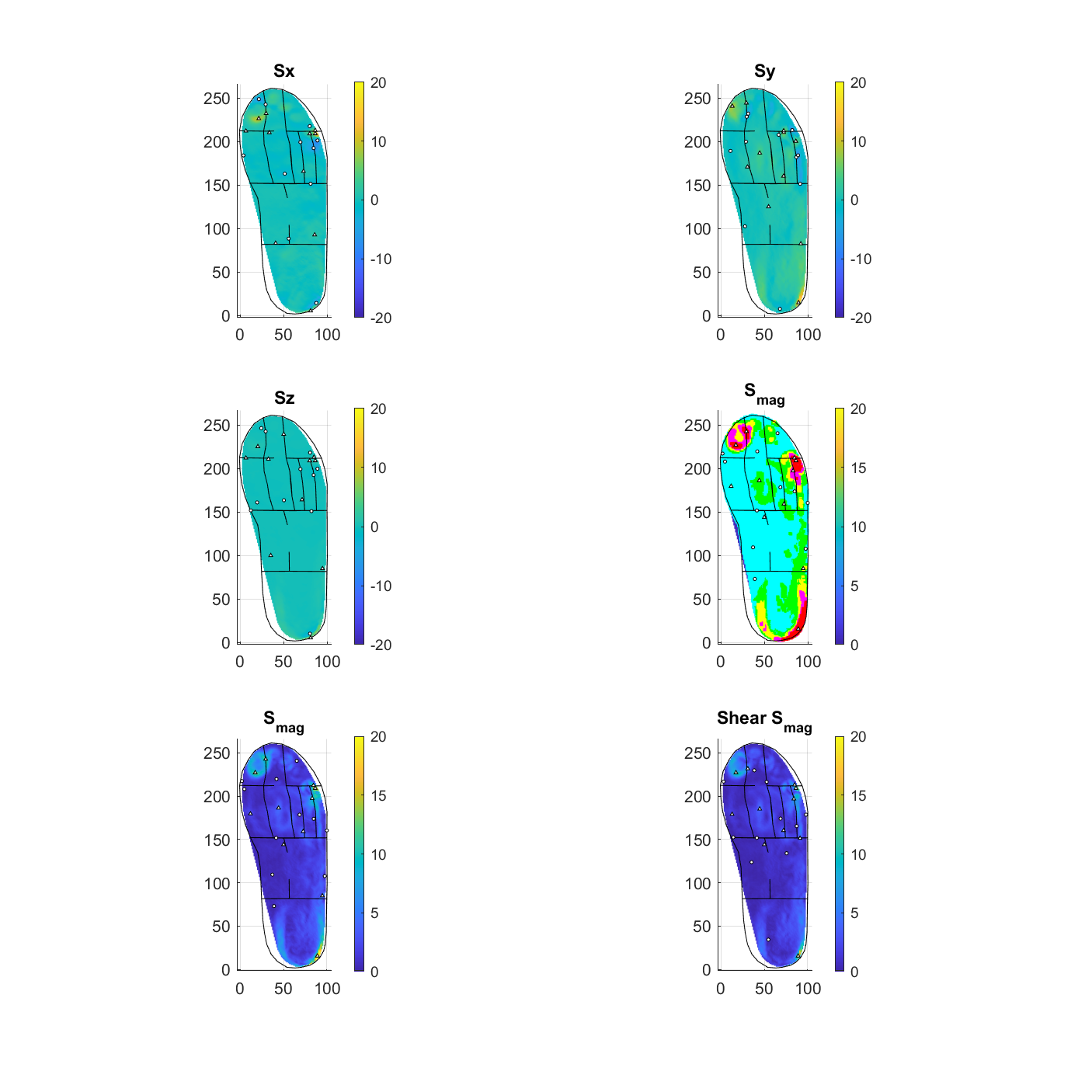 | | 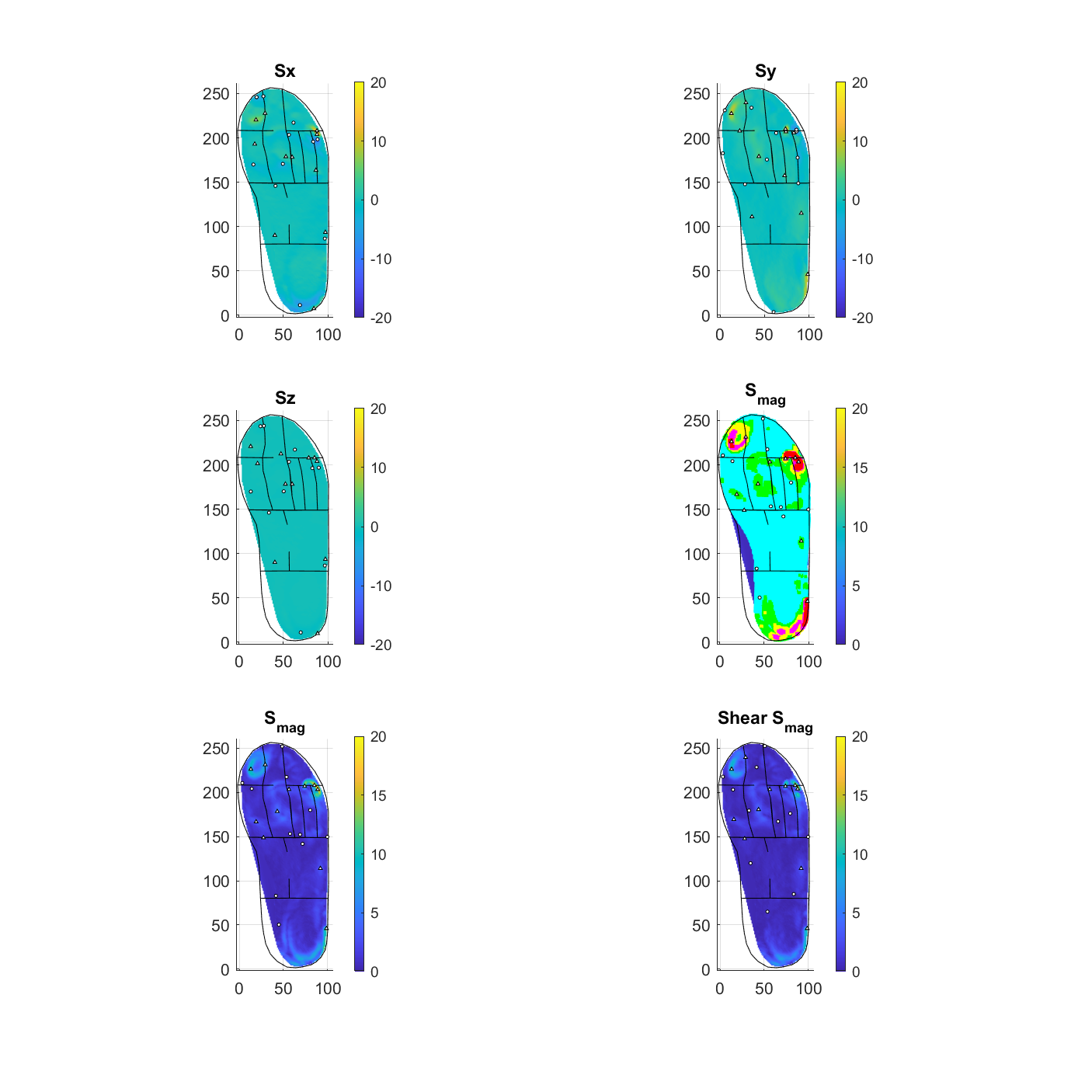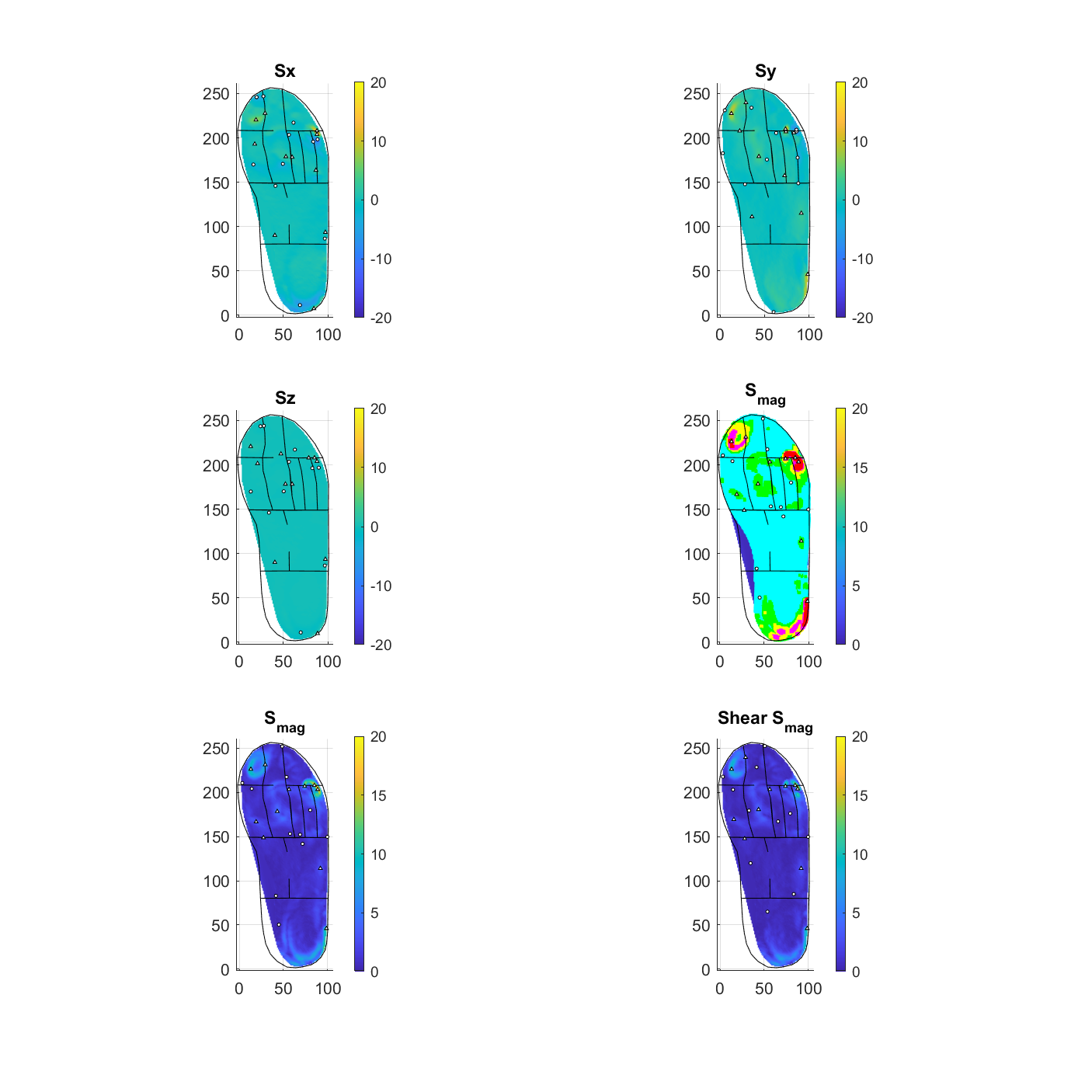 | |
| P03 | 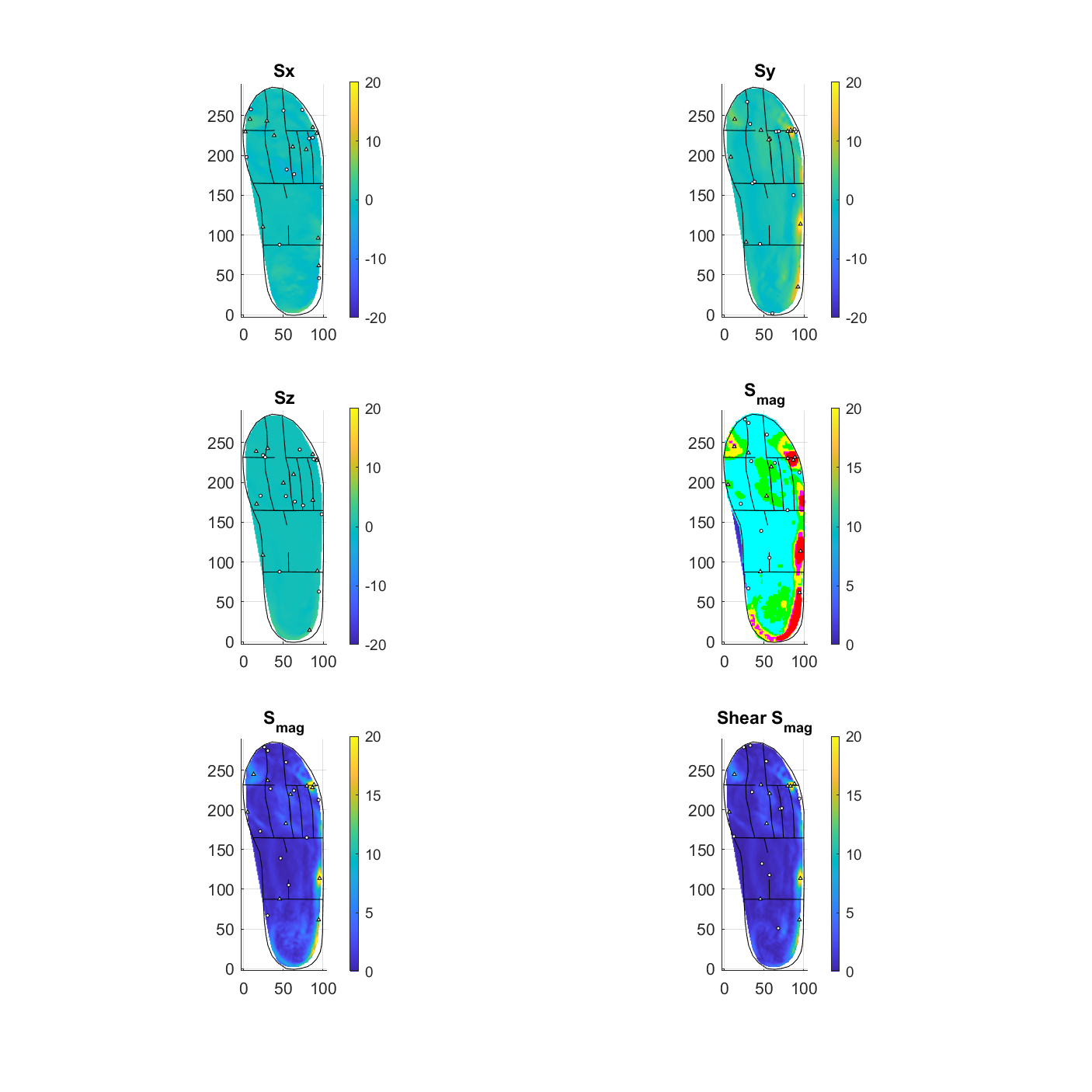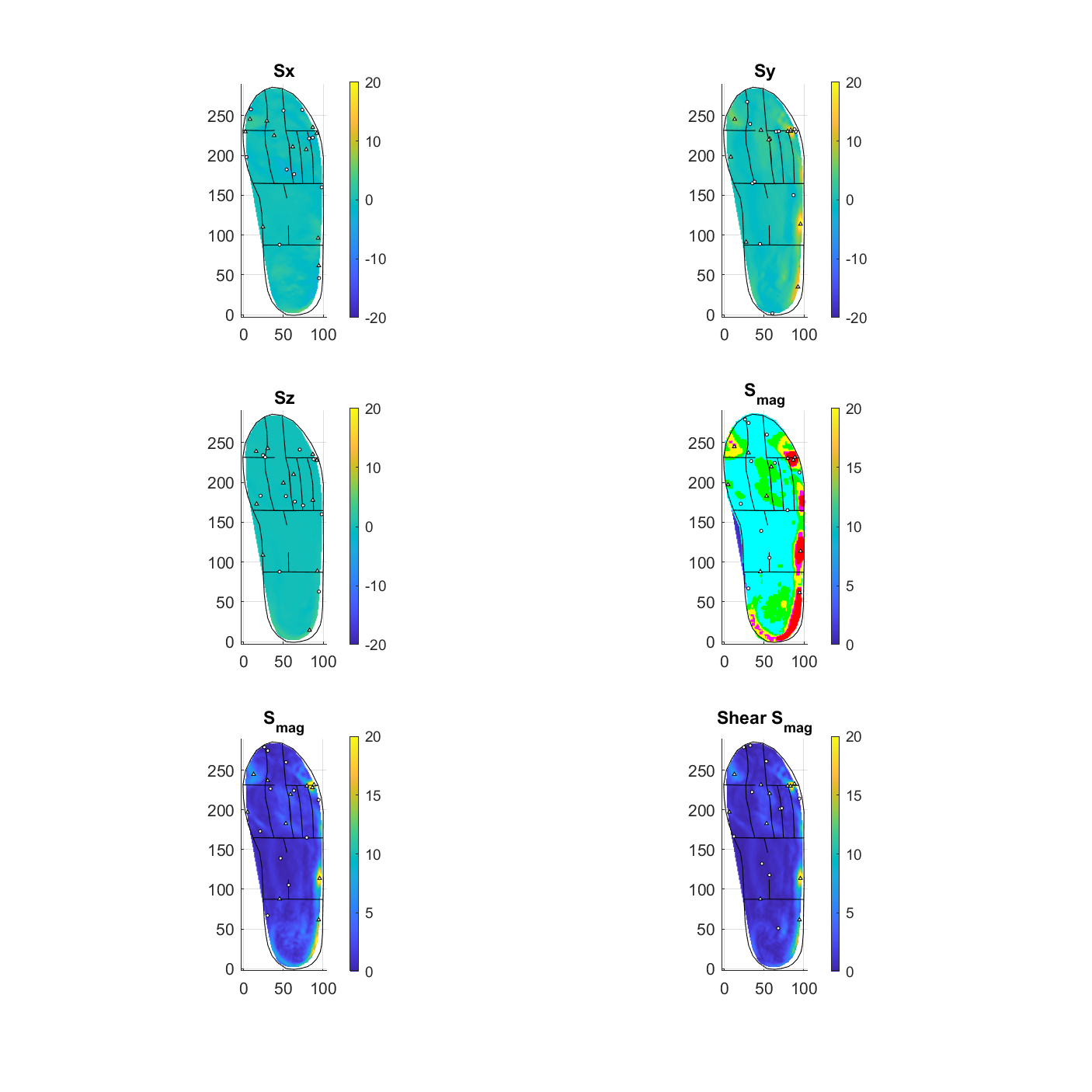 | | 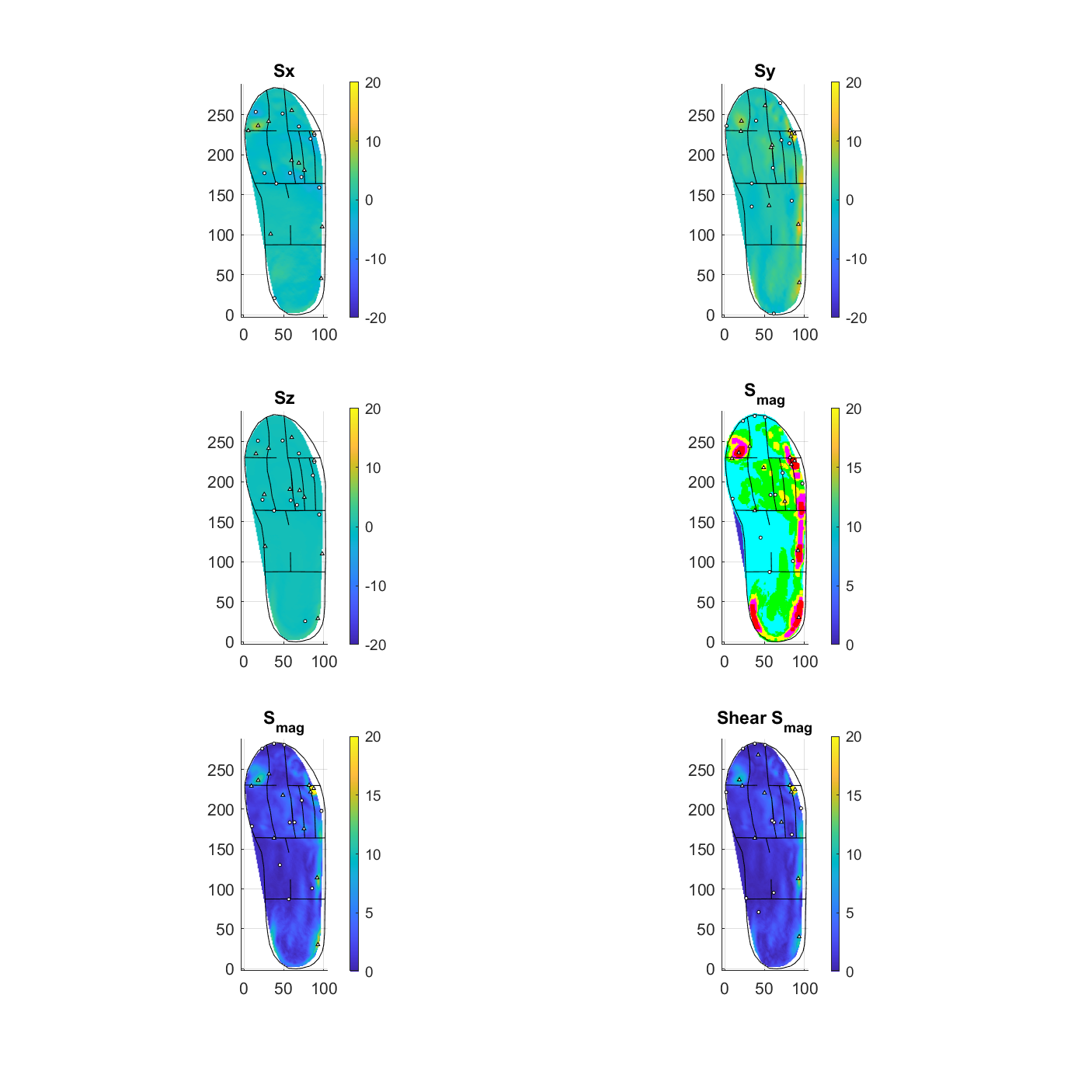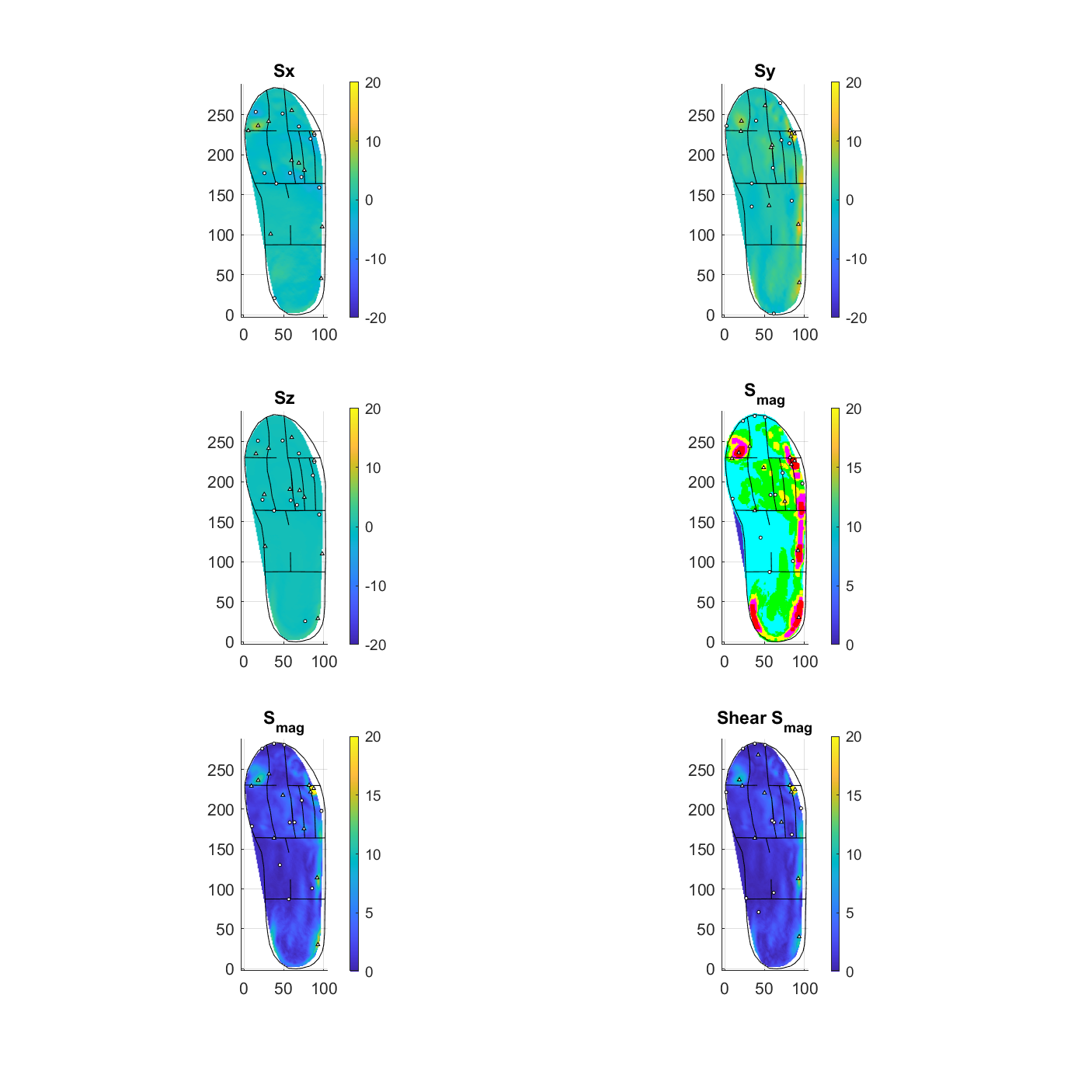 | | 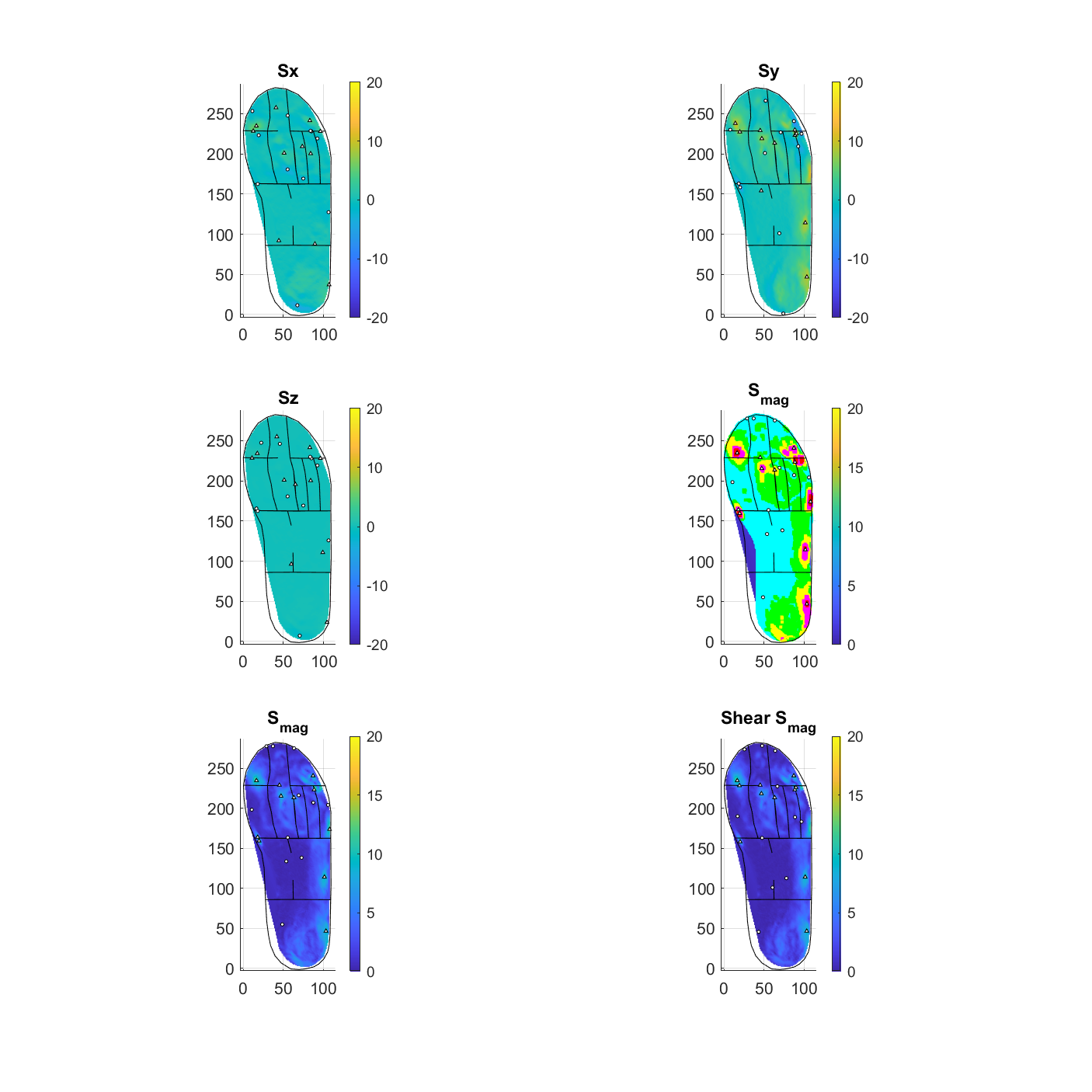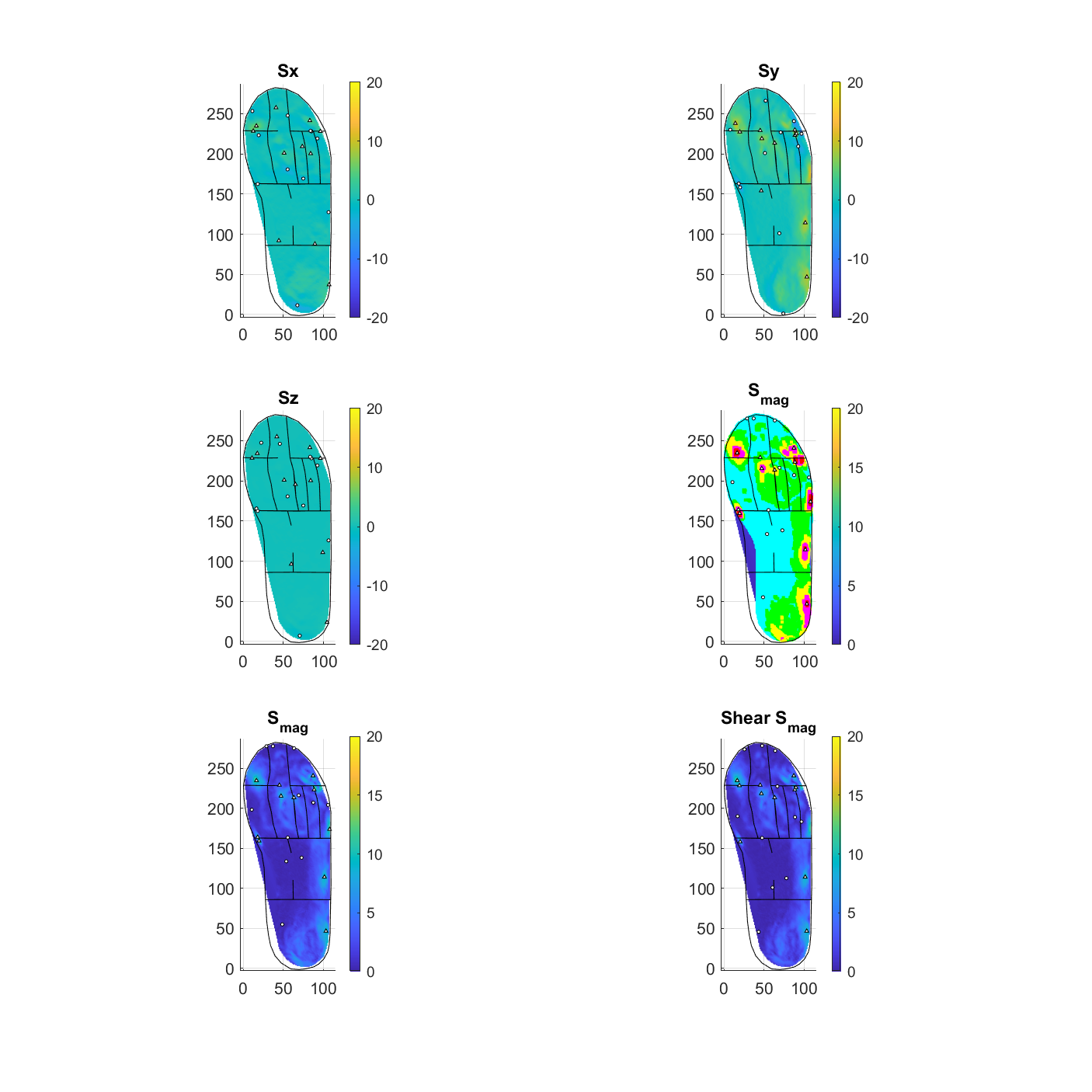 | |
| P04 | 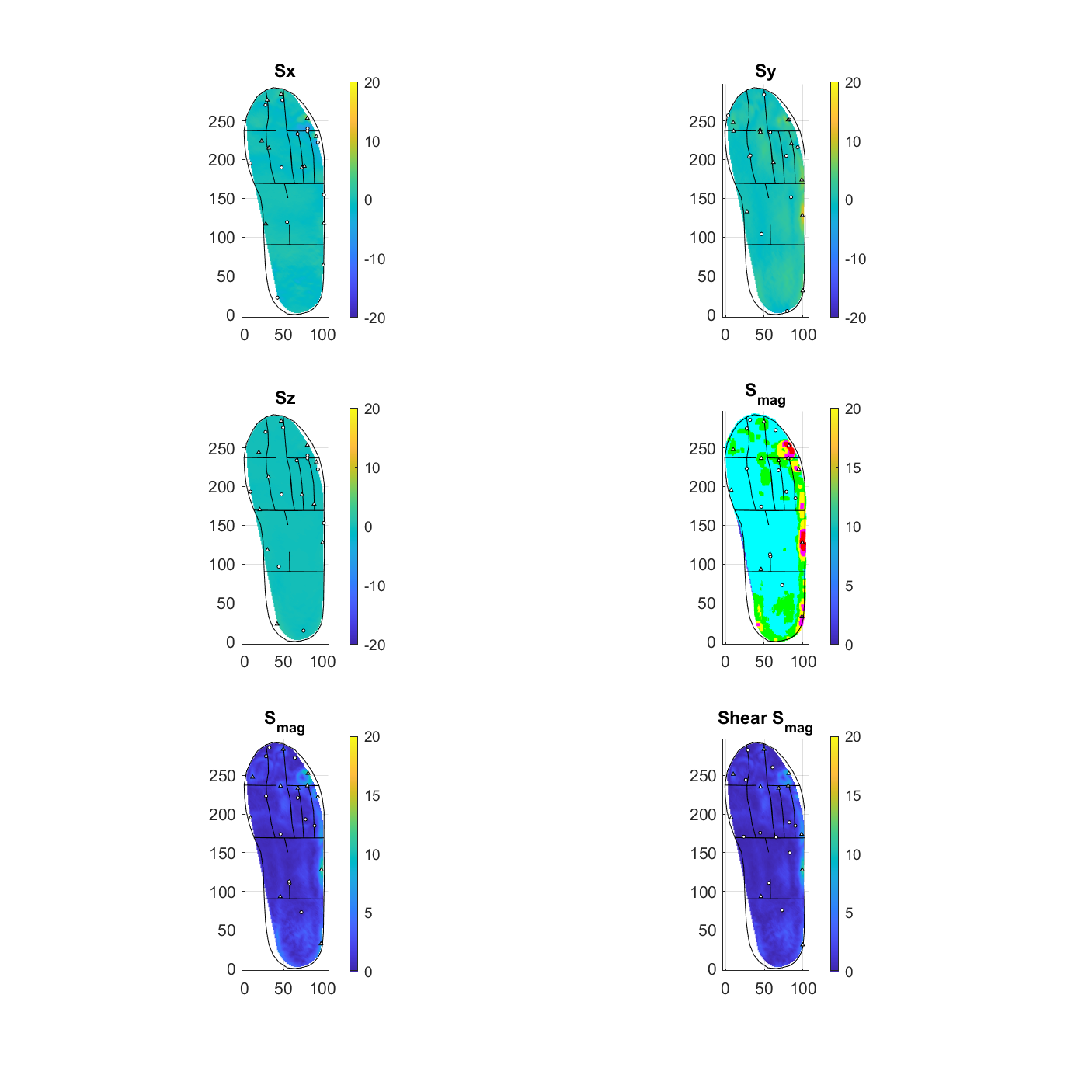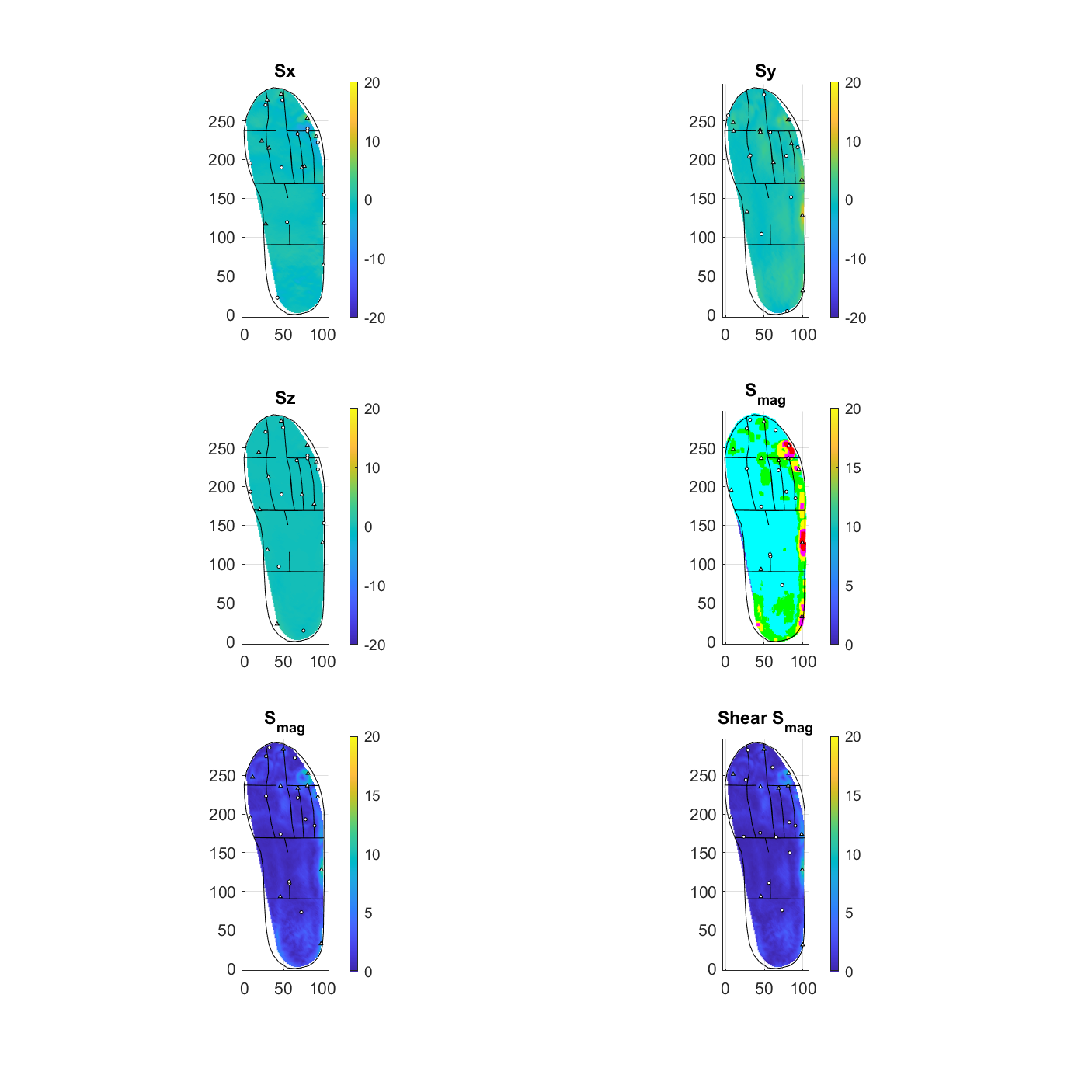 | | 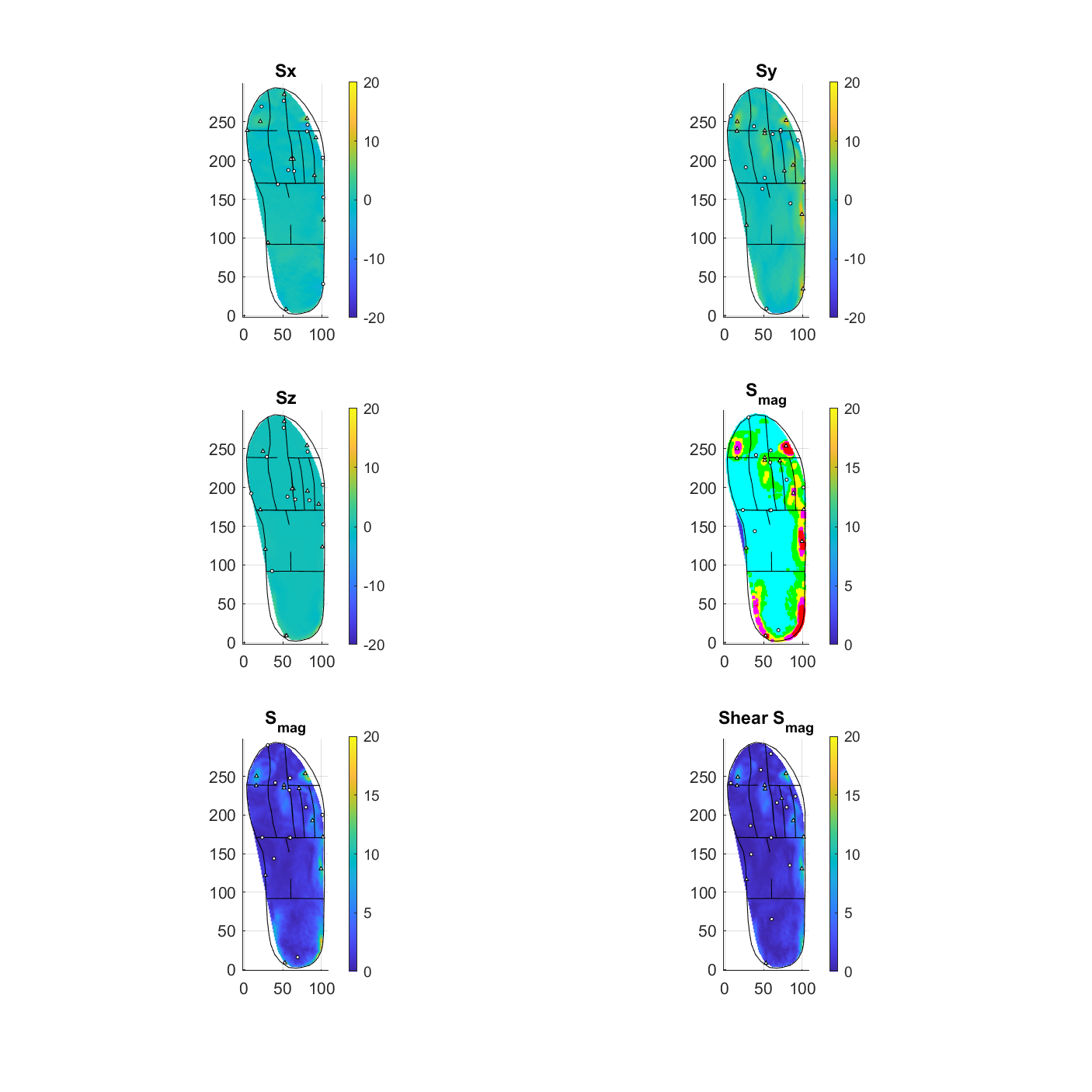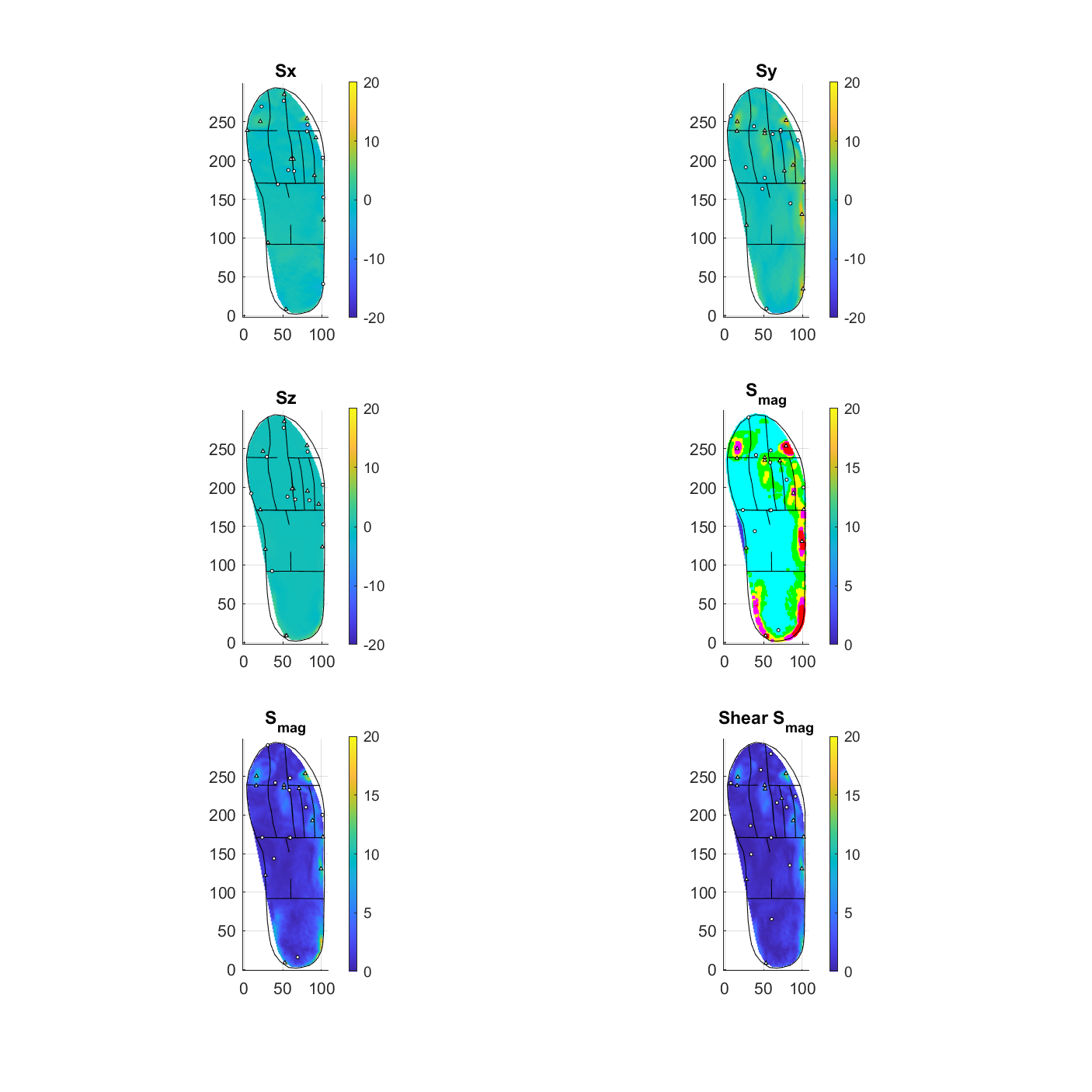 | | 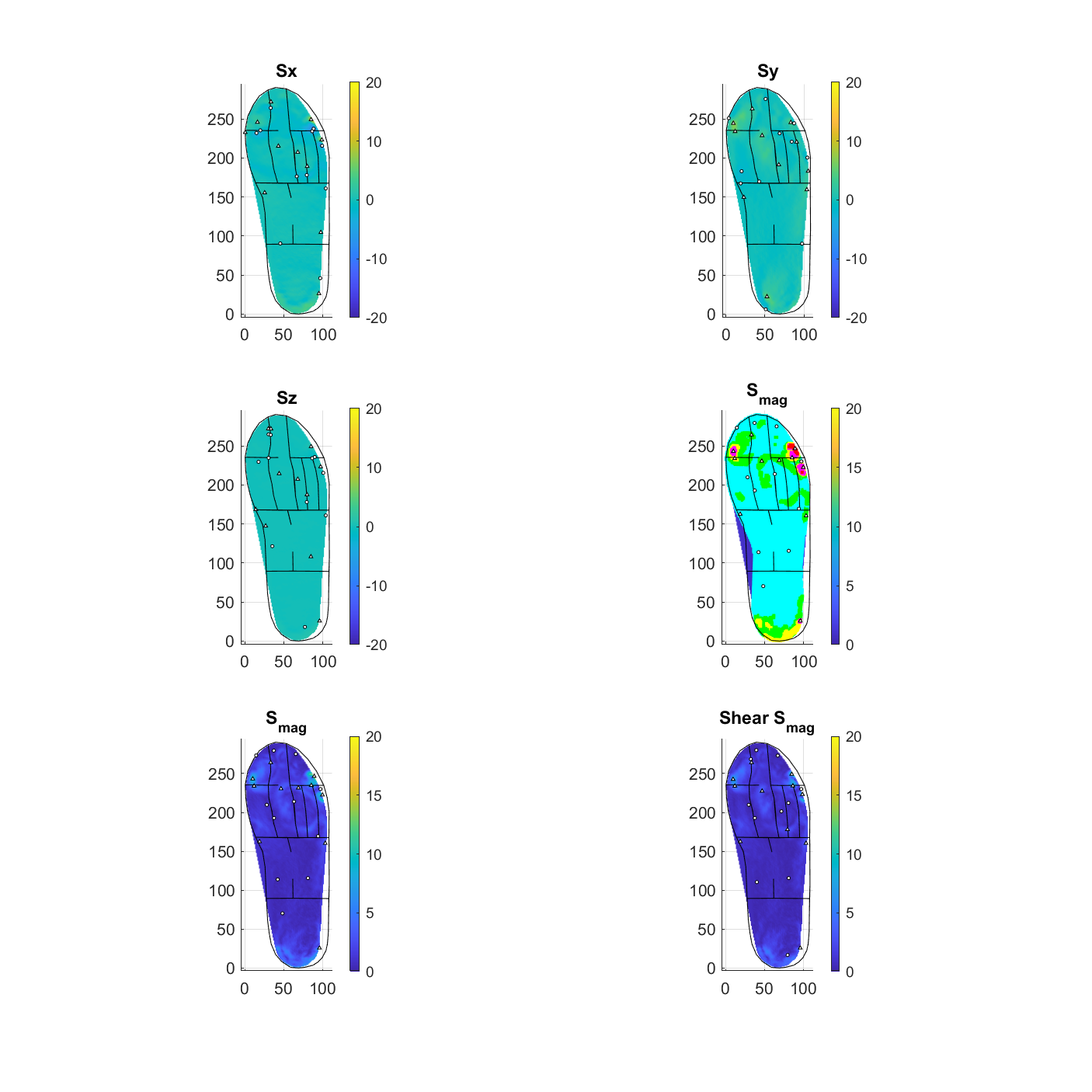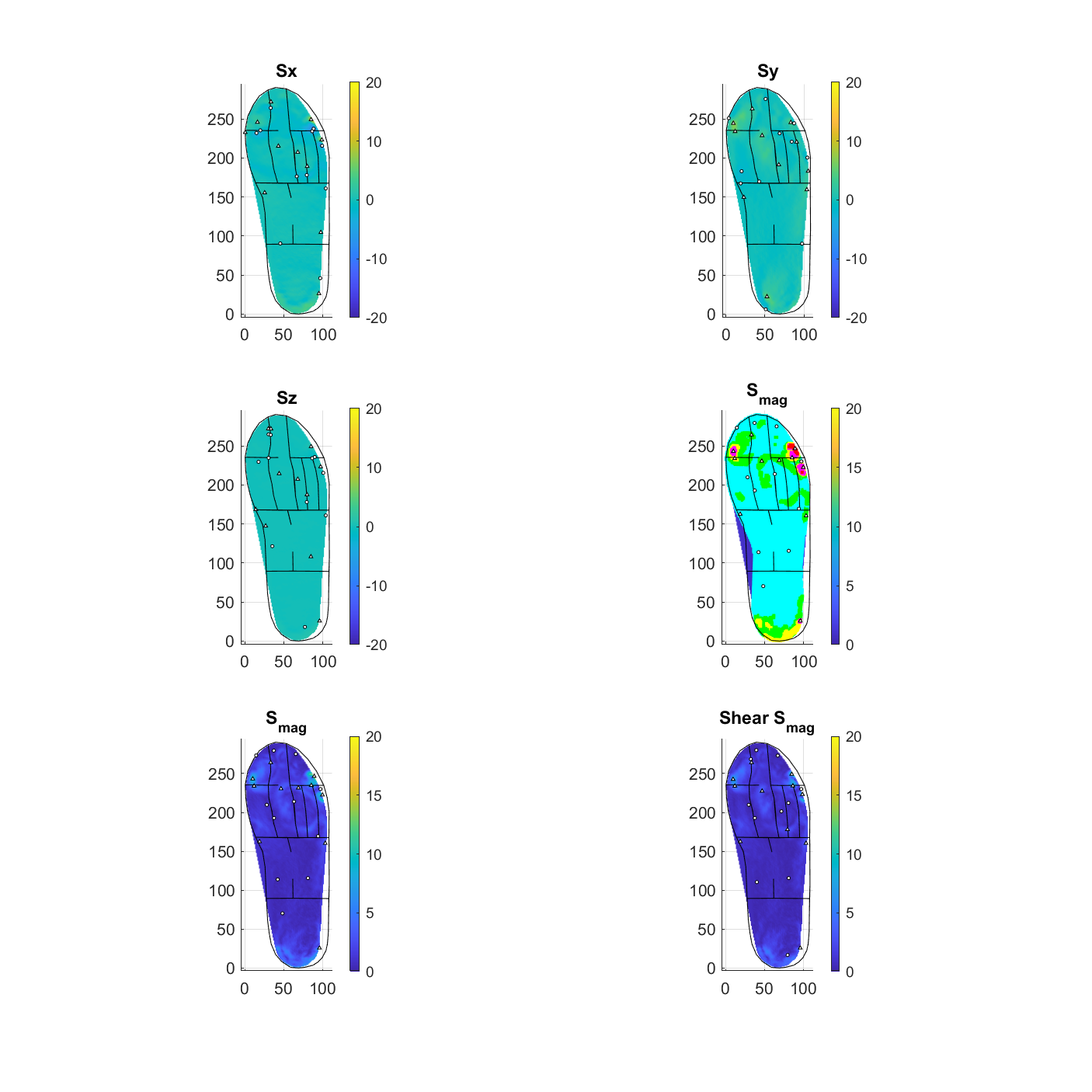 | |
| P05 | 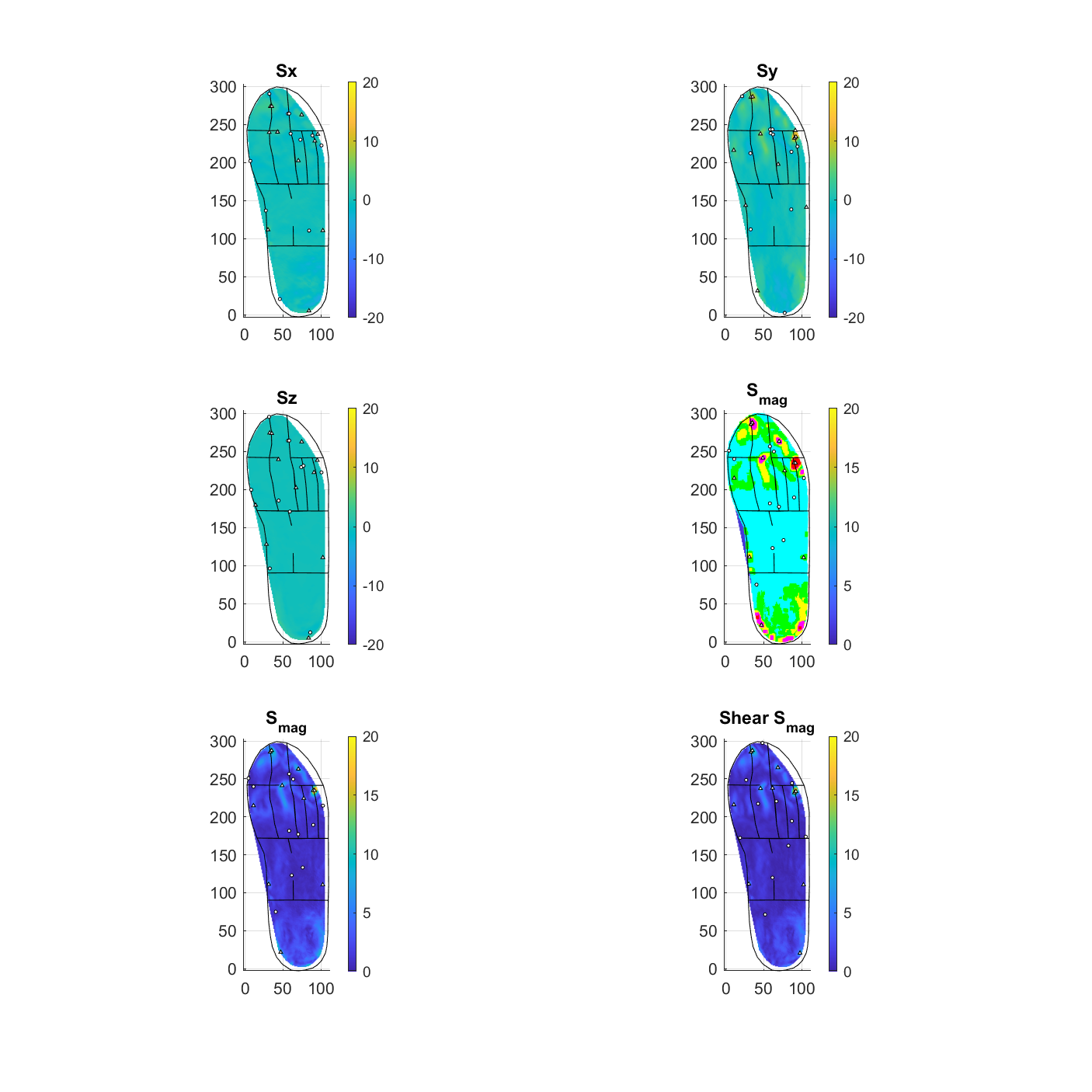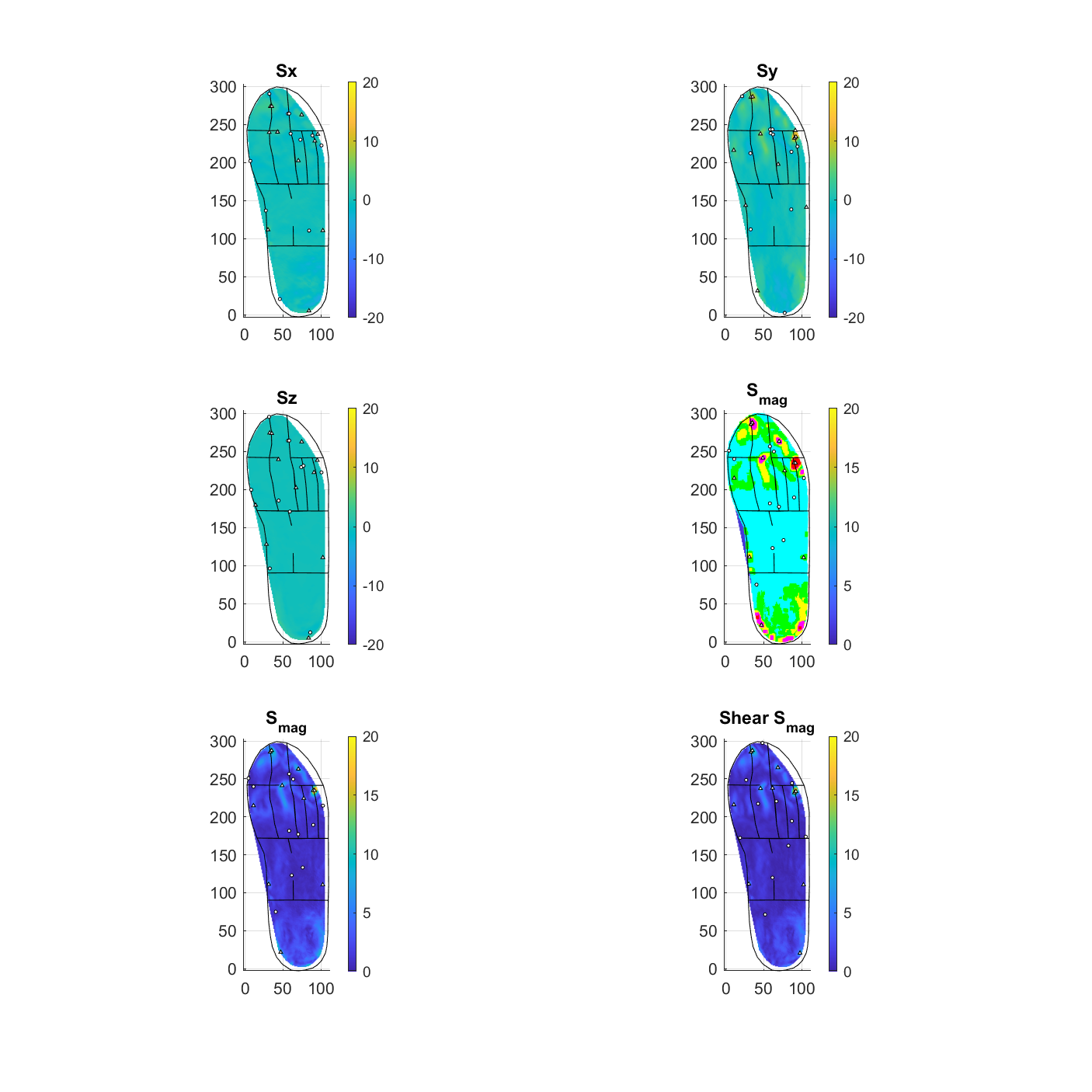 | | 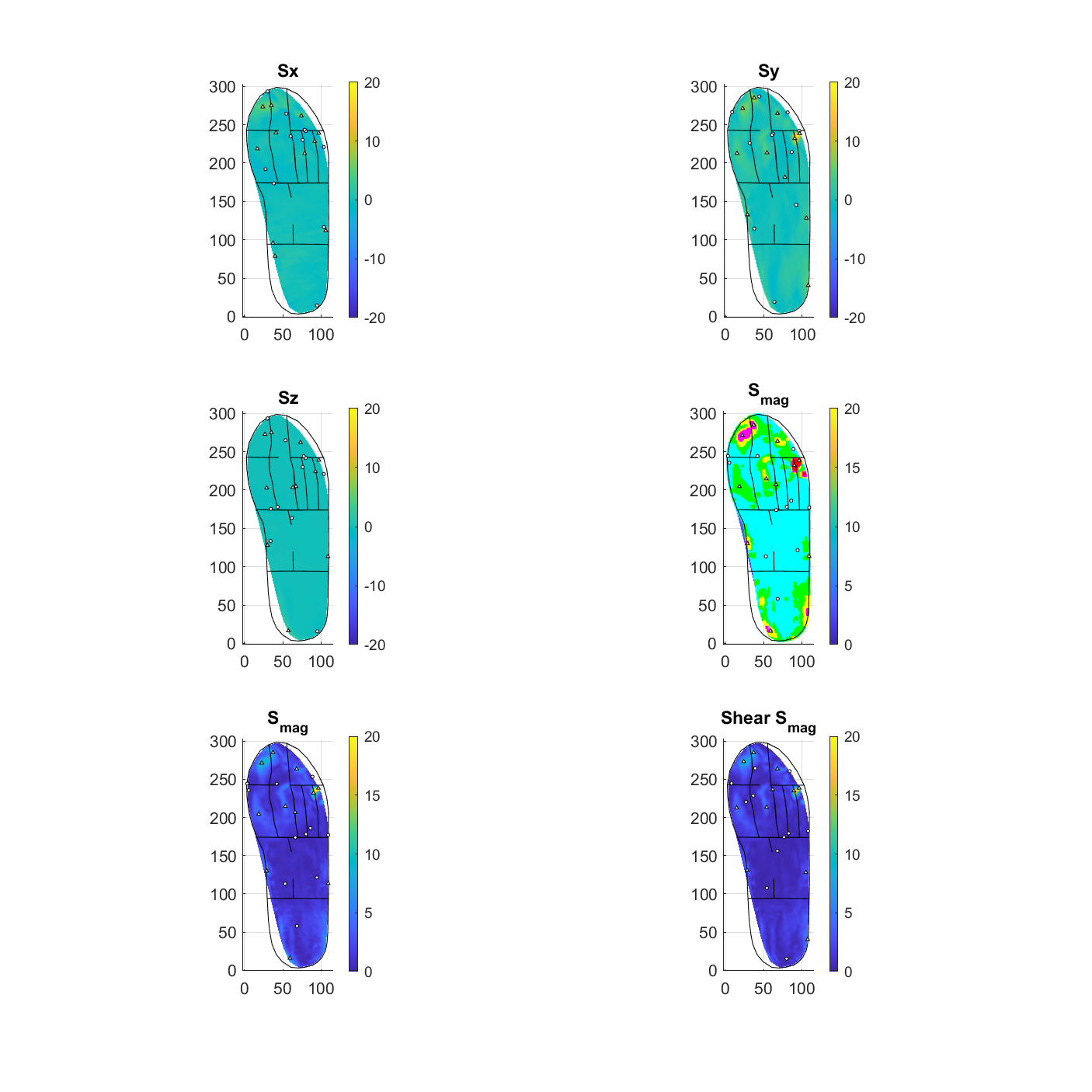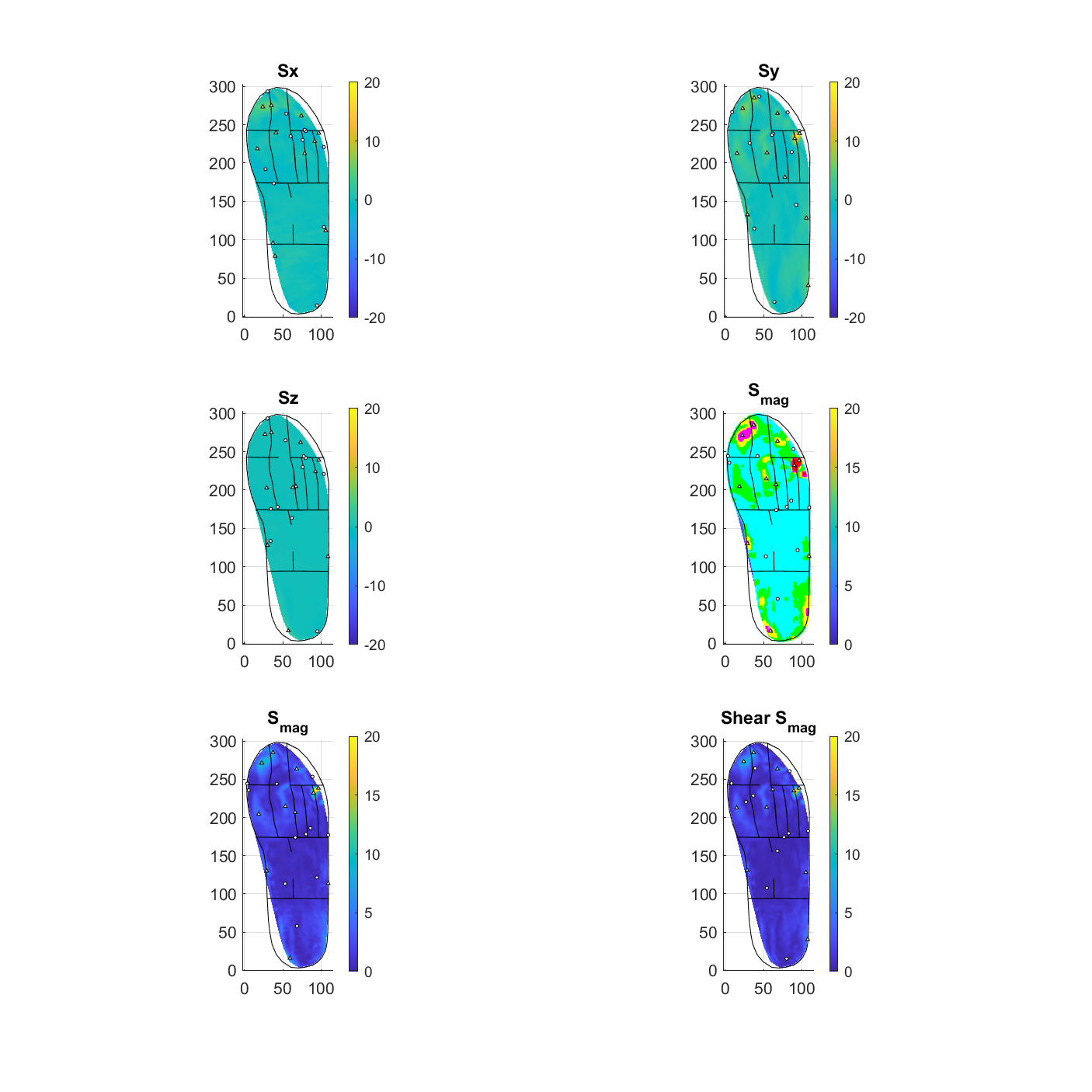 | | 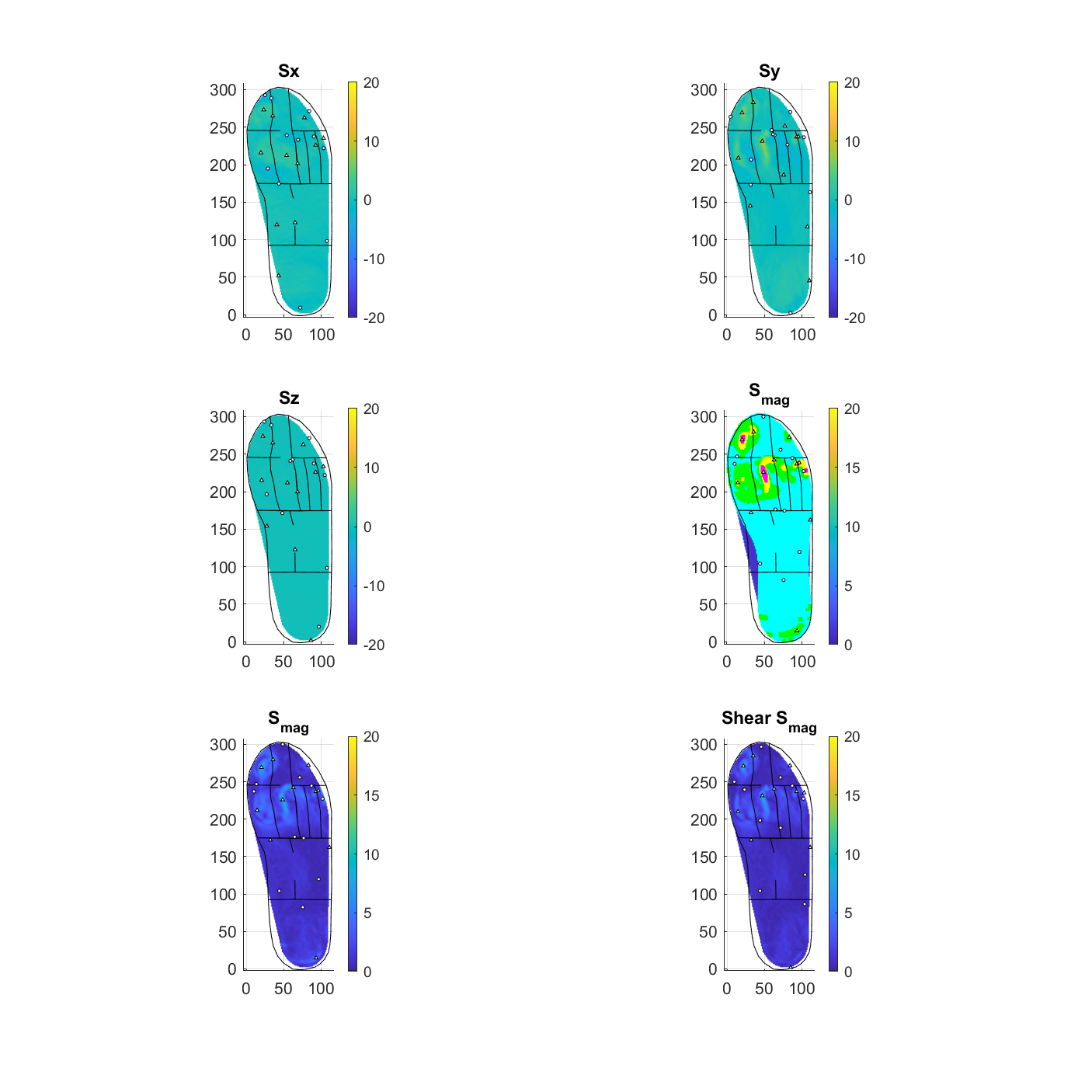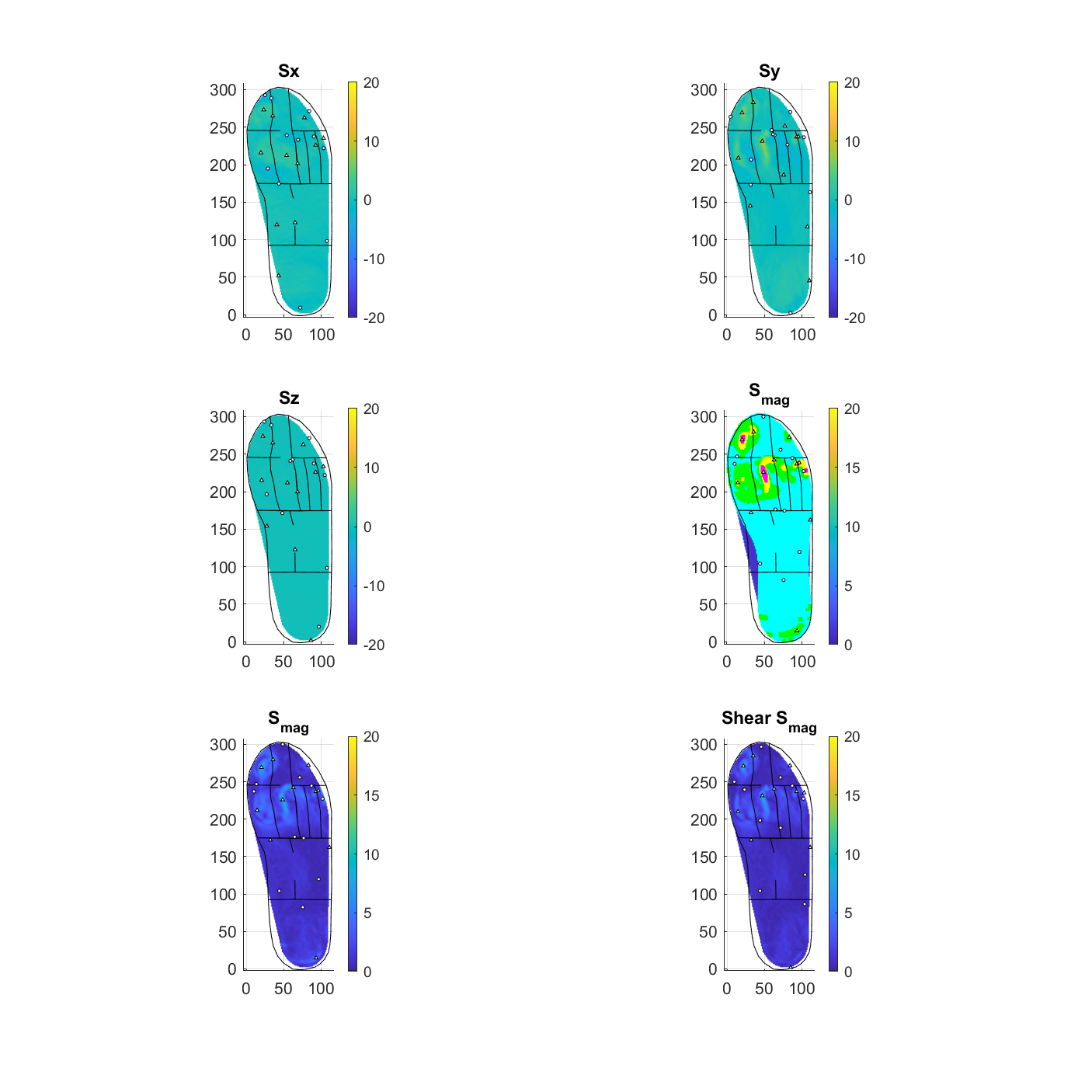 | |

**Supplementary Figure 2.** Representative strain maps for S_MAG_, trial 3 across each participant, with absolute plots demonstrating strain brackets: 0-2.5% (blue), 2.5-5% (green), 5-7.5% (yellow), 7.5-10% (magenta) and > 10 (red).

|  | **Low Stiffness** | **High Stiffness** | **STAMPS** |
| --- | --- | --- | --- |
|  | **Strain Map (S_X_)** | **Strain Map (S_X_)** | **Strain Map (S_X_)** |
| P01 | 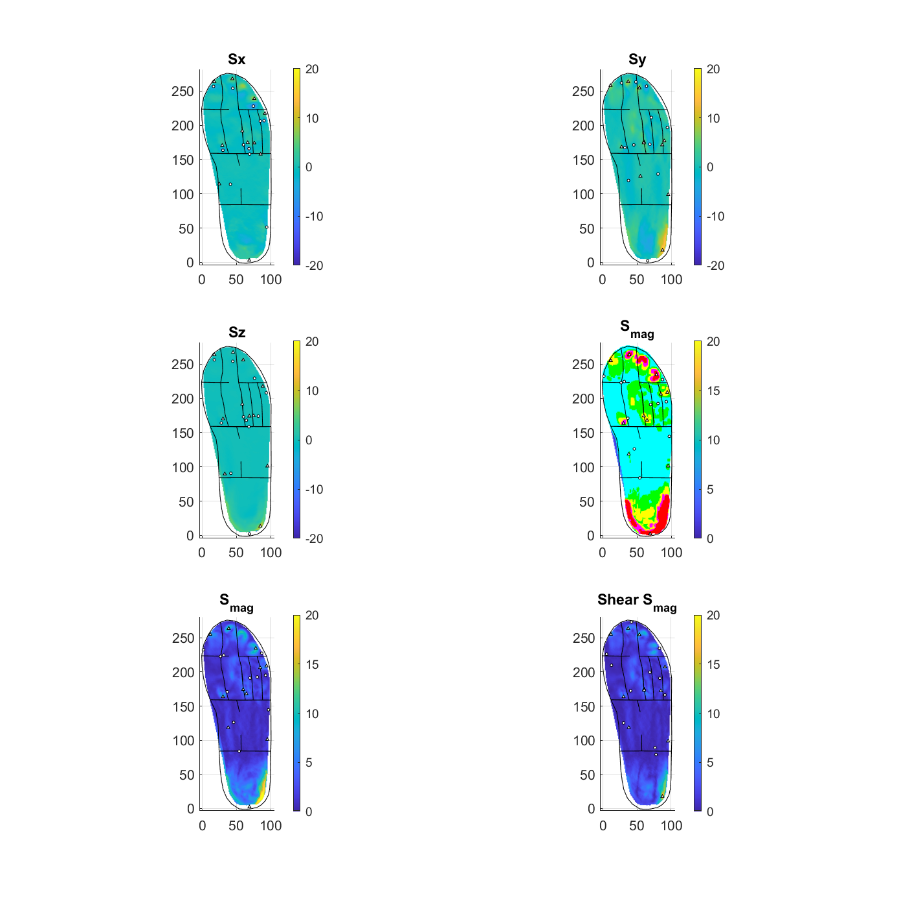 | 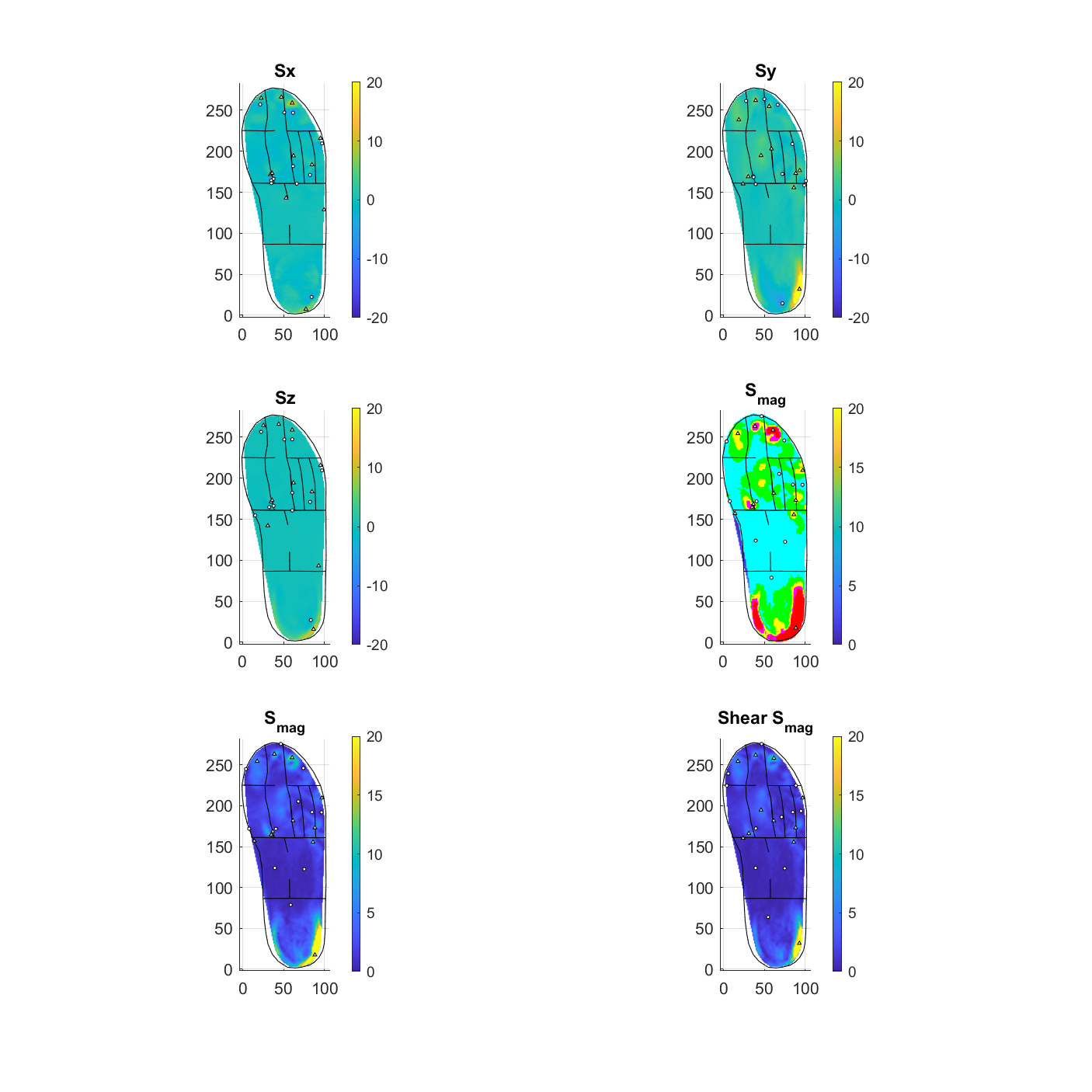 | 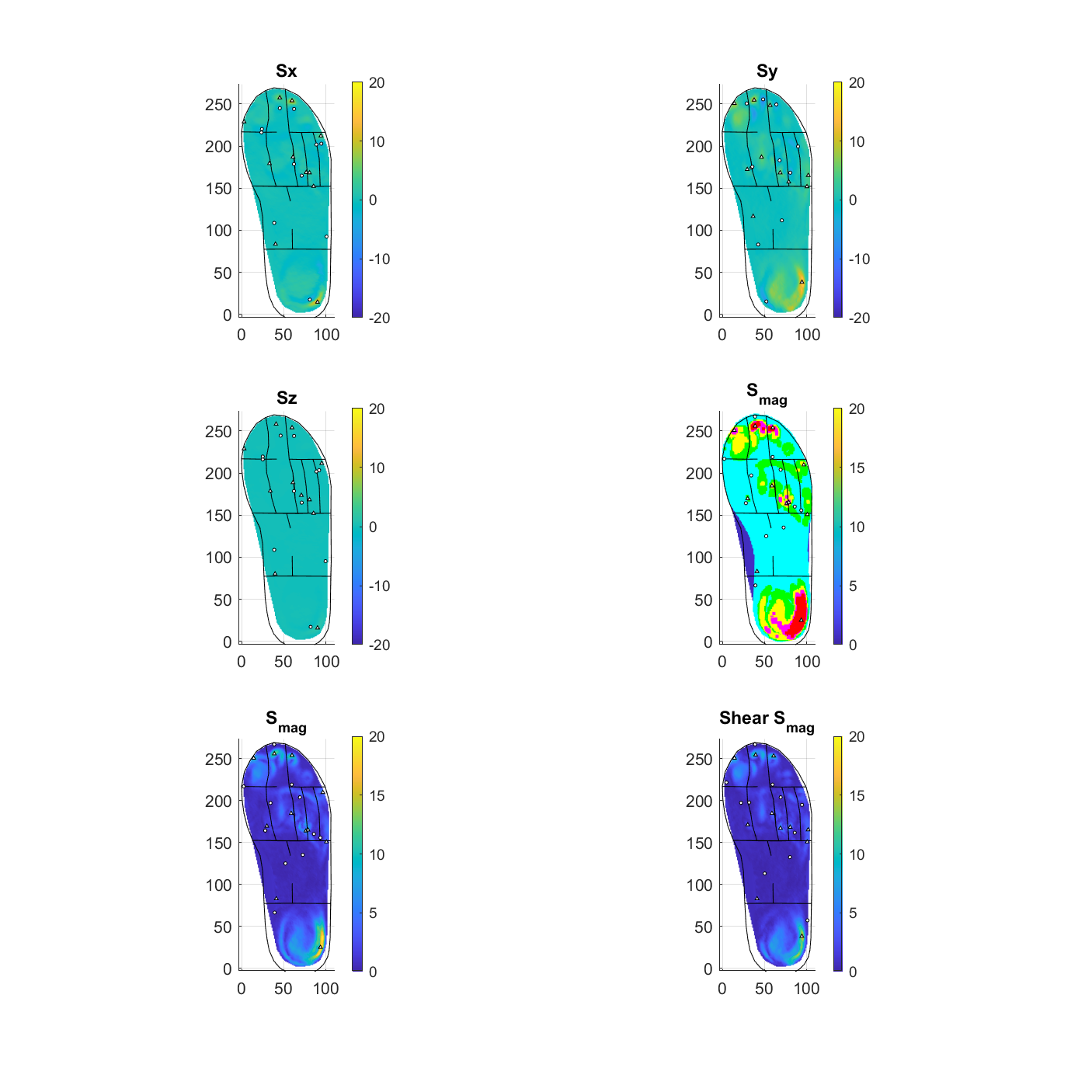 |
| P02 | 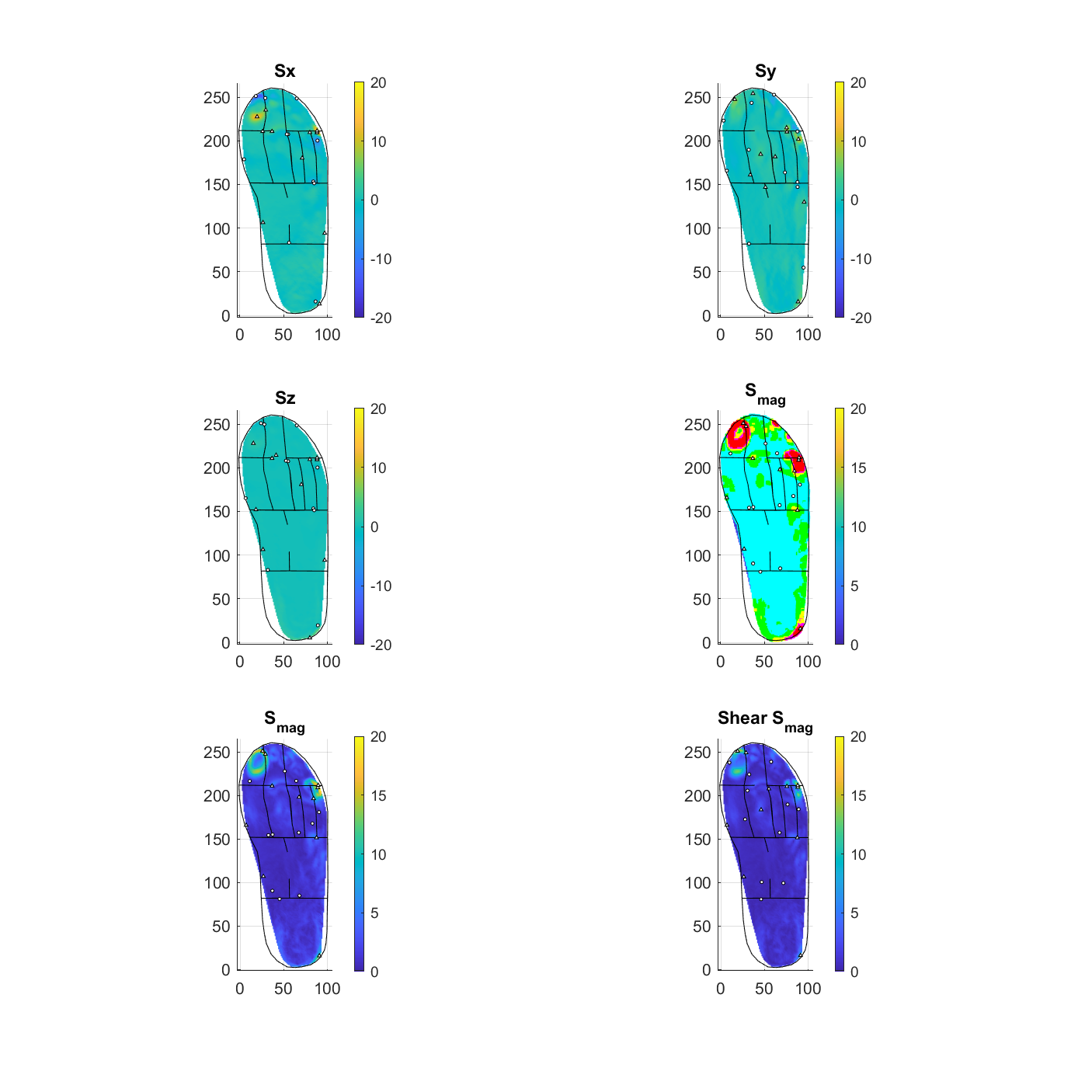 | 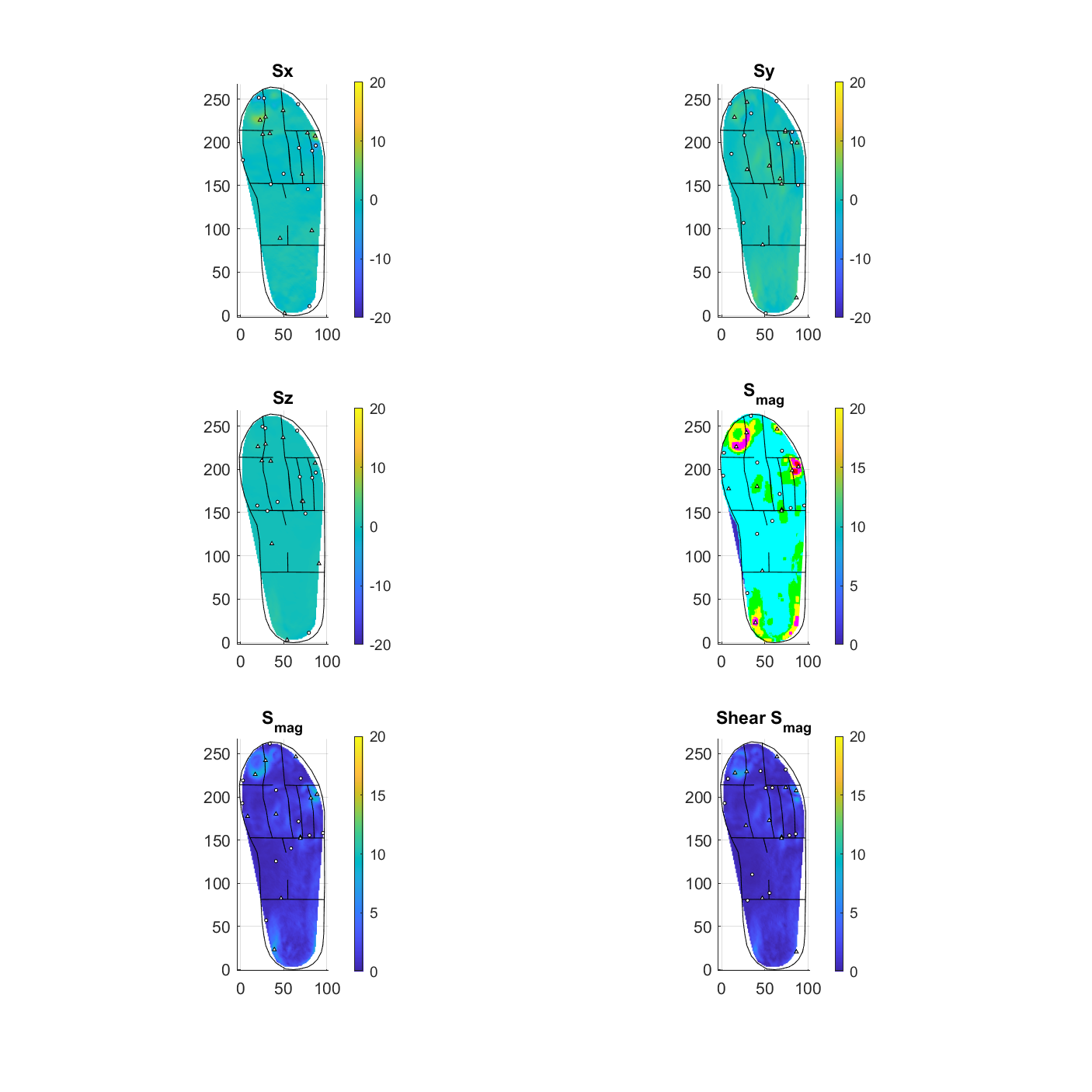 | 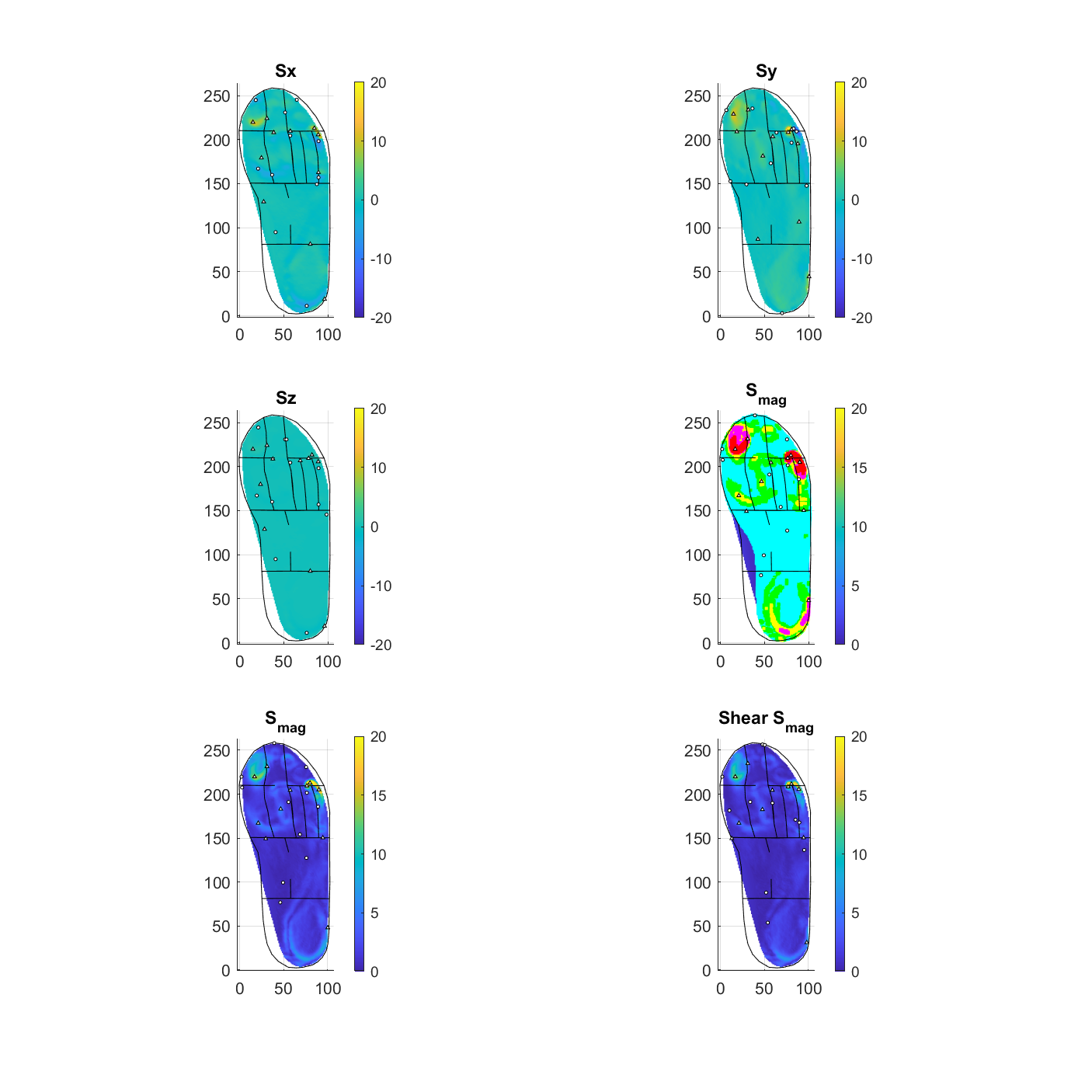 |
| P03 | 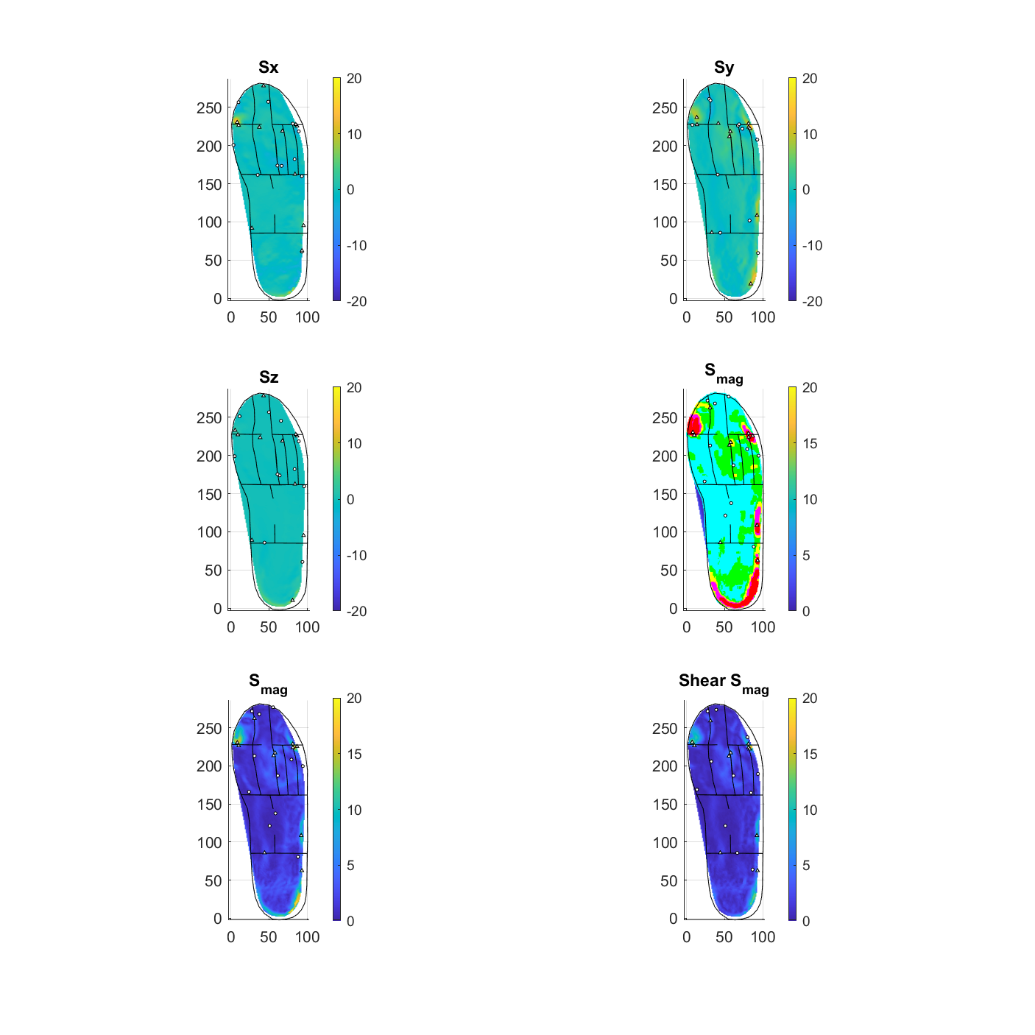 | 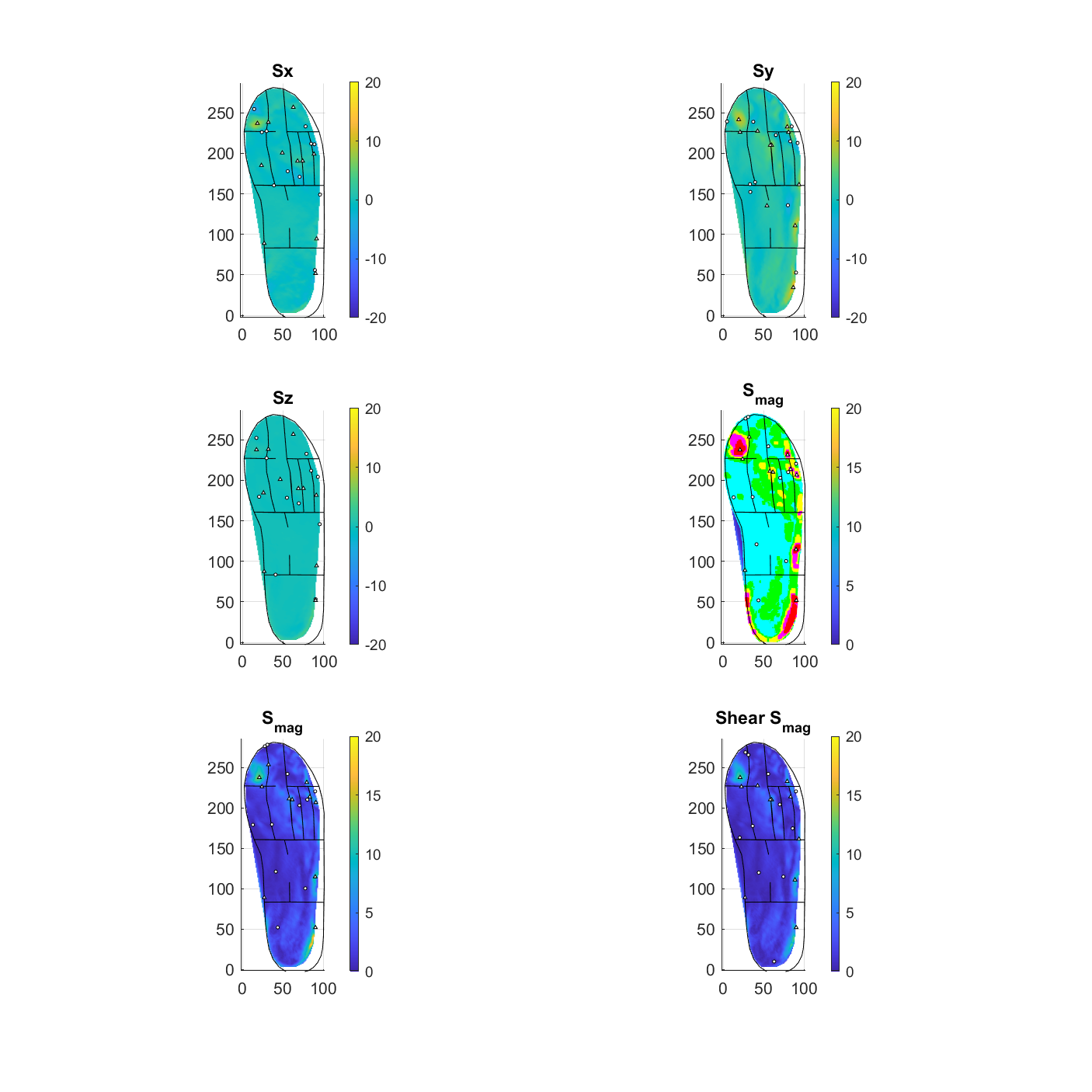 | 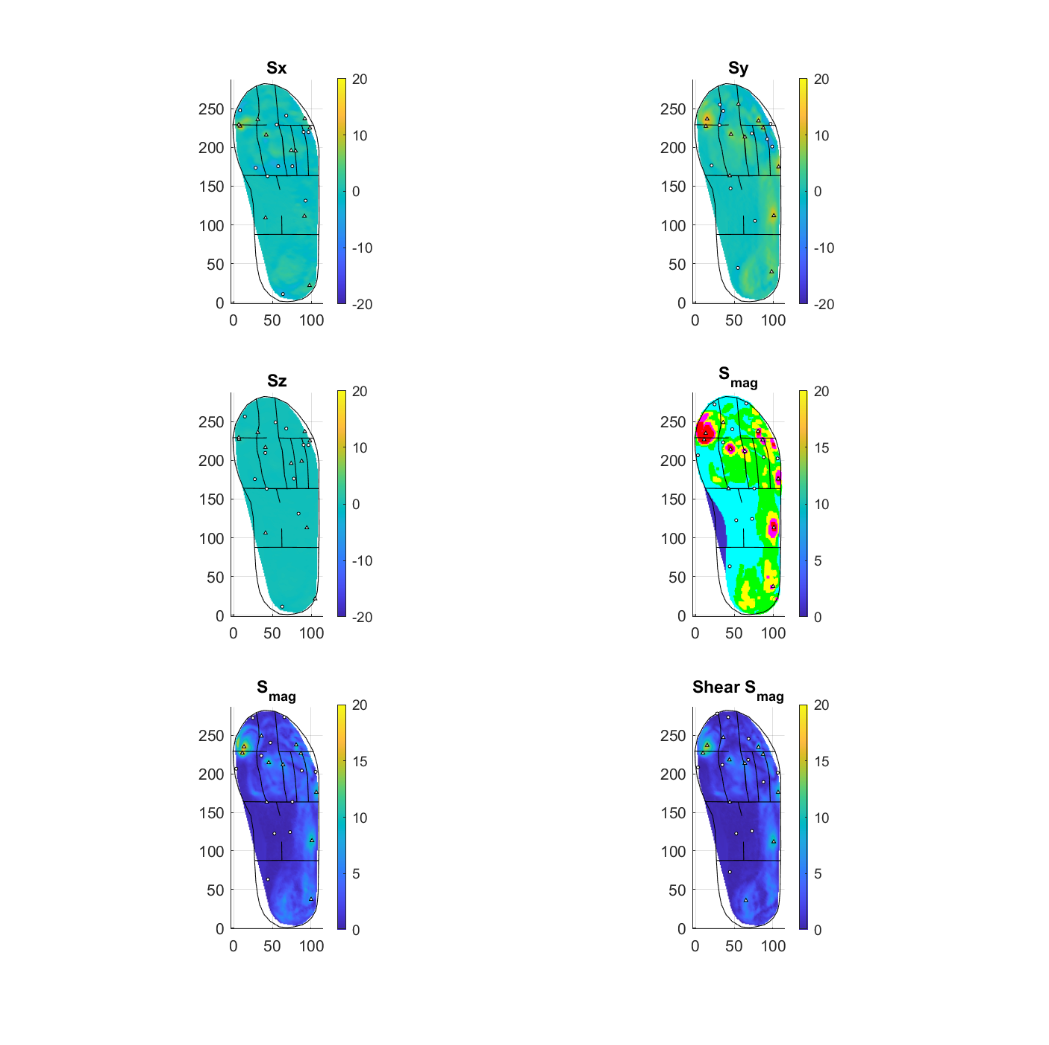 |
| P04 | 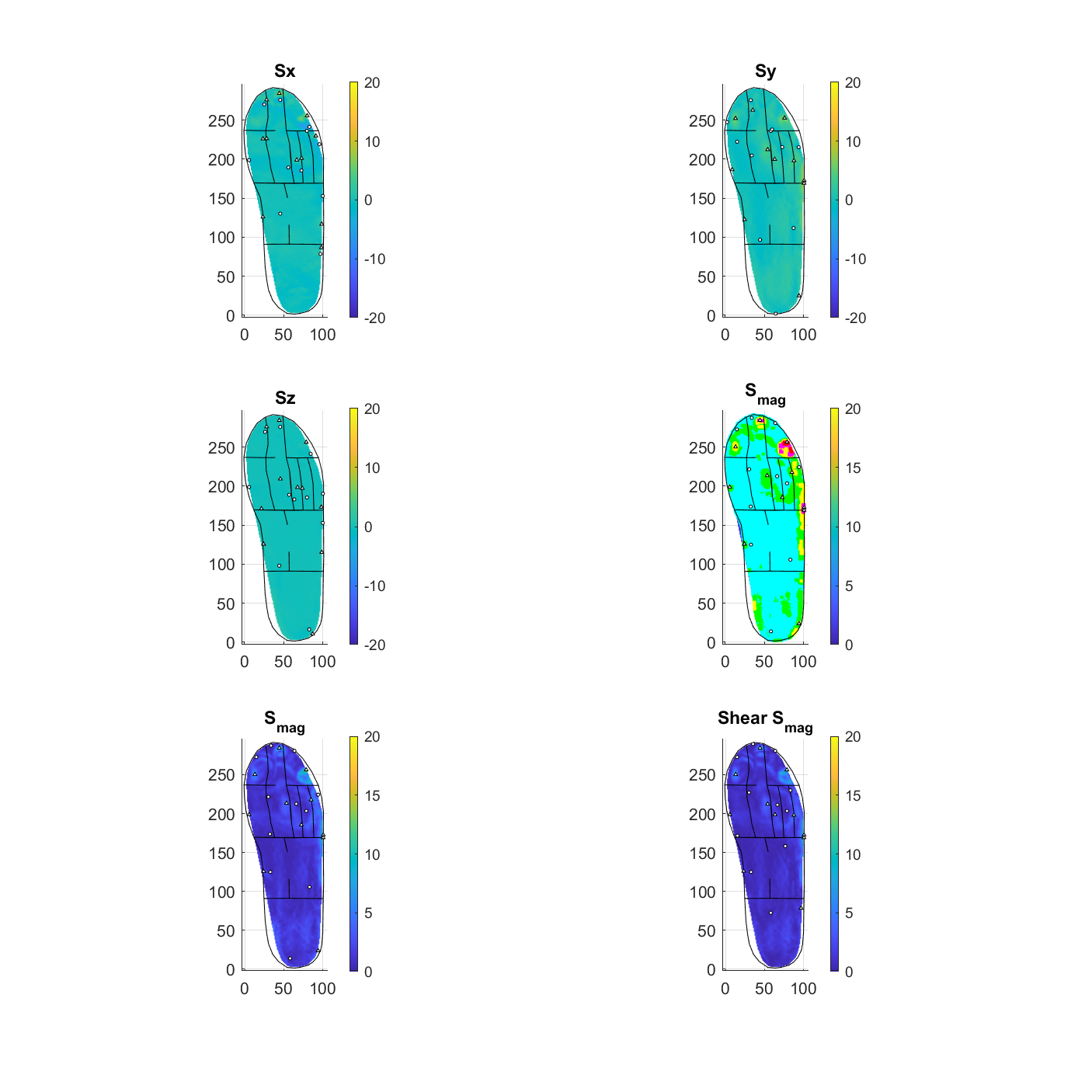 | 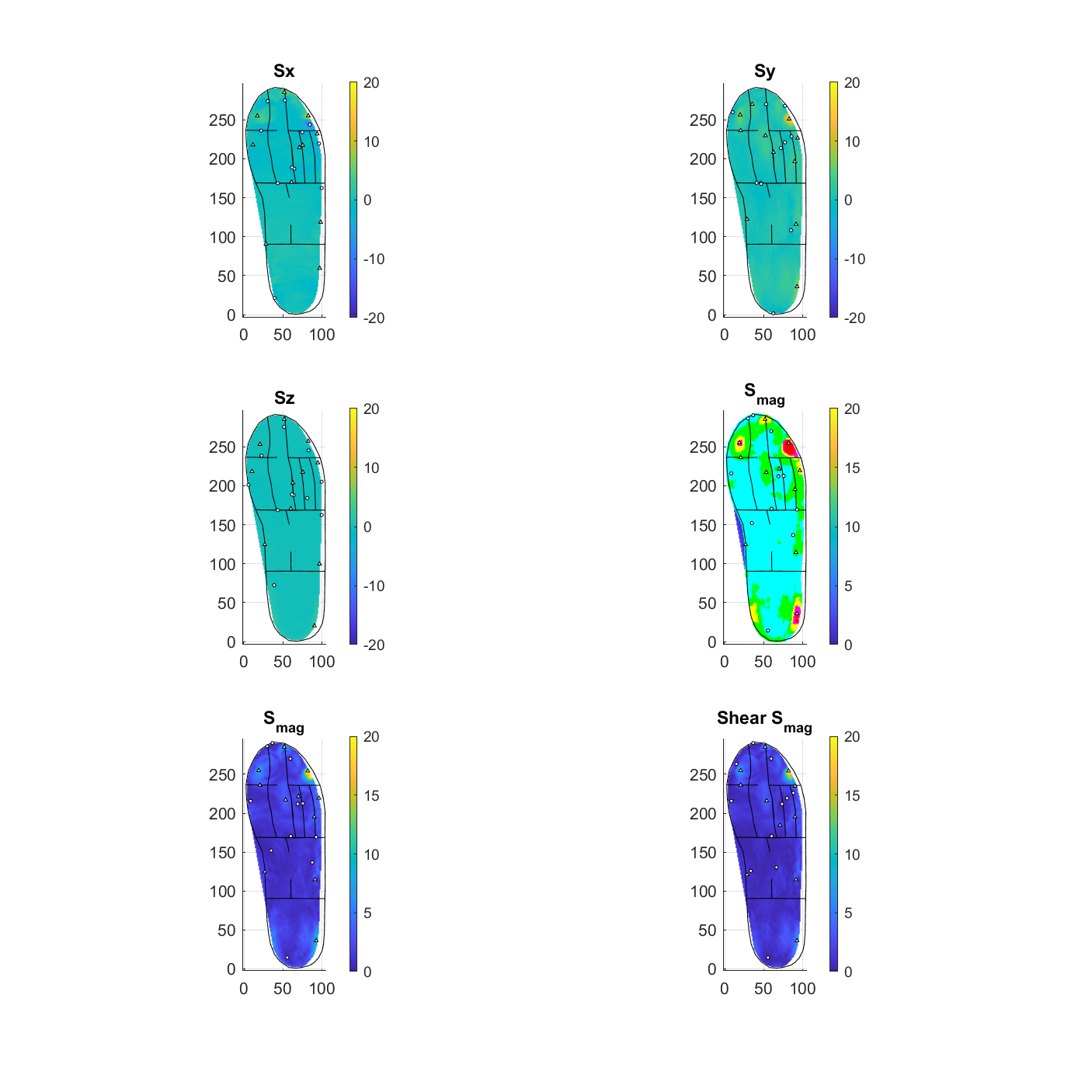 | 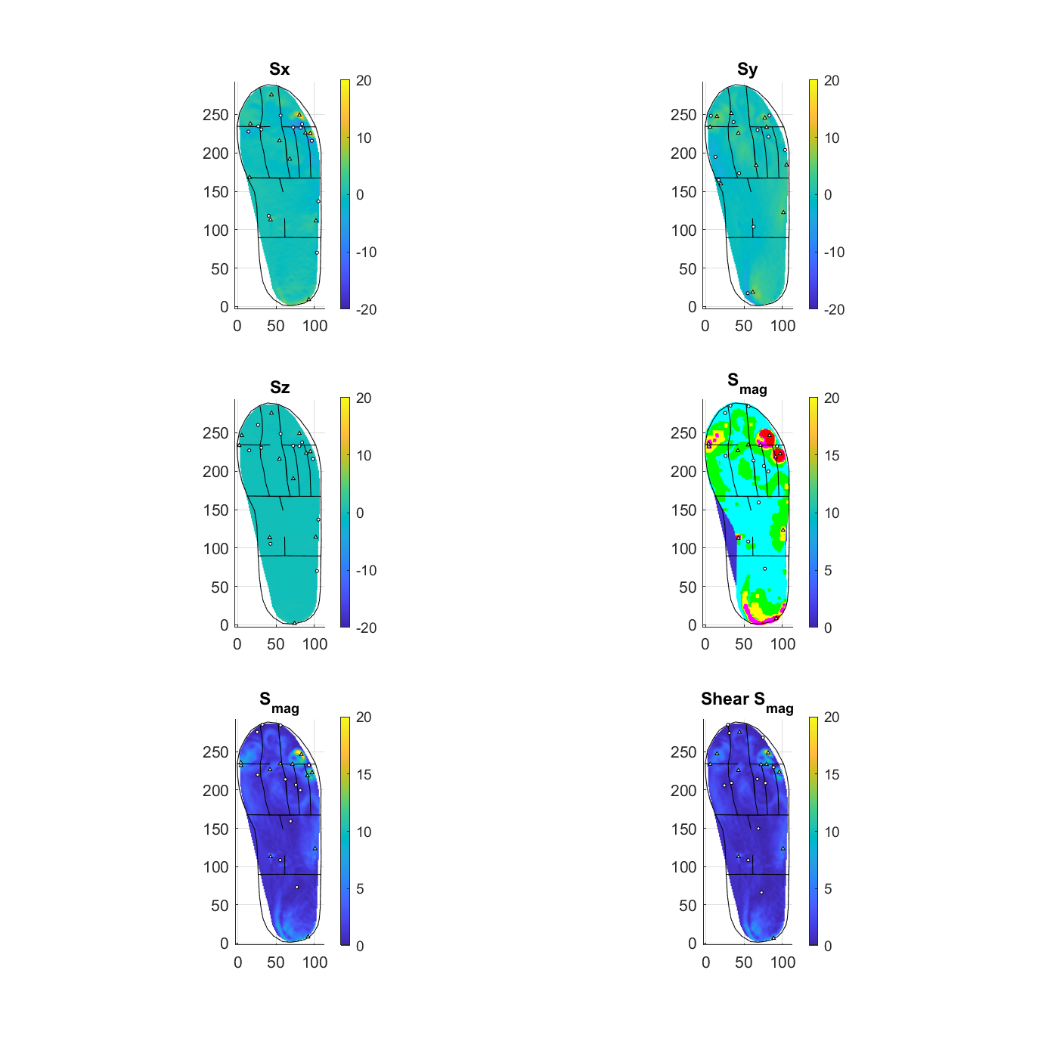 |
| P05 | 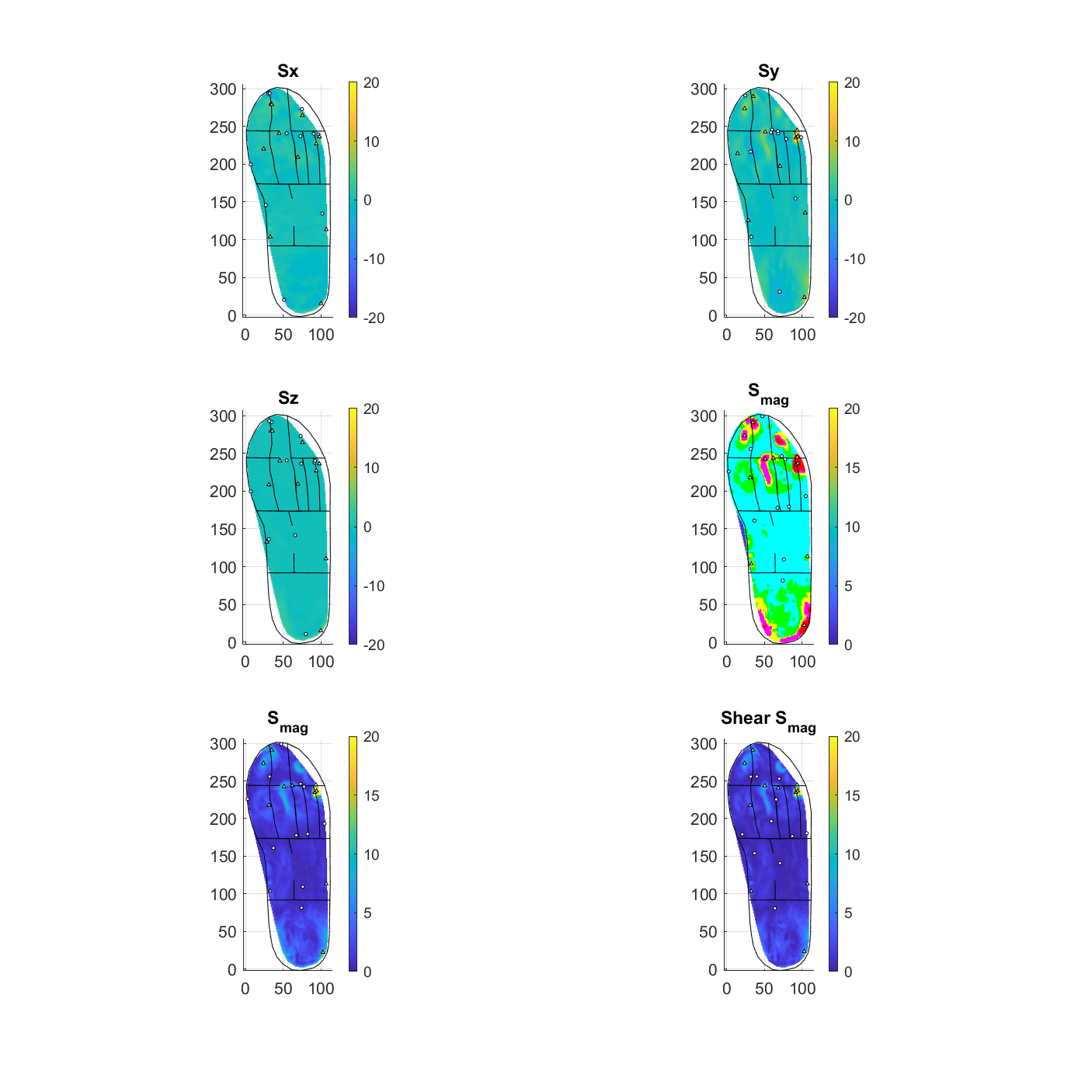 | 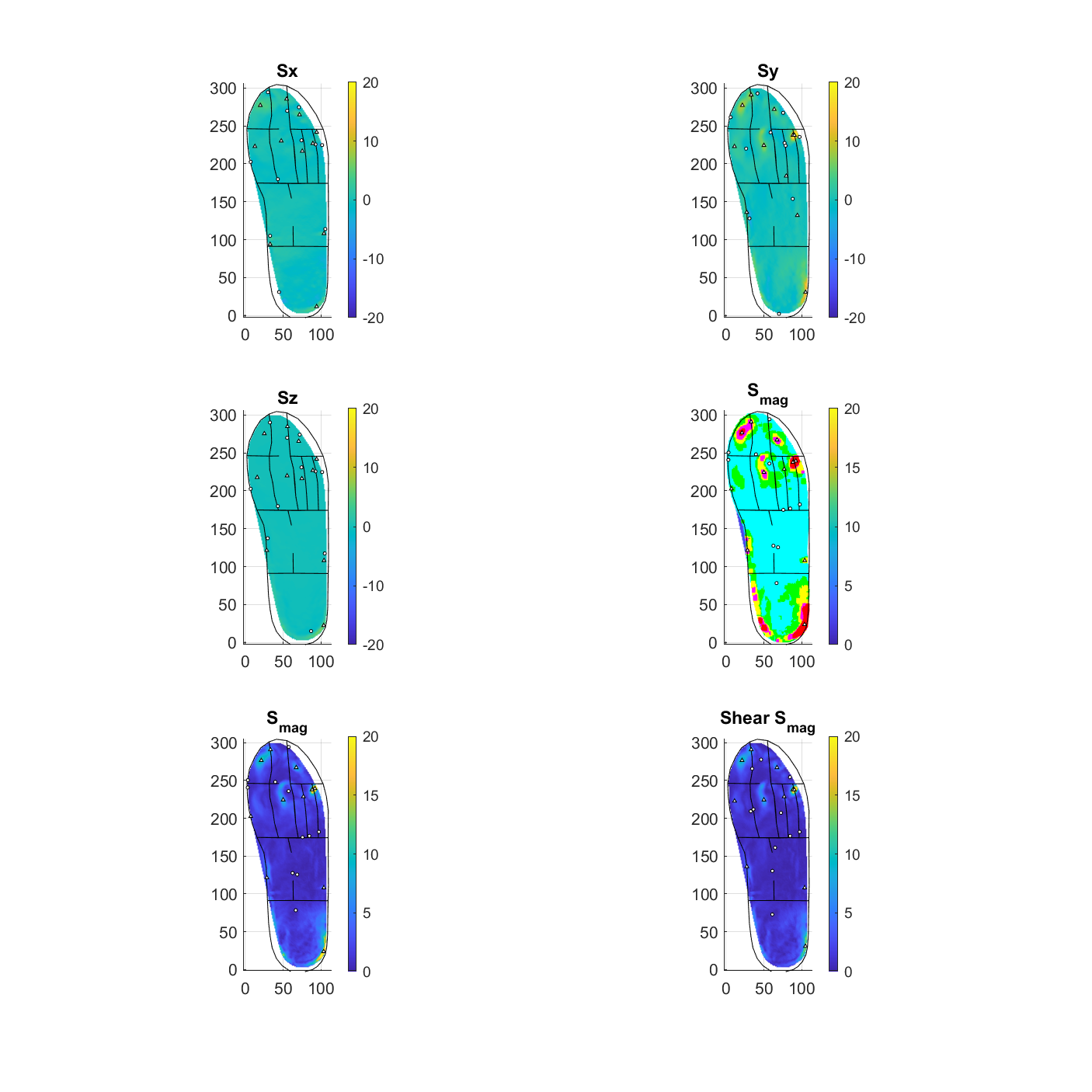 | 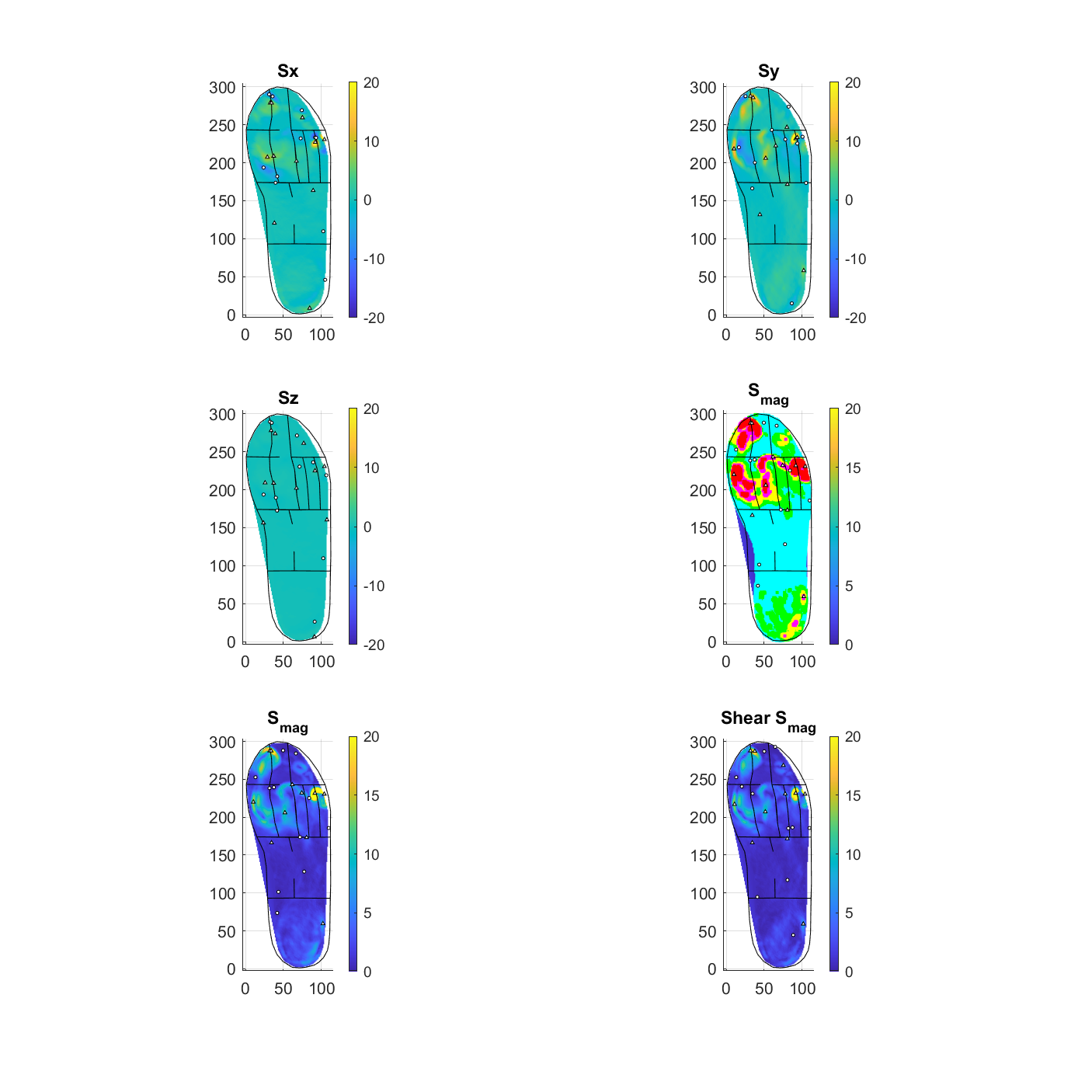 |

**Supplementary Figure 3.** Representative strain maps for S_X_, trial 1 across each participant and condition.

|  | **Low Stiffness** | **High Stiffness** | **STAMPS** |
| --- | --- | --- | --- |
|  | **Strain Map (S_X_)** | **Strain Map (S_X_)** | **Strain Map (S_X_)** |
| P01 | 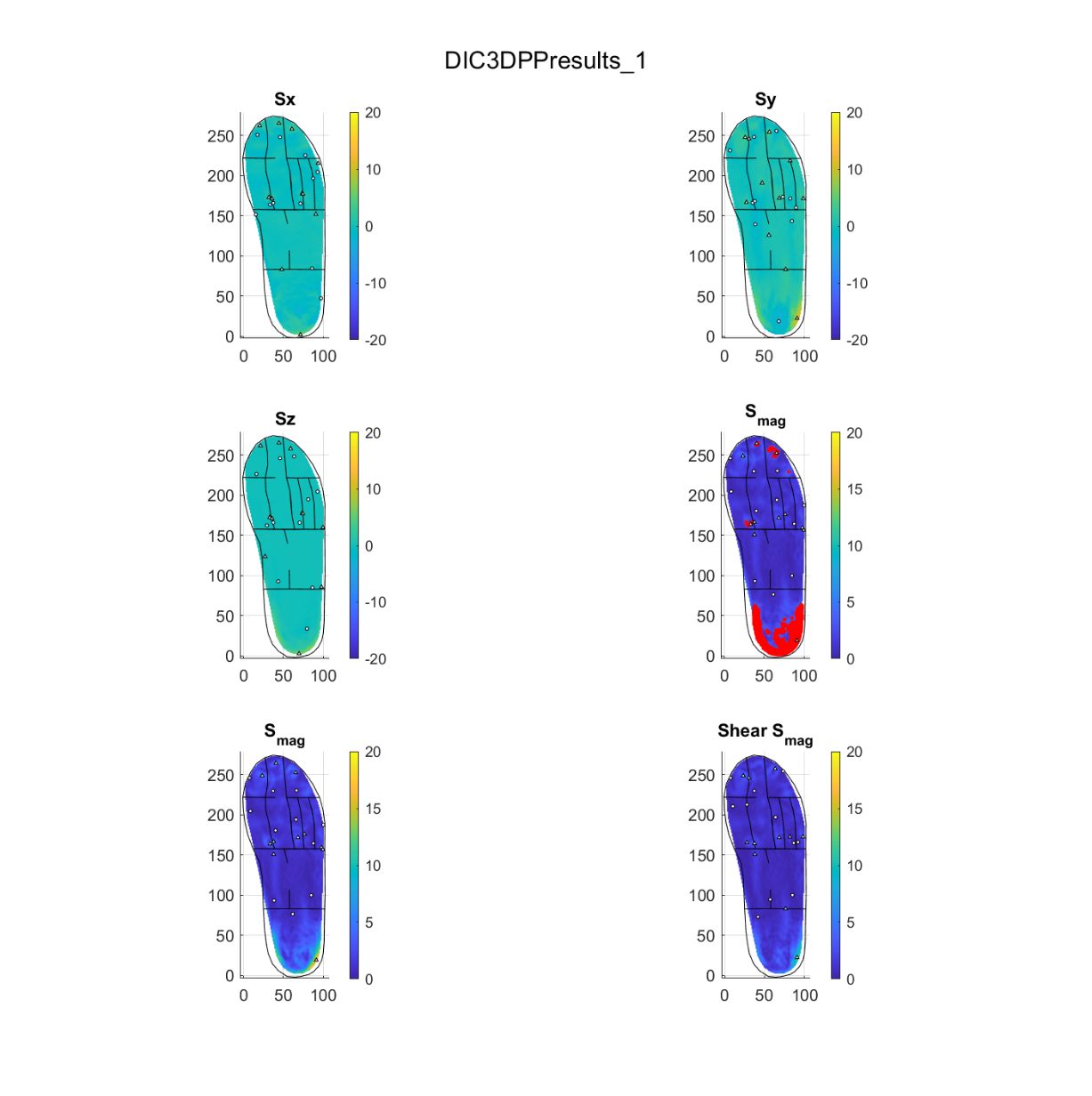 | 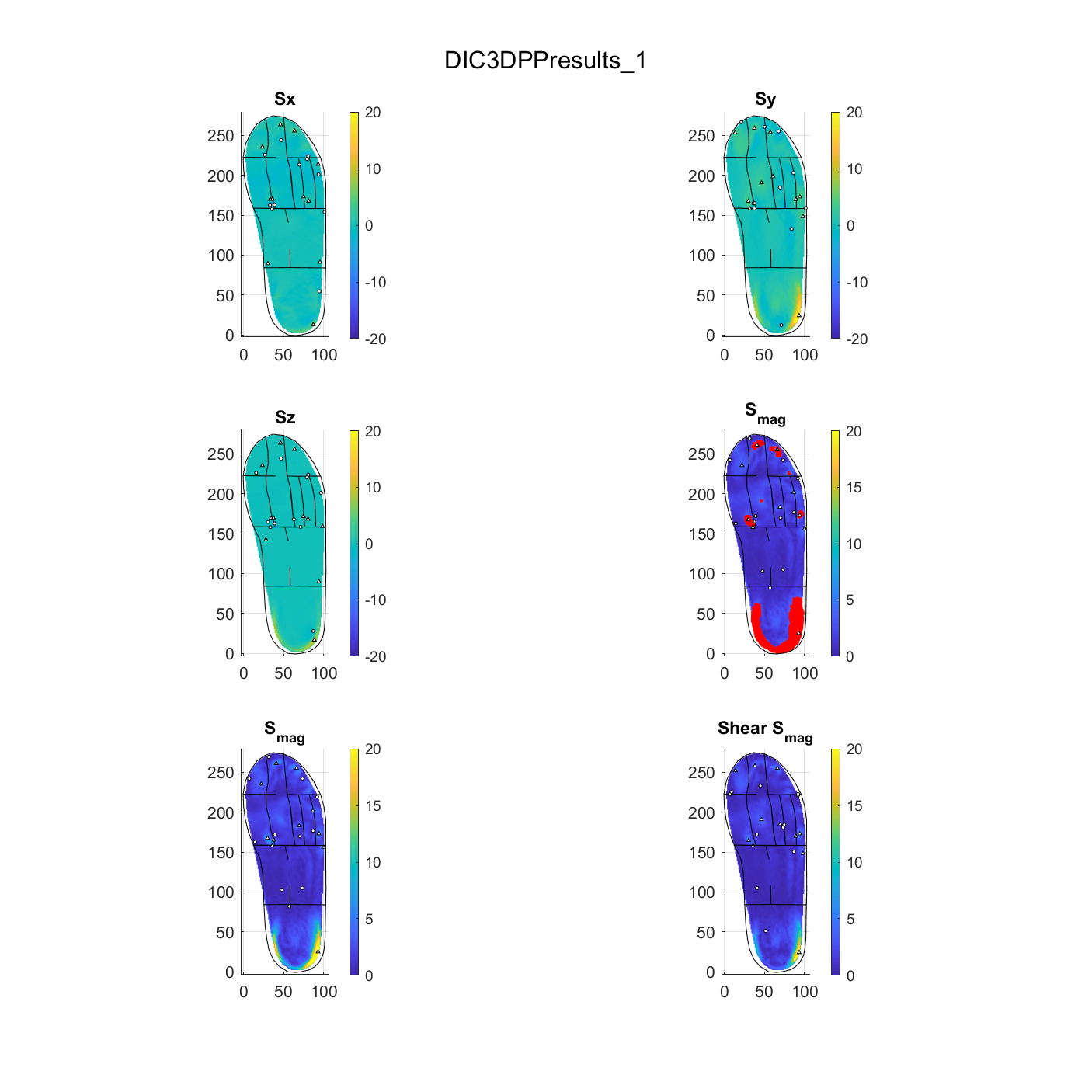 | 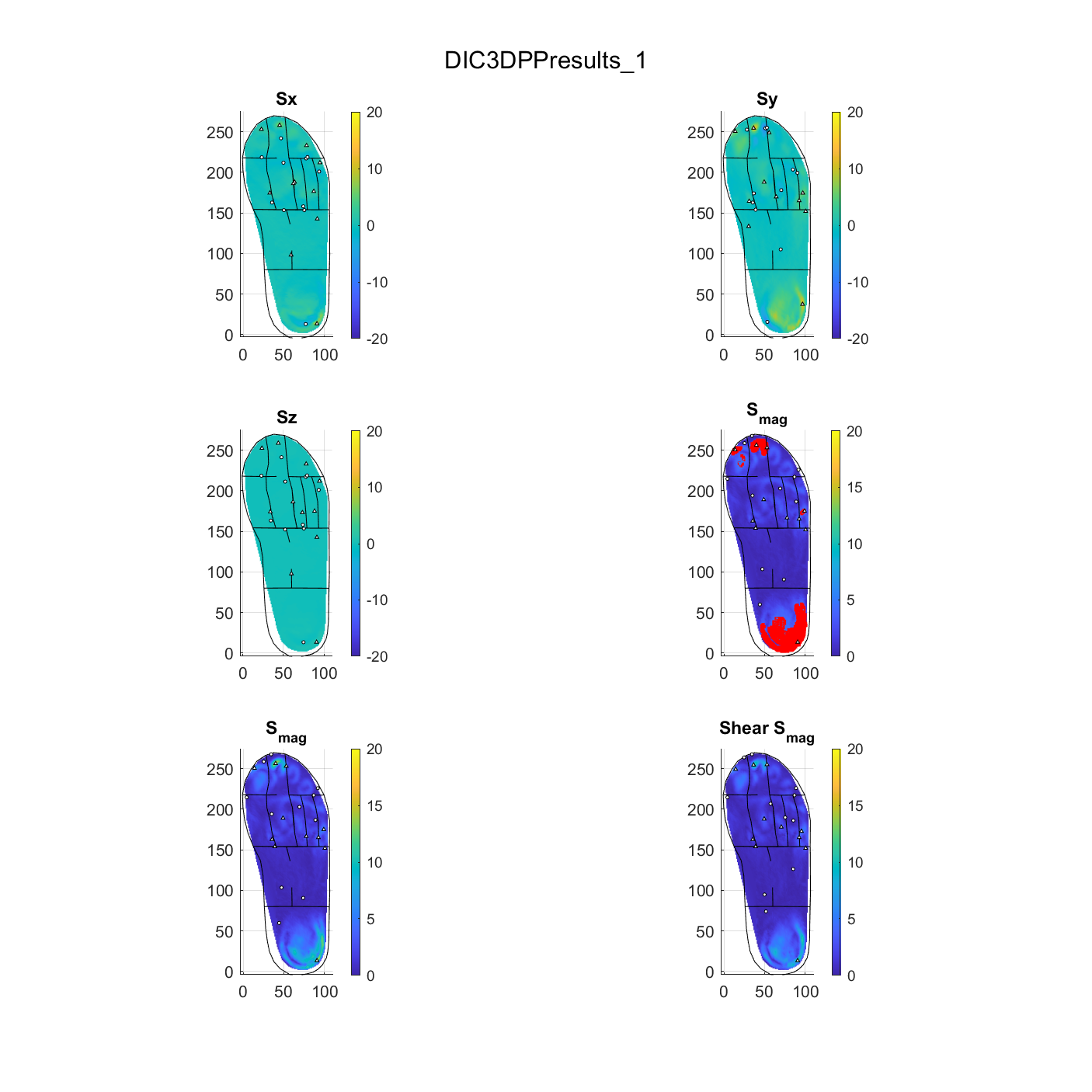 |
| P02 | 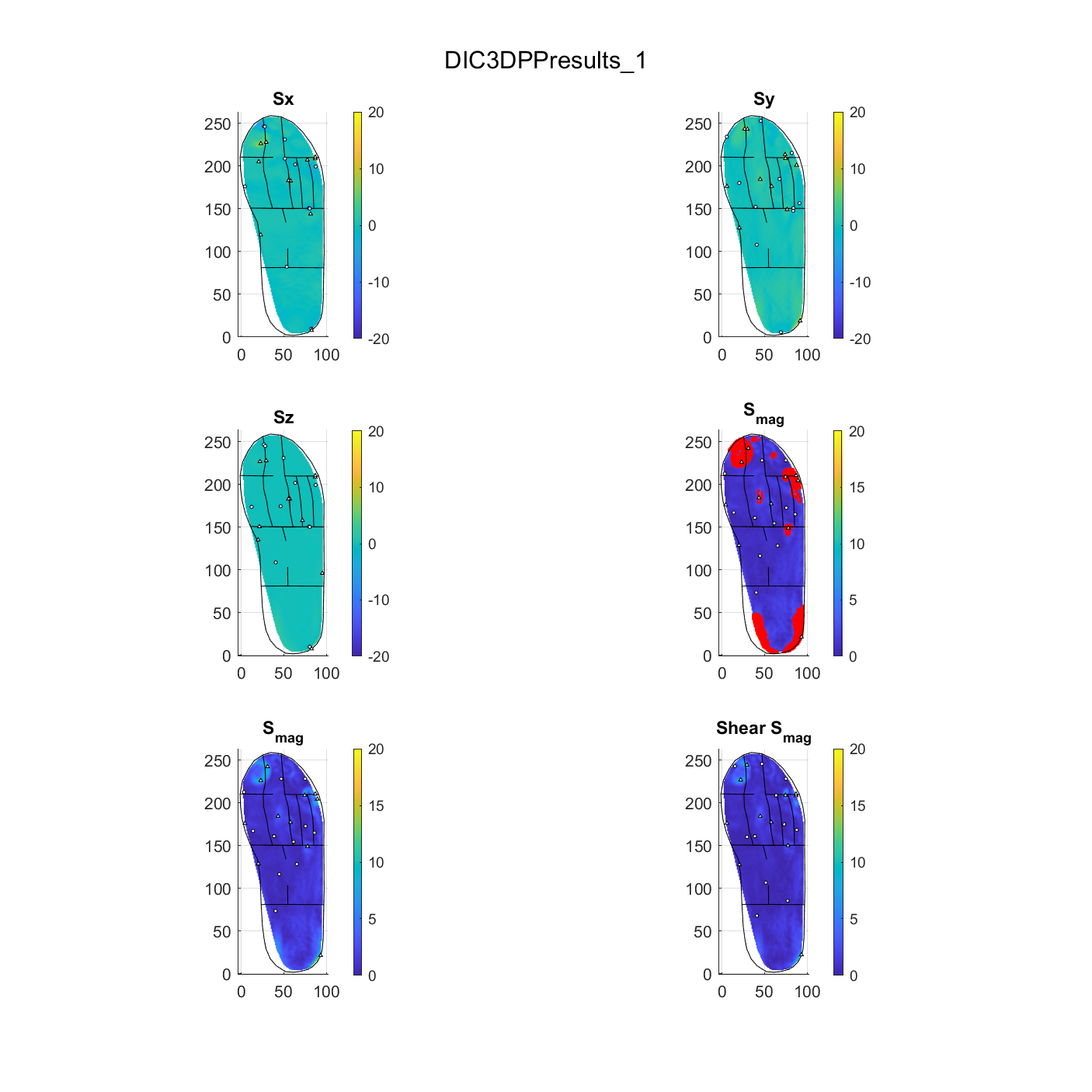 | 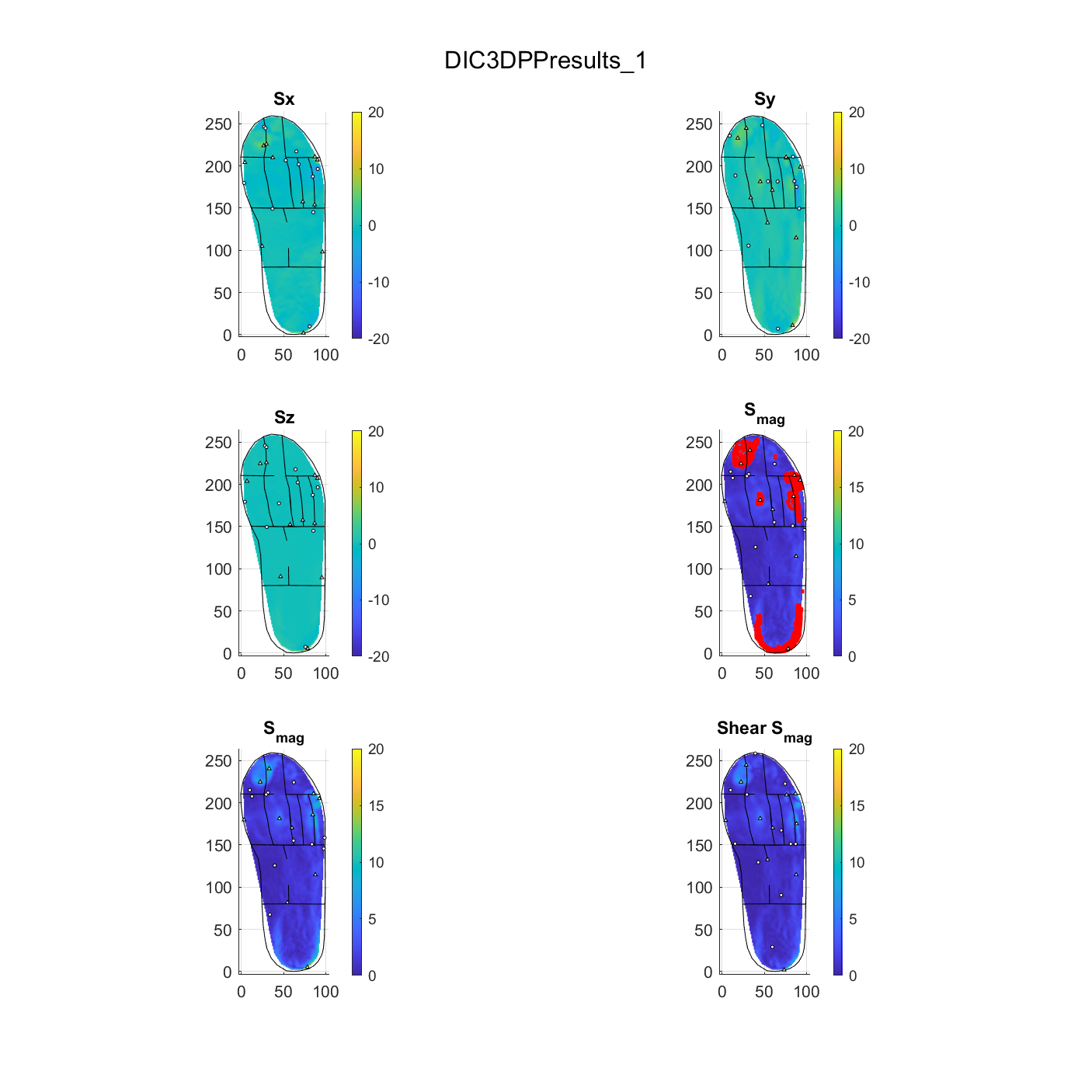 | 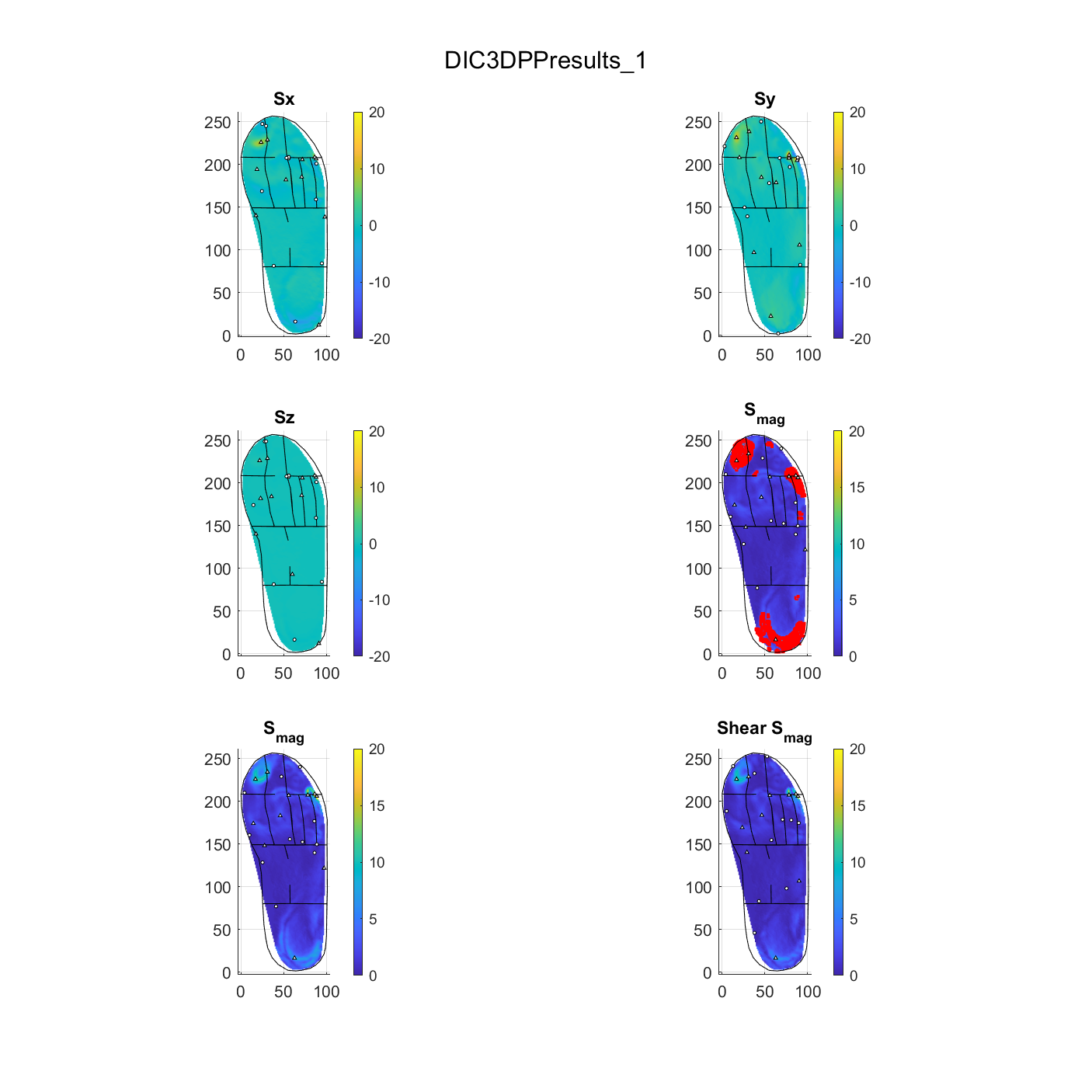 |
| P03 | 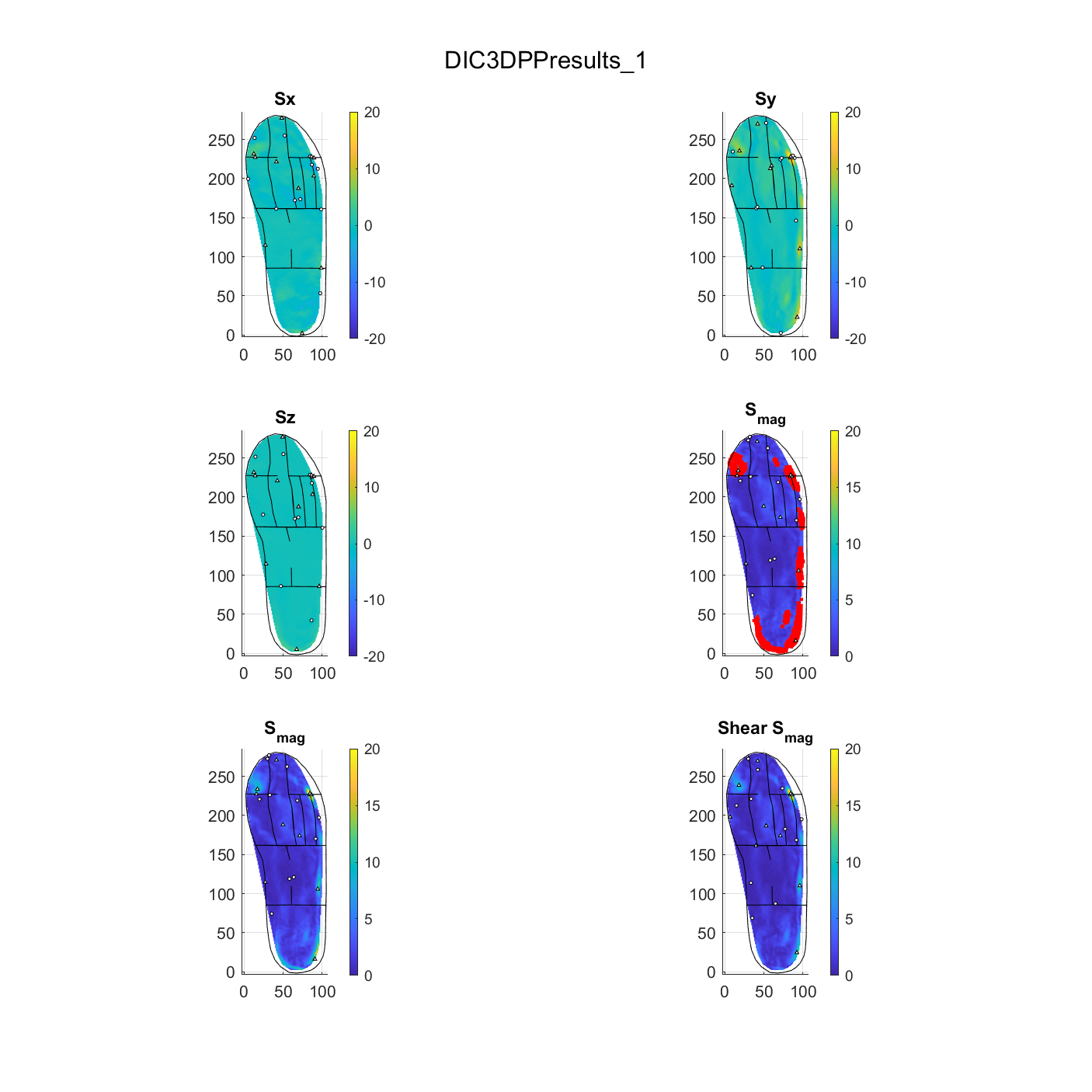 | 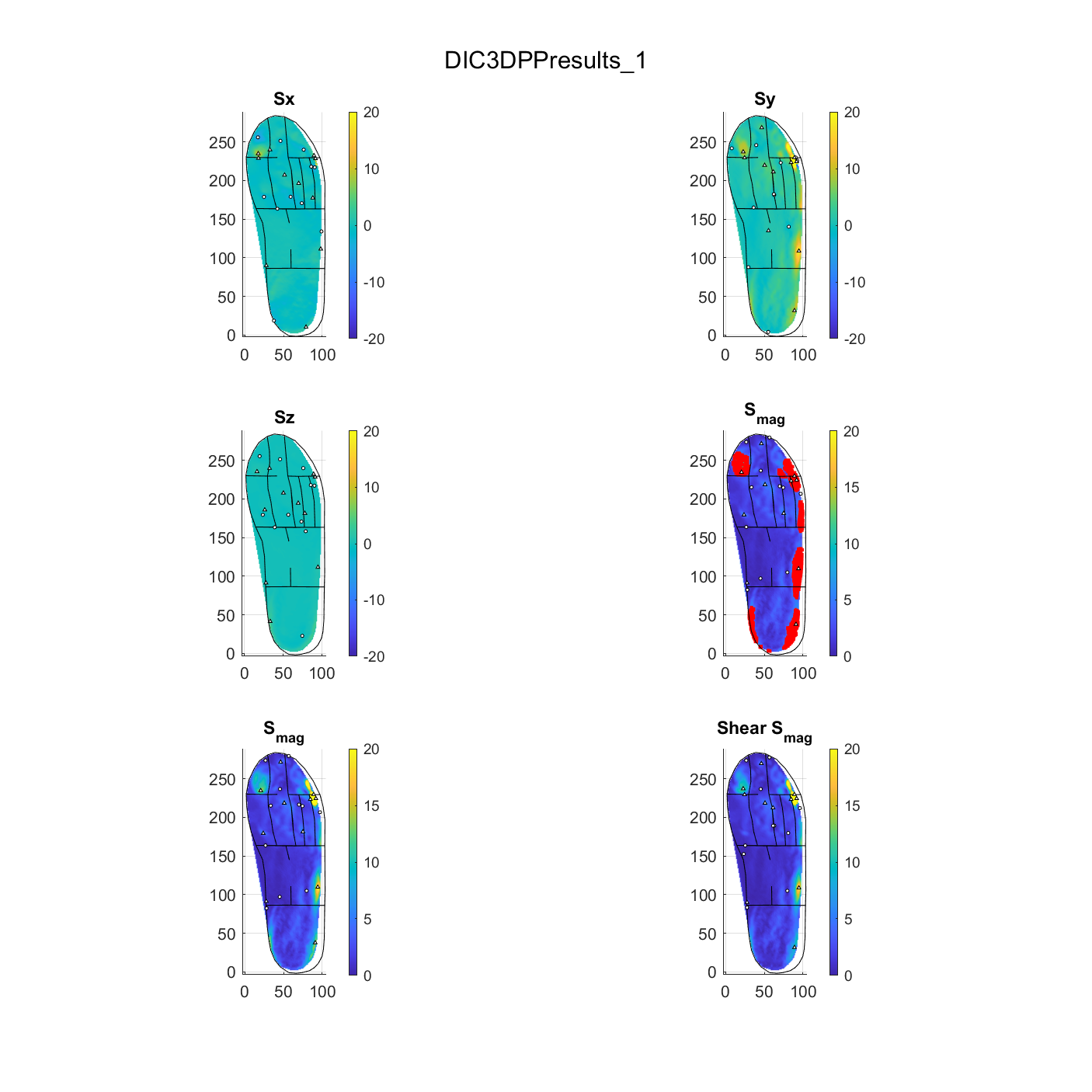 | 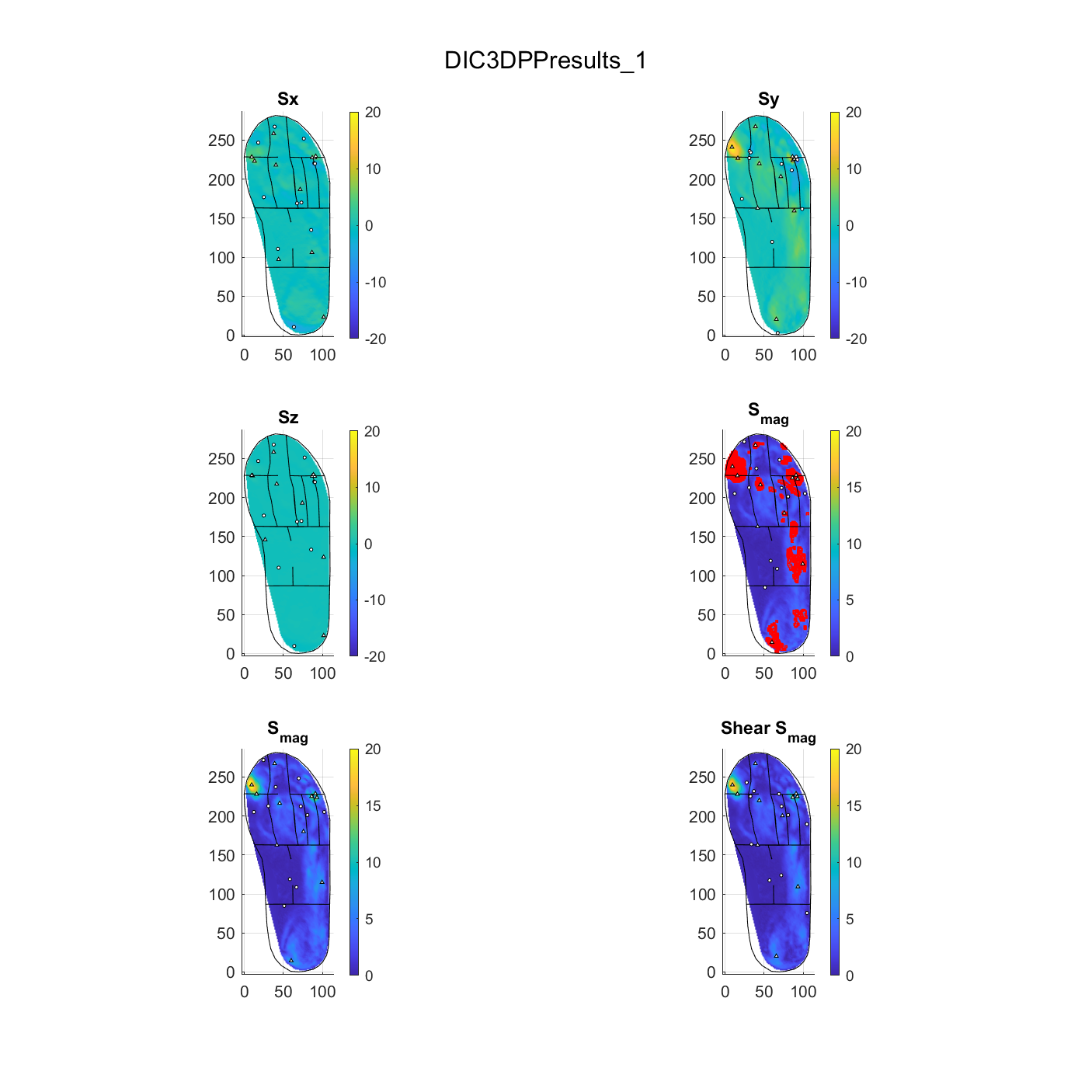 |
| P04 | 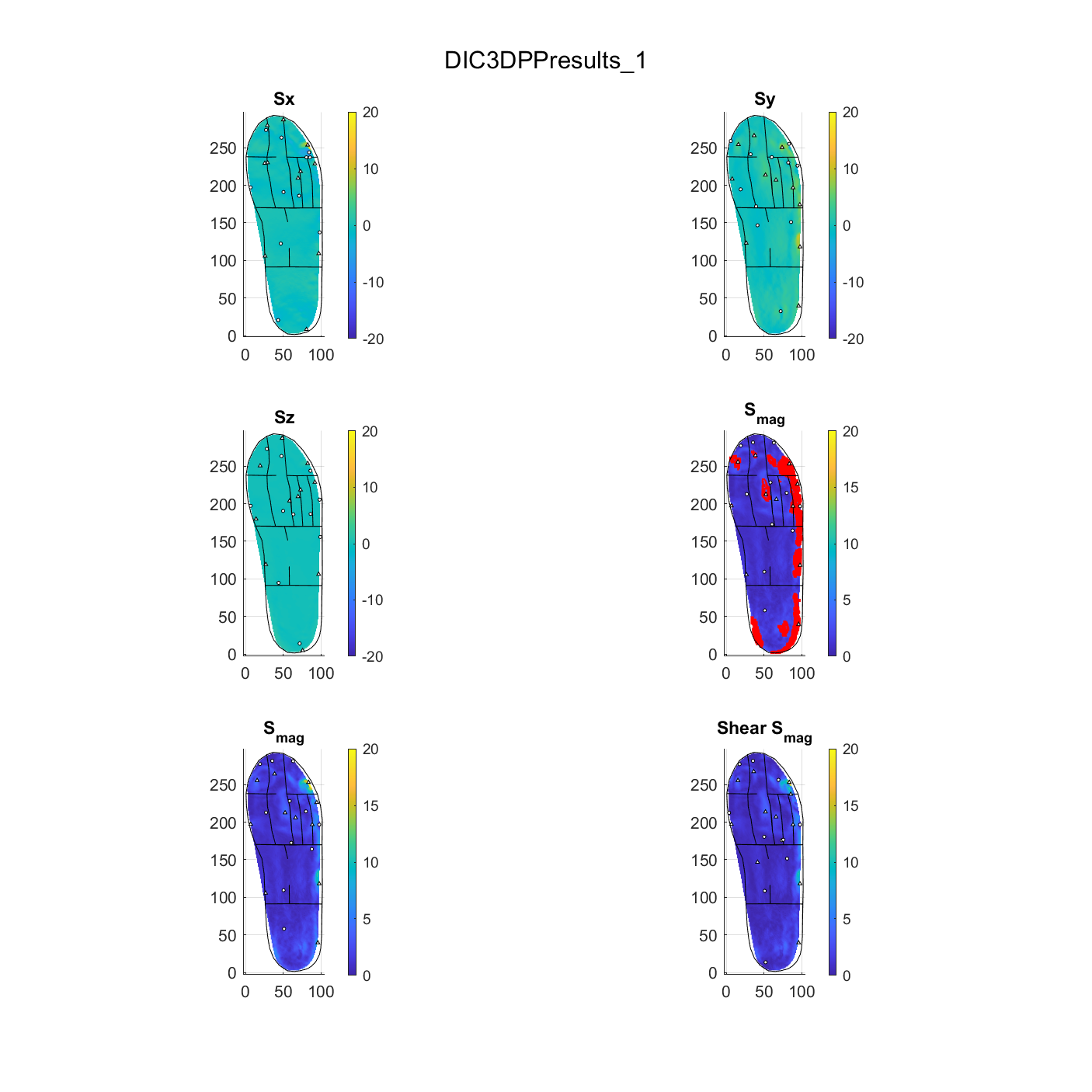 | 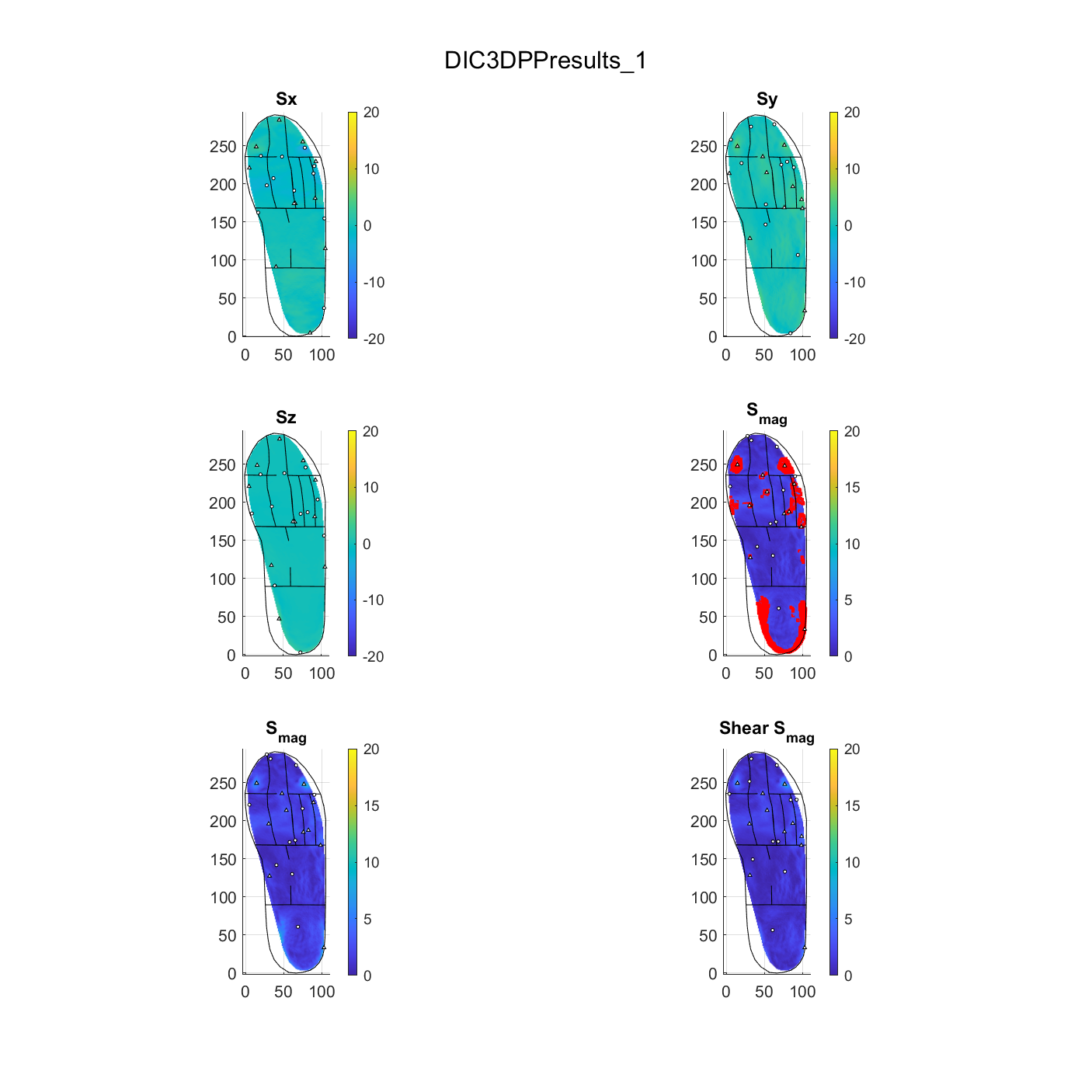 | 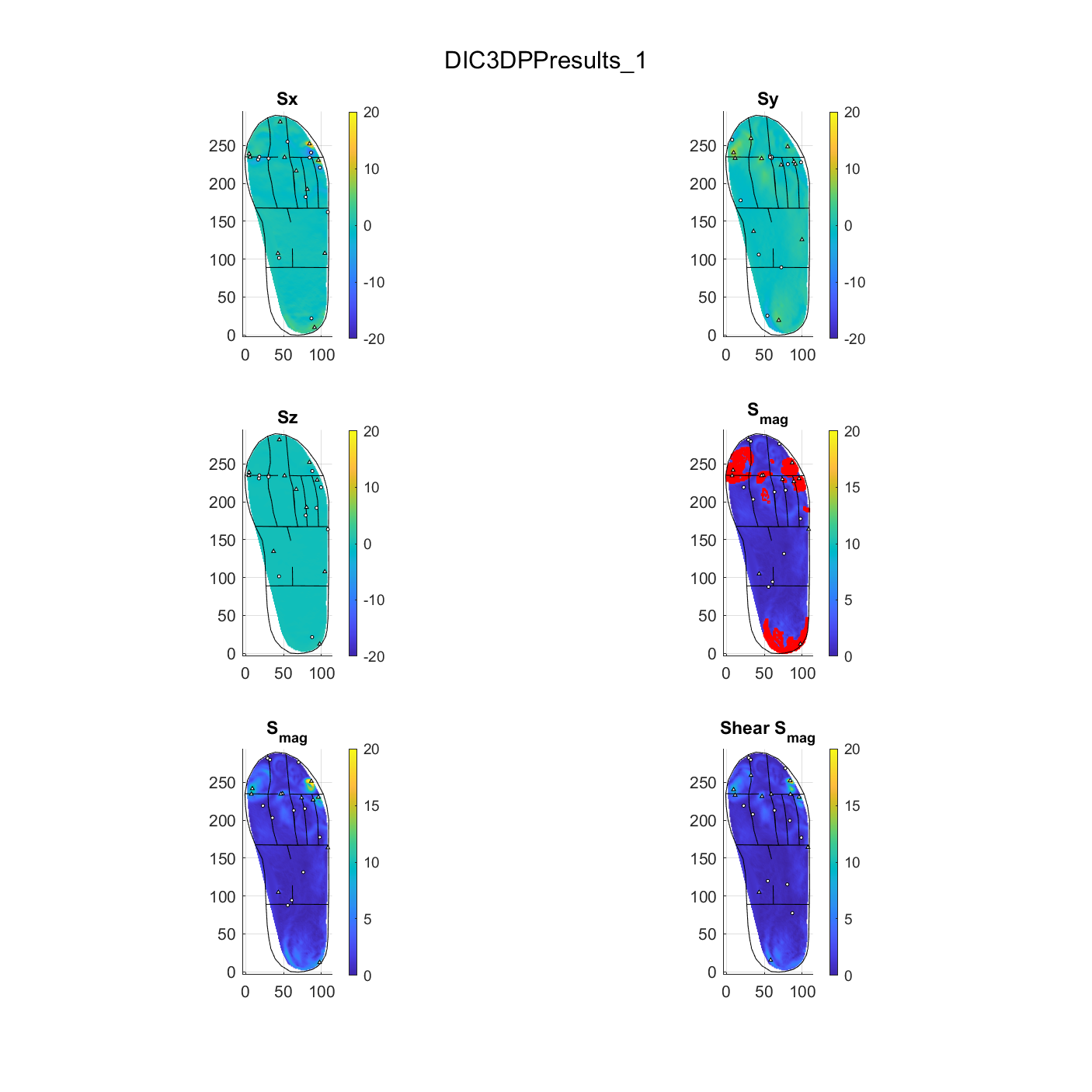 |
| P05 | 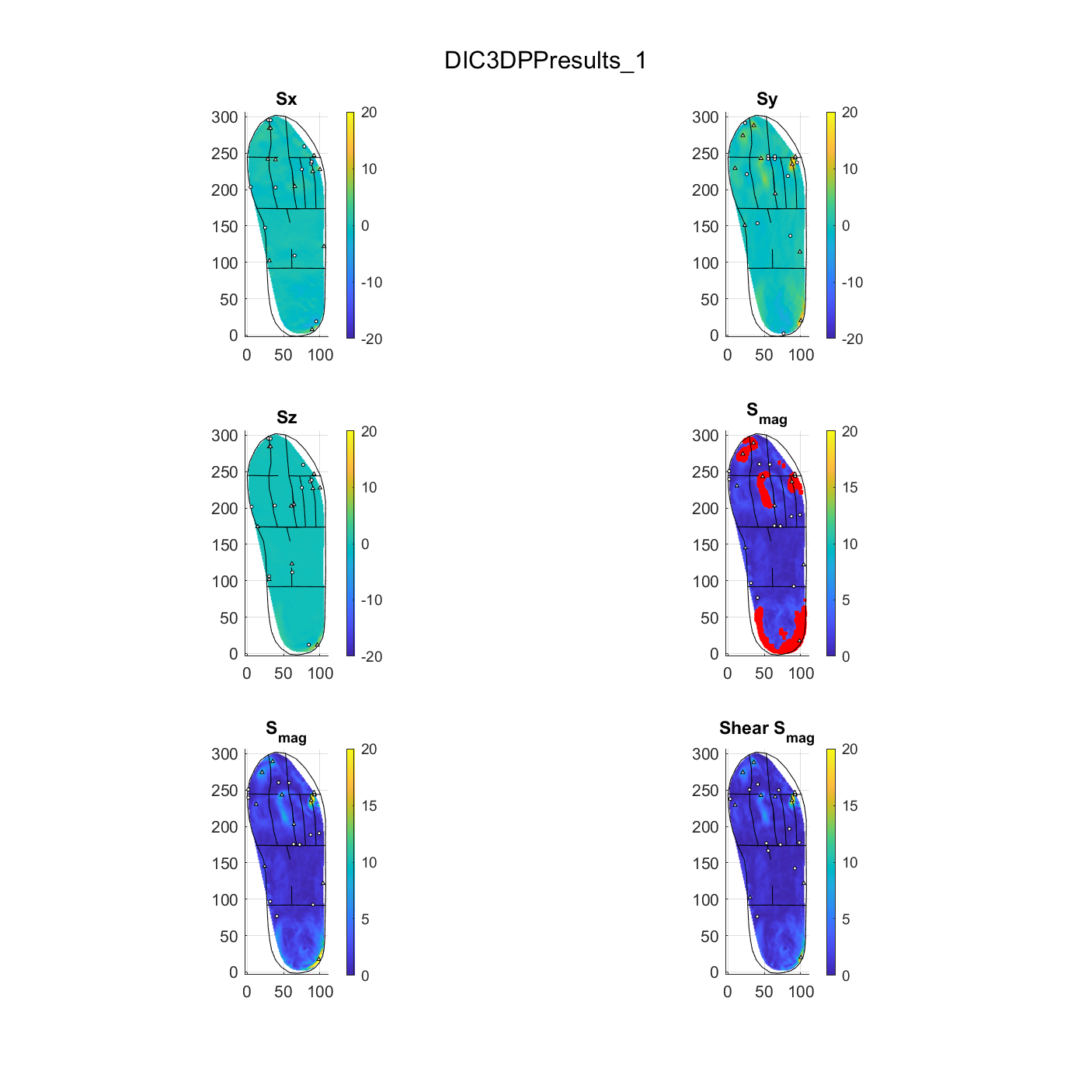 | 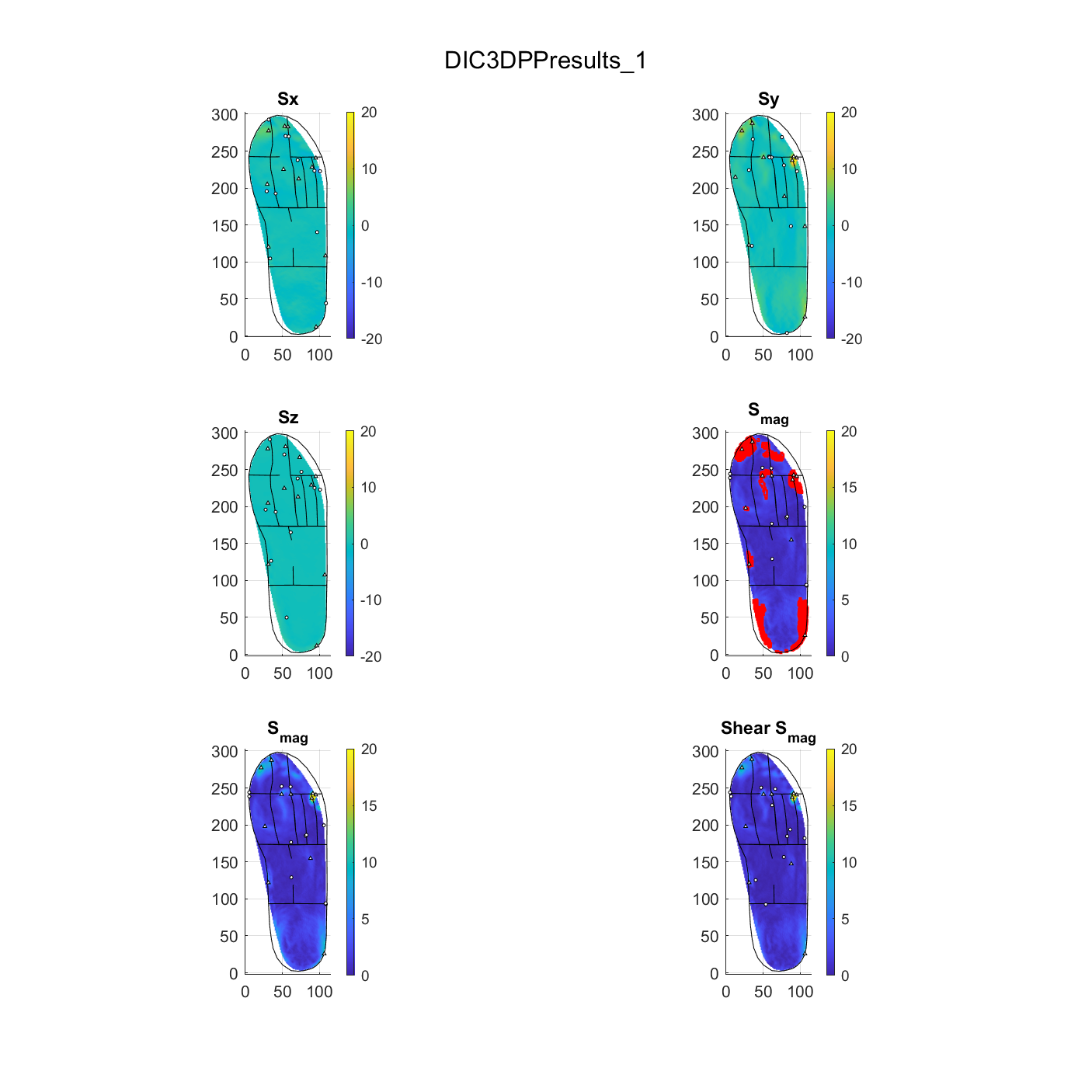 | 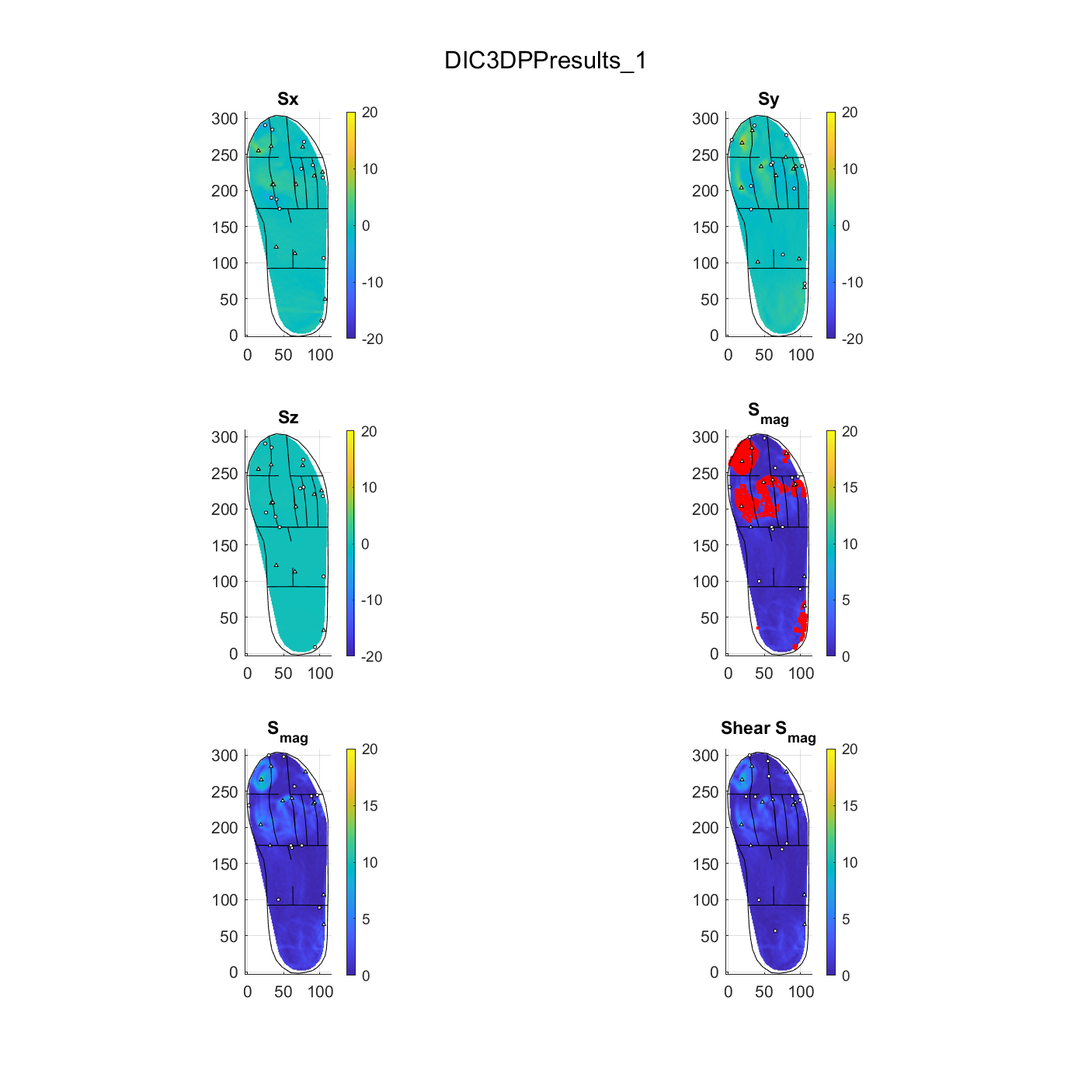 |

**Supplementary Figure 4.** Representative strain maps for S_X_, trial 2 across each participant and condition.

|  | **Low Stiffness** | **High Stiffness** | **STAMPS** |
| --- | --- | --- | --- |
|  | **Strain Map (S_X_)** | **Strain Map (S_X_)** | **Strain Map (S_X_)** |
| P01 | 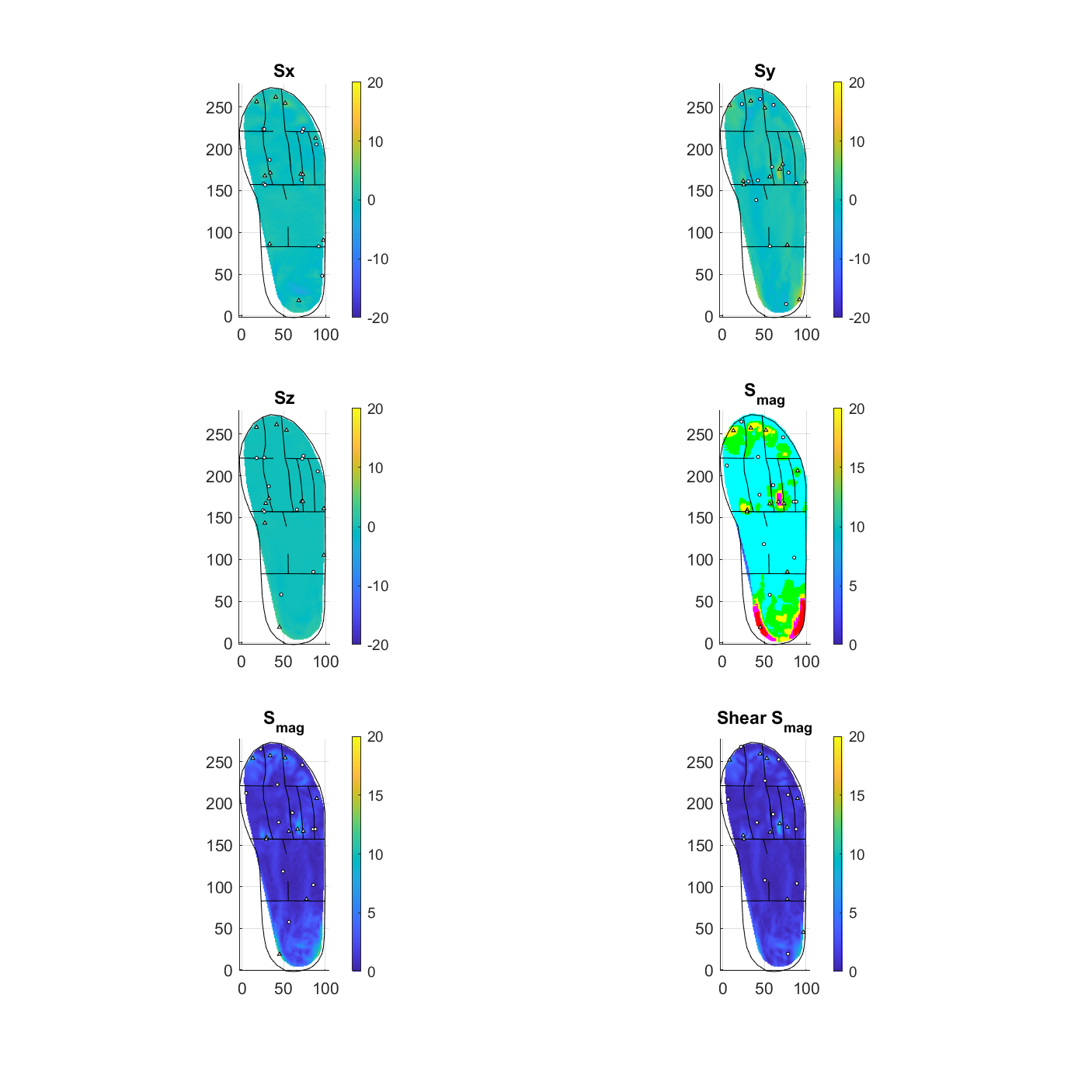 | 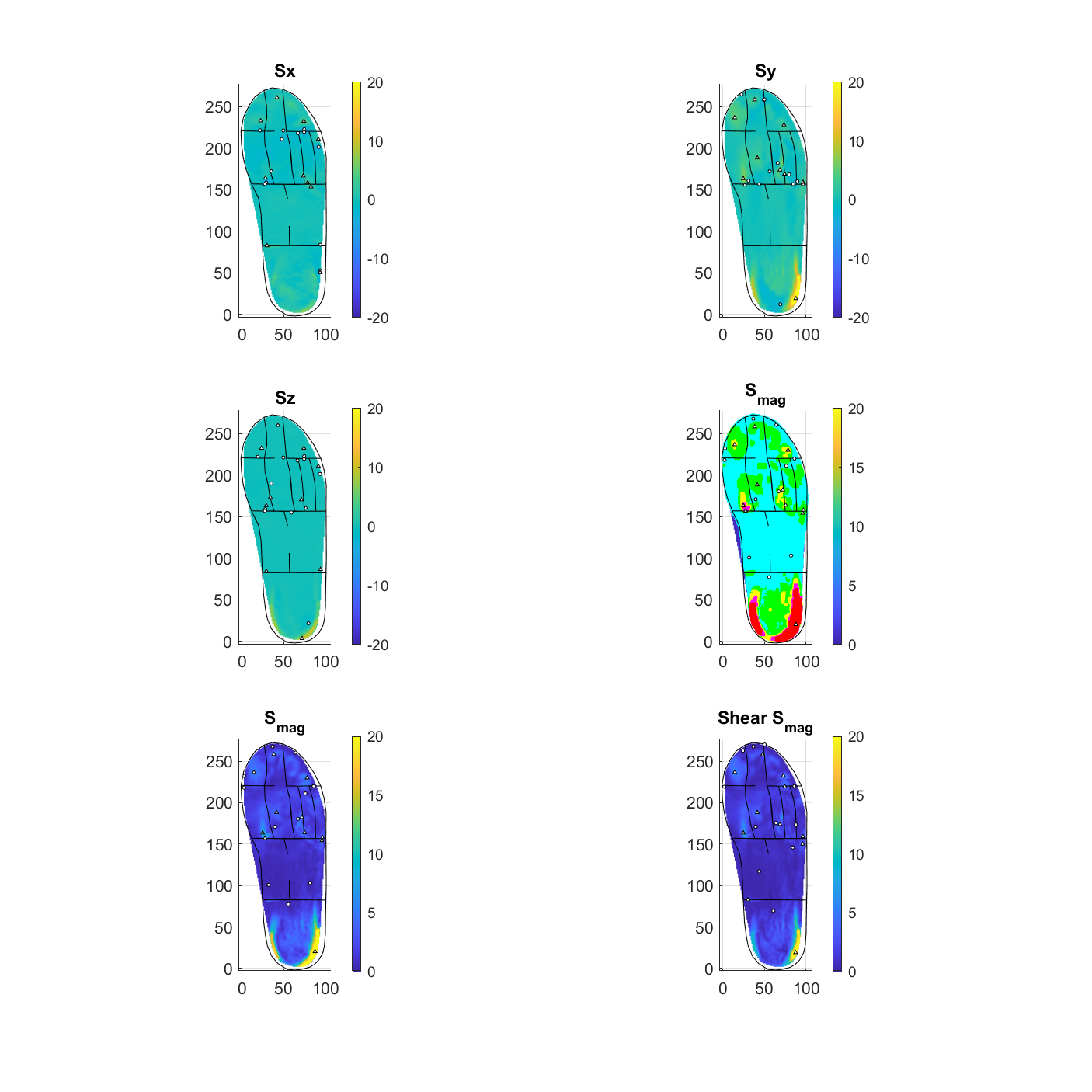 | 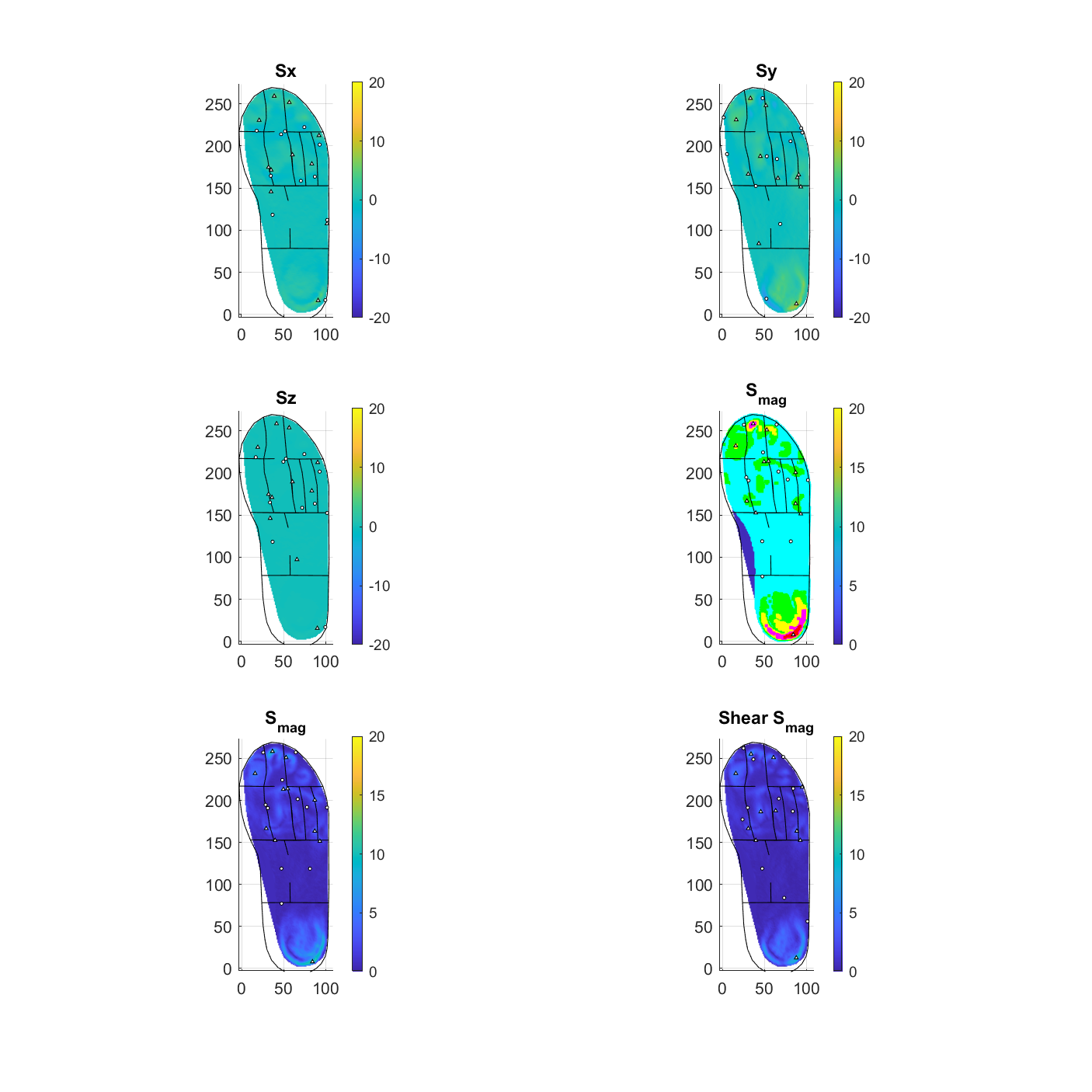 |
| P02 | 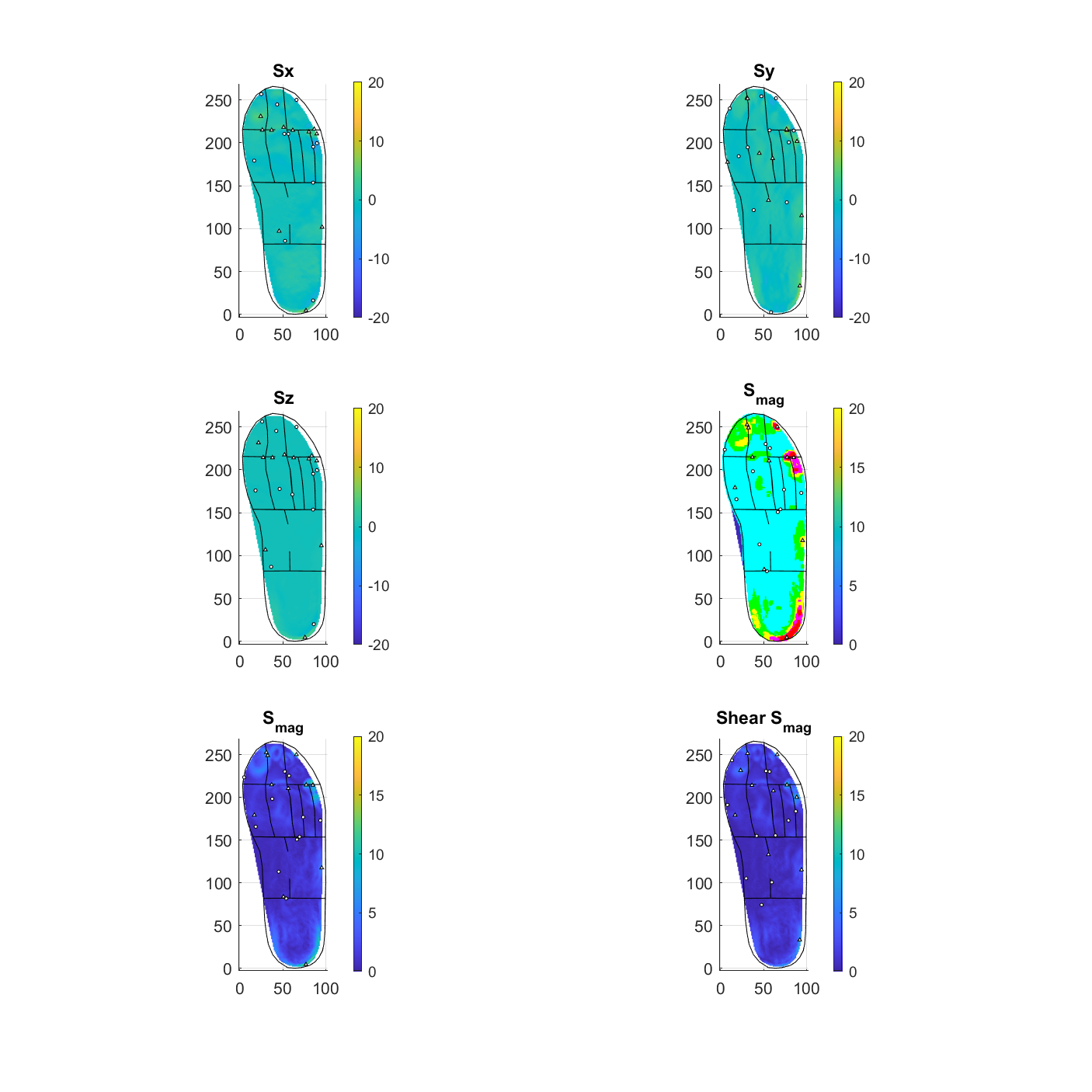 | 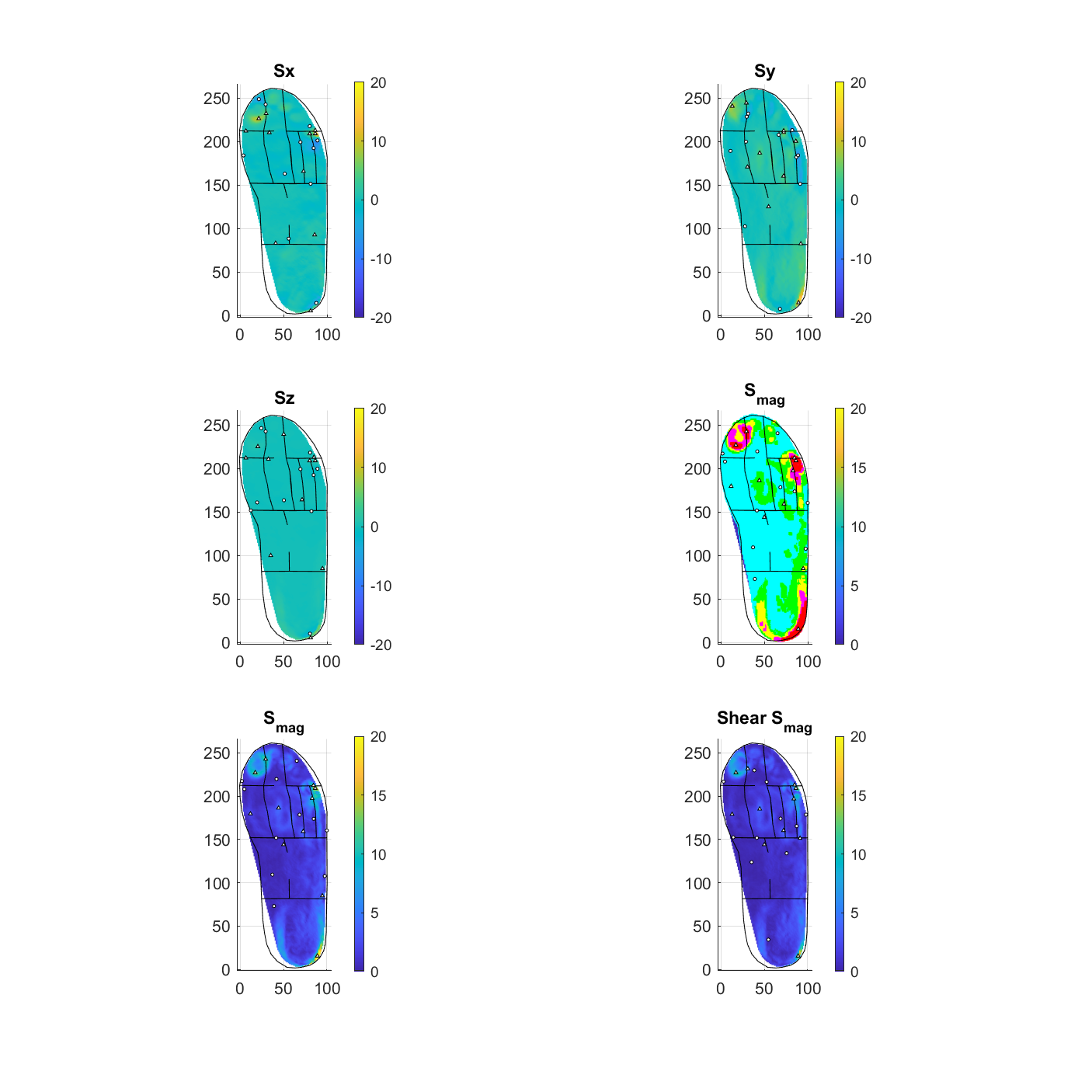 | 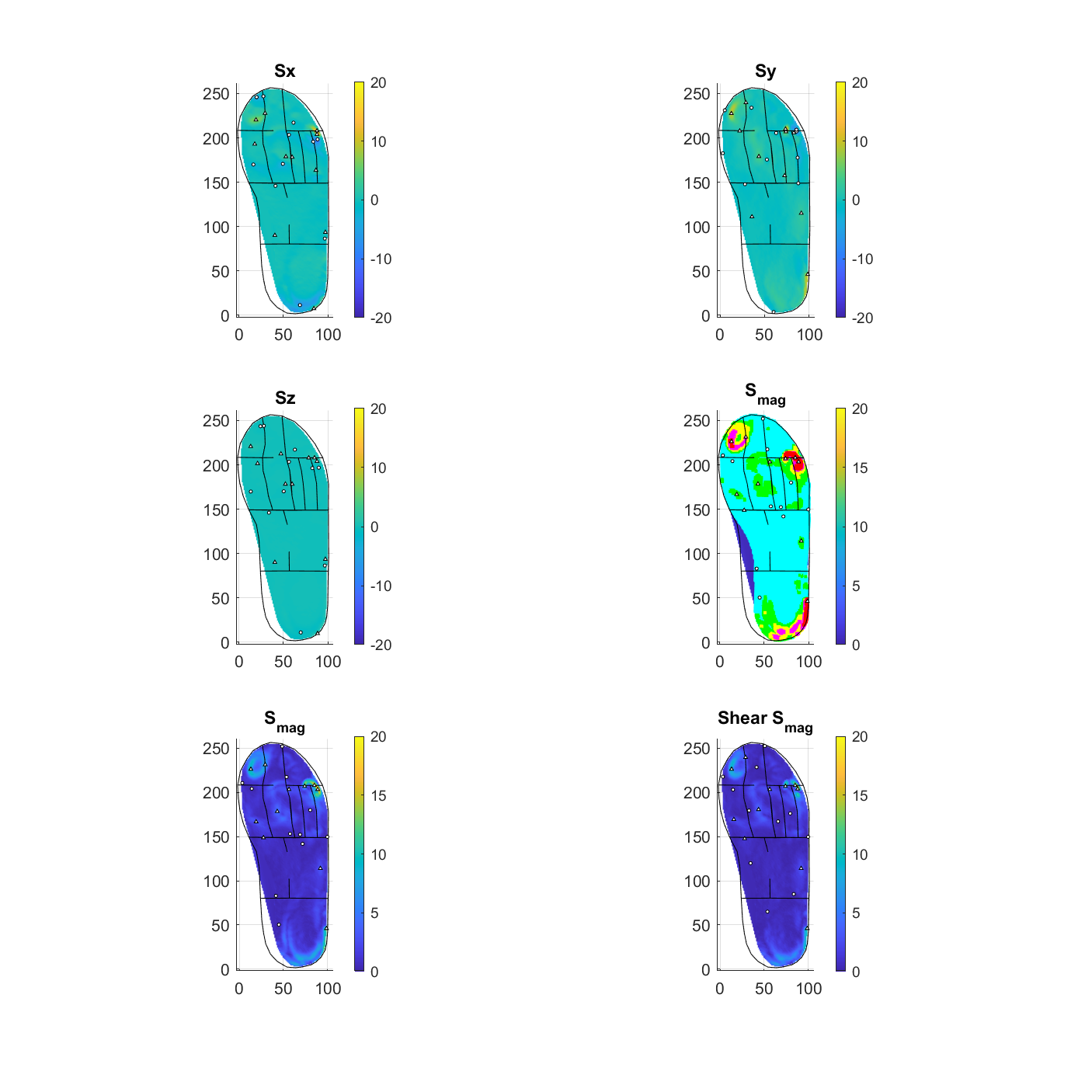 |
| P03 | 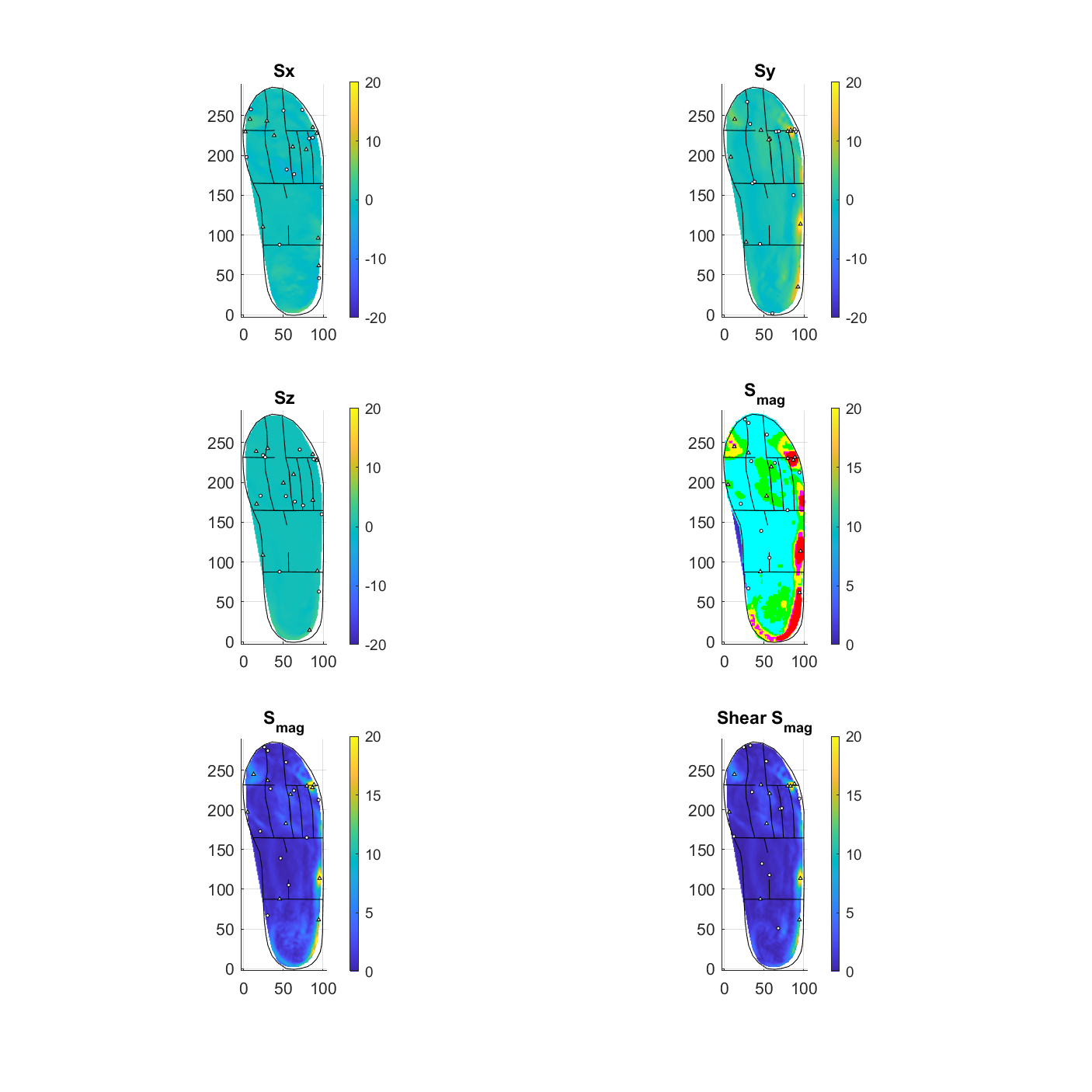 | 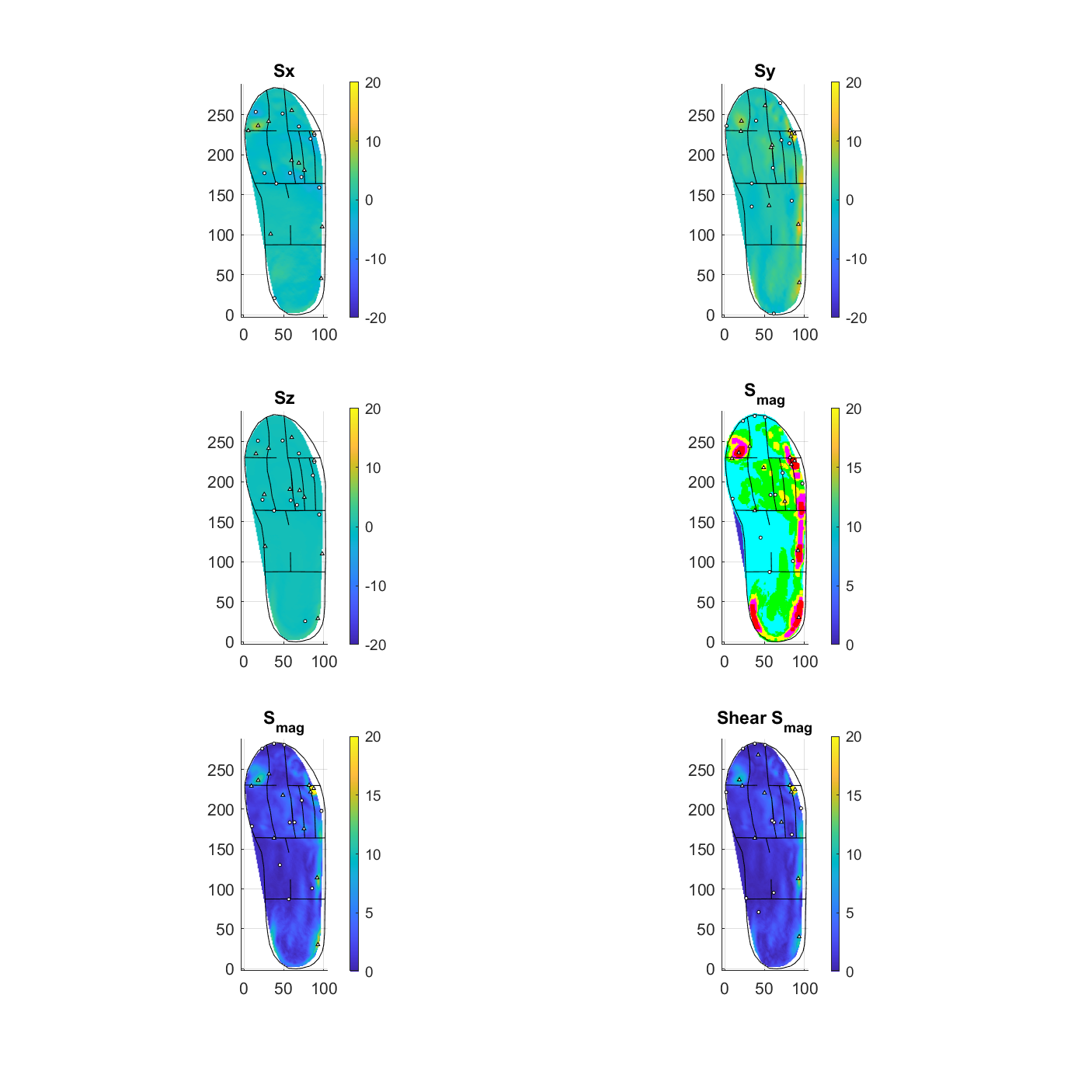 | 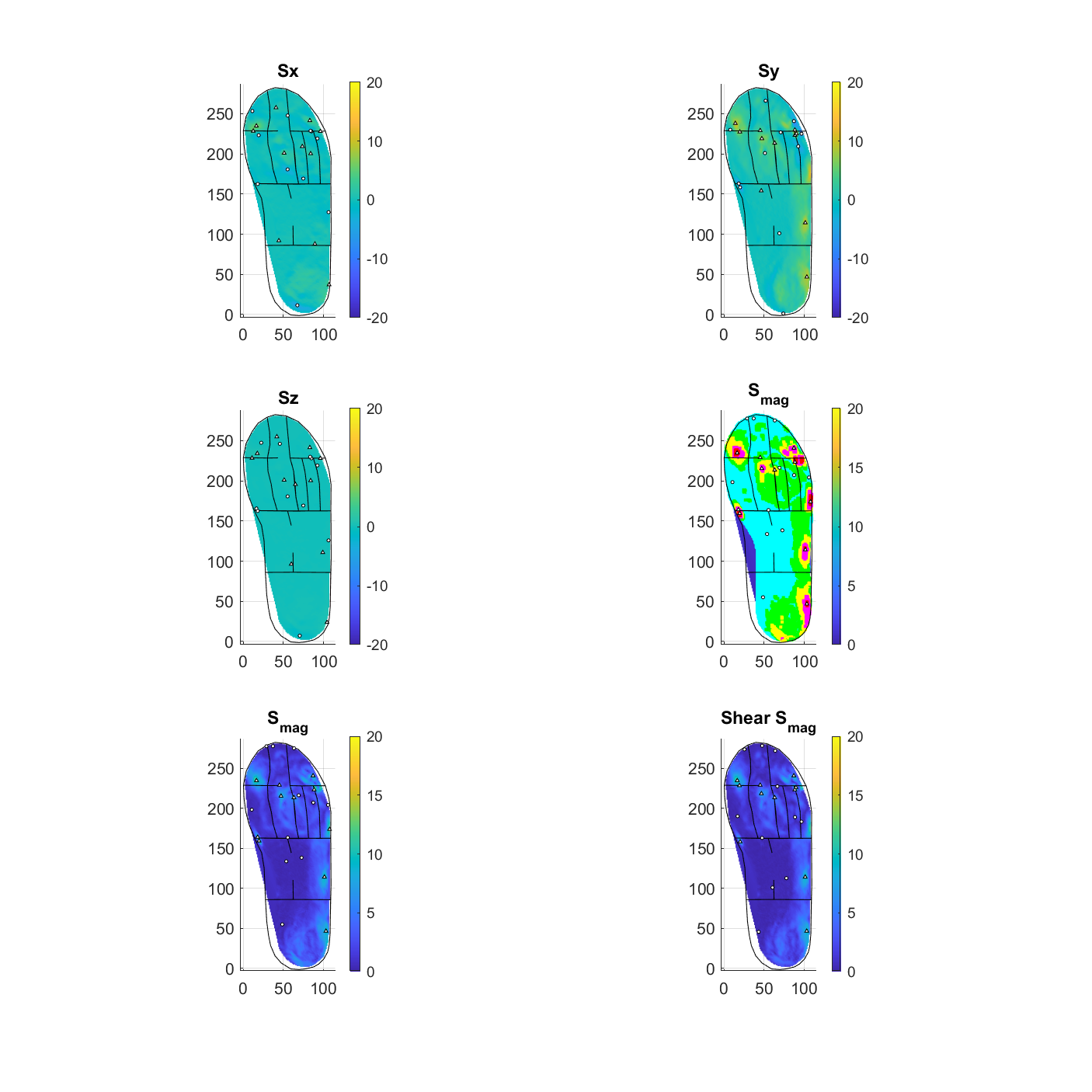 |
| P04 | 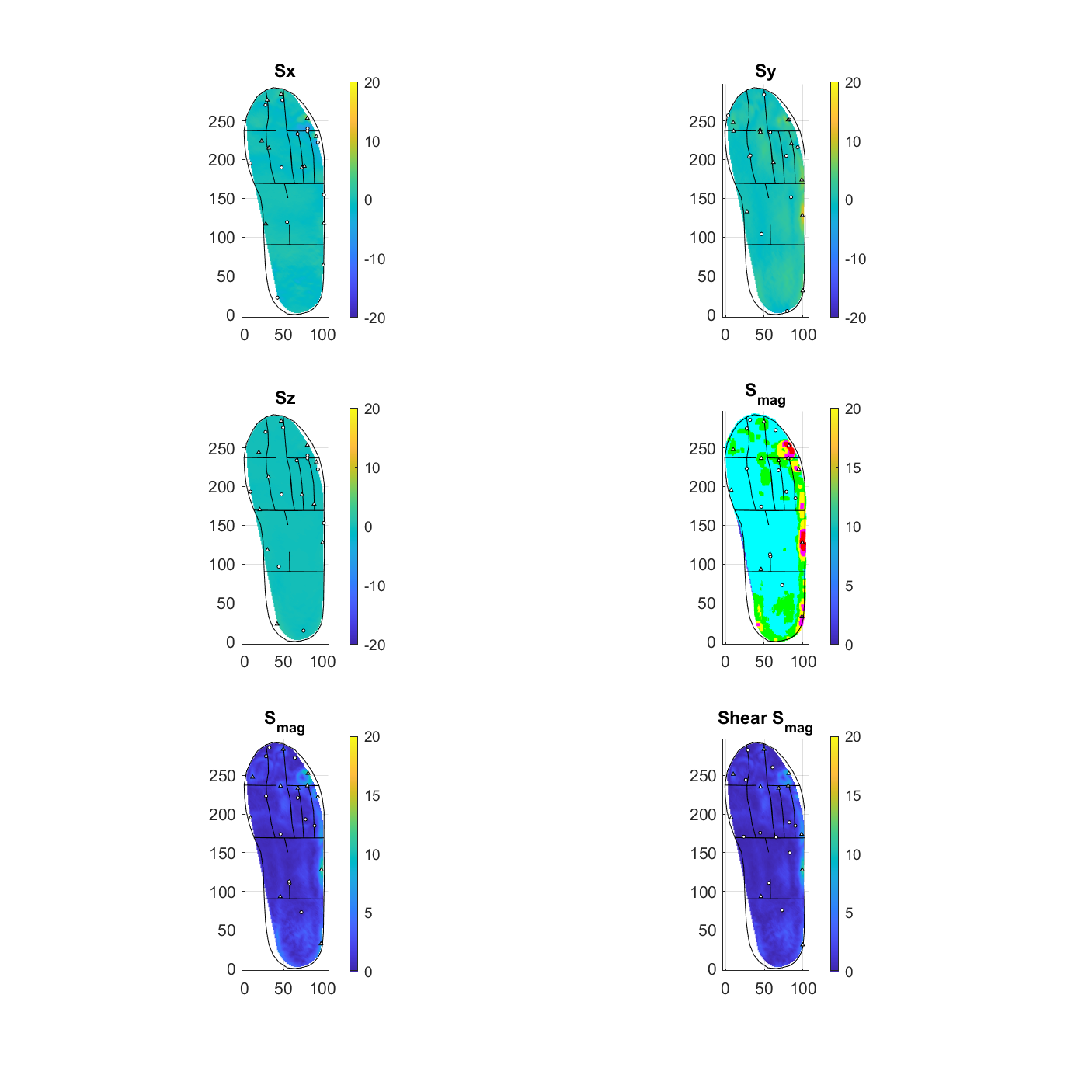 |  |  |
| P05 |  |  |  |

**Supplementary Figure 5.** Representative strain maps for S_X_, trial 3 across each participant and condition.

|  | **Low Stiffness** | **High Stiffness** | **STAMPS** |
| --- | --- | --- | --- |
|  | **Strain Map (S_Y_)** | **Strain Map (S_Y_)** | **Strain Map (S_Y_)** |
| P01 |  |  |  |
| P02 |  |  |  |
| P03 |  |  |  |
| P04 |  |  |  |
| P05 |  |  |  |

**Supplementary Figure 6.** Representative strain maps for S_Y_, trial 1 across each participant and condition.

|  | **Low Stiffness** | **High Stiffness** | **STAMPS** |
| --- | --- | --- | --- |
|  | **Strain Map (S_Y_)** | **Strain Map (S_Y_)** | **Strain Map (S_Y_)** |
| P01 |  |  |  |
| P02 |  |  |  |
| P03 |  |  |  |
| P04 |  |  |  |
| P05 |  |  |  |

**Supplementary Figure 7.** Representative strain maps for S_Y_, trial 2 across each participant and condition.

|  | **Low Stiffness** | **High Stiffness** | **STAMPS** |
| --- | --- | --- | --- |
|  | **Strain Map (S_Y_)** | **Strain Map (S_Y_)** | **Strain Map (S_Y_)** |
| P01 |  |  |  |
| P02 |  |  |  |
| P03 |  |  |  |
| P04 |  |  |  |
| P05 |  |  |  |

**Supplementary Figure 8.** Representative strain maps for S_Y_, trial 3 across each participant and condition.

|  | **Low Stiffness** | **High Stiffness** | **STAMPS** |
| --- | --- | --- | --- |
|  | **Strain Map (S_Z_)** | **Strain Map (S_Z_)** | **Strain Map (S_Z_)** |
| P01 |  |  |  |
| P02 |  |  |  |
| P03 |  |  |  |
| P04 |  |  |  |
| P05 |  |  |  |

**Supplementary Figure 9.** Representative strain maps for S_Z_, trial 1 across each participant and condition.

|  | **Low Stiffness** | **High Stiffness** | **STAMPS** |
| --- | --- | --- | --- |
|  | **Strain Map (S_Z_)** | **Strain Map (S_Z_)** | **Strain Map (S_Z_)** |
| P01 |  |  |  |
| P02 |  |  |  |
| P03 |  |  |  |
| P04 |  |  |  |
| P05 |  |  |  |

**Supplementary Figure 10.** Representative strain maps for S_Z_, trial 2 across each participant and condition.

|  | **Low Stiffness** | **High Stiffness** | **STAMPS** |
| --- | --- | --- | --- |
|  | **Strain Map (S_Z_)** | **Strain Map (S_Z_)** | **Strain Map (S_Z_)** |
| P01 |  |  |  |
| P02 |  |  |  |
| P03 |  |  |  |
| P04 |  |  |  |
| P05 |  |  |  |

**Supplementary Figure 11.** Representative strain maps for S_Z_, trial 3 across each participant and condition.

## Boxplots

**Supplementary Figure 12.** Boxplot demonstrating global distribution of peak S_MAG_ across all participants for the low stiffness, high stiffness and STAMPS conditions.

**Supplementary Figure 13.** Boxplot demonstrating distribution of peak S_MAG_ across all participants for the low stiffness, high stiffness and STAMPS conditions at the hallux.

**Supplementary Figure 14.** Boxplot demonstrating distribution of peak S_MAG_ across all participants for the low stiffness, high stiffness and STAMPS conditions at the 2^nd^ toe.

**Supplementary Figure 15.** Boxplot demonstrating distribution of peak S_MAG_ across all participants for the low stiffness, high stiffness and STAMPS conditions at the toes 3-5.

**Supplementary Figure 16.** Boxplot demonstrating distribution of peak S_MAG_ across all participants for the low stiffness, high stiffness and STAMPS conditions at the MTH1.

**Supplementary Figure 17.** Boxplot demonstrating distribution of peak S_MAG_ across all participants for the low stiffness, high stiffness and STAMPS conditions at the MTH2.

**Supplementary Figure 18.** Boxplot demonstrating distribution of peak S_MAG_ across all participants for the low stiffness, high stiffness and STAMPS conditions at the MTH3.

**Supplementary Figure 19.** Boxplot demonstrating distribution of peak S_MAG_ across all participants for the low stiffness, high stiffness and STAMPS conditions at the MTH4.

**Supplementary Figure 20.** Boxplot demonstrating distribution of peak S_MAG_ across all participants for the low stiffness, high stiffness and STAMPS conditions at the MTH5.

**Supplementary Figure 21.** Boxplot demonstrating distribution of peak S_MAG_ across all participants for the low stiffness, high stiffness and STAMPS conditions at the medial midfoot.

**Supplementary Figure 22.** Boxplot demonstrating distribution of peak S_MAG_ across all participants for the low stiffness, high stiffness and STAMPS conditions at the lateral midfoot.

**Supplementary Figure 23.** Boxplot demonstrating distribution of peak S_MAG_ across all participants for the low stiffness, high stiffness and STAMPS conditions at the heel.
